# The impact of social marketing practices in promoting digital etiquette: a descriptive and analytical study on a sample of Fallujah University students

**DOI:** 10.12688/f1000research.173990.1

**Published:** 2026-04-18

**Authors:** Reham Moatasem Hamza, Ahmed Abbas Hammadi

**Affiliations:** 1 Department of Public Administration, College of Administration and Economics, University of Fallujah, Iraq; 2 Department of Business Administration, College of Administration and Economics, University of Fallujah, Iraq

**Keywords:** social marketing practices, digital etiquette.

## Abstract

**Background:**

The research aims to know the role of social marketing practices in enhancing digital etiquette at the University of Fallujah, through a descriptive and analytical study on a sample of students amounting to (215) students. The research problem was the deficiency in some students' commitment to digital etiquette. The importance of the study is highlighted in analyzing the impact of social marketing tools on raising awareness of ethical values on the Internet and elsewhere. The research recommends the necessity for Iraqi universities to adopt innovative social marketing practices to enhance digital responsibility, and develop technological infrastructure and human resources in line with the modern digital environment.

**Methodology:**

The research followed the descriptive analytical approach, and the data were collected via a questionnaire. The data were analyzed using the program (SPSS V.24) based on arithmetic means, standard deviations, the simple correlation coefficient (Pearson), and multiple linear regression testing to verify the influence relationships between the study variables.

**Results:**

Analytical results indicate that the university regularly adopts social marketing tools and activates its four dimensions (social product, social pricing, social promotion, and social distribution), which has had a positive impact on digital transformation. The data showed an increase in the level of commitment to digital etiquette among students by (25%) according to the approved digital behavior standards, with a significant moral impact (p < 0.05) of social marketing practices in promoting positive digital behaviors and reducing irresponsible behaviors.

**Conclusions:**

Social marketing practices play an important role in raising awareness among Fallujah University students about the importance of responsible digital behavior. The use of various methods such as awareness videos, infographics, and direct interaction with students also helped deliver ethical messages more effectively. The study confirms that social marketing is a real catalyst for social change in the digital environment.

## Introduction

The world today is witnessing rapid transformations led by modern technology and the digitization of societies, which has made digital behavior an essential element in shaping the behavior of individuals ( Causio et al., 2025:4). Internet technologies have become an integral part of daily life, providing broad possibilities for communication, but at the same time they require high responsibility and commitment to digital etiquette as a socio-cultural phenomenon resulting from the development of digital civilization This development has contributed to reshaping traditional social values and norms, and has highlighted the need for digital etiquette as a normative and ethical regulator of communication in the virtual environment ( Issembayeva & Seyitkazy, 2025:11-12).

In light of the Iraqi Ministry of Higher Education and Scientific Research's move towards digitization, local university education is witnessing a rapid shift towards a digital environment, which has produced new practices and behaviors among students within e-learning spaces. Despite the opportunities this shift provides for interaction, creativity, and knowledge exchange, it may reveal aspects of the shortcomings in some students' commitment to digital etiquette Whether in communicating via academic platforms or in using electronic resources in a responsible and ethical manner, such as publishing inappropriate comments, sharing information without permission, etc. The US Development Bank report (2025) indicates that digital behaviors require users and developers to practice conscious and responsible behavior that protects users from misuse of technology, such as cyberbullying and exploitation of personal data, and in return promotes the development of technologies that support and enhance social welfare and human rights ( Latorre et al., 2025:7). The World Health Organization also directly confirmed this problem, as it indicated in its report issued in 2025 that: «Research has reinforced the existence of multiple online risks to the mental health, physical health, and well-being of young people, with a strong consensus that the stages of biological and cognitive development of children and adolescents make them particularly vulnerable to online harm» ( WHO, 2025, 2). This reflects the complex nature of digital behavior and highlights the need for effective interventions that promote awareness and safe use of technology.

In this context, the importance of studying social marketing practices emerges as an influential tool capable of bringing about positive behavioral changes that lead to improving the well-being of the individual and benefit society as a whole ( Galiano-Coronil et al., 2024), as its essence is based on achieving the public good and modifying negative behaviors based on behavioral, psychological and marketing sciences to solve social problems ( Shekhar and Venugopal, 2025:1-3) Khatti-Dizabadi et al. (2024:155) agreed that social marketing practices represent (social product, social pricing, social distribution, social promotion). A social product may be a service, idea, or behavioral change, such as digital stress relief initiatives, smartphone overuse reduction systems, and digital wellbeing programs ( Arenas-Escaso et al., 2024:1-5; Orzikulova et al., 2024:2-3), as for the social price, it is represented by the effort made to adopt the new behavior ( Kegoro, 2020:91), such as self-control to use the phone, which is an influential factor in reducing over-reliance on technology ( Li et al., 2025:2), ( Fayyaz, 2022:19) indicated that place means providing goods, services, or the appropriate environment that facilitates the adoption of the new behavior, as for ( Kegoro, 2020:9). It focused on the fact that the dimension of social promotion is measured by indicators such as adaptability and consumption time, ensuring that interventions reach the target group effectively. For example, Apple and Google have introduced a range of features and apps such as SPACE and GDW to help reduce distraction and enhance the user's digital well-being ( Almourad et al., 2021:1-3). Social promotion focuses on raising awareness by using media and awareness messages to promote healthy digital behaviors through preventive digital applications and interventions ( Saud, 2022:128; Akbar et al., 2023:1; Zhao et al., 2024:3; Wu et al., 2025).

In today's interconnected world, understanding digital etiquette is essential to maintaining safe and respectful online interactions ( Ab Wahab, 2024:2), and after reviewing many studies, research and scientific literature related to the concept of digital etiquette such as the study of ( Abdul Tawab, 2020; Al-Shehri, 2021; Haroun and Hassouna, 2022), The researchers summarized it in five basic dimensions that represent the main axes on which digital behavior is built. The technological dimension is represented in the individual's awareness of using information technology, and his mastery of the rules of digital communication such as writing email and using platforms in a professional manner, while the ethical dimension is concerned with adhering to ethical rules derived from religion, law, and societal norms, while respecting privacy and the rights of others, avoiding abuse, protecting personal information, and reviewing content before publishing it to ensure that it is not misused ( Abdel Tawab, 2020; Al-Shehri, 2021; Haroun and Hassouna, 2022). The social dimension includes polite behaviors that regulate individuals' interaction within the digital environment, such as taking responsibility, effective communication, respecting other opinions, and positive participation that reflects the spirit of cooperation within the digital community ( Abdul Tawab, 2020; Al-Shehri, 2021; Haroun and Hassouna, 2022). Social media is a tool for instilling social values and teaching acceptable interaction styles ( Neumann & Rhodes, 2023:2), but it can present challenges such as overreliance and digital bullying in its various forms, making digital moral and social education essential to ensure respectful and responsible interaction within the digital space ( Rini et al., 2023:107). The health dimension includes the behaviors that individuals should practice while using digital technology, as part of digital etiquette, such as healthy sitting, organizing the duration of use, and paying attention to basic meals to ensure a balance between healthy living and digital use. This dimension is linked to awareness of the risks resulting from excessive use of technology, as it may lead to depression, anxiety, isolation, and sleep disorders. The World Health Organization has classified Internet addiction as one of the global problems affecting sleep, motivation, memory, relationships, and quality of life, due to its similarity to addiction to harmful substances ( Dresp-Langley & Hutt, 2022:2-4). To address these effects, healthy behavior change (HBC) interventions have emerged that adopt psychological and behavioral approaches to improve diet, physical activity, sleep, and reduce the use of harmful substances. Although traditional interventions are effective, they are expensive, making digital alternatives more scalable ( Sawyer et al., 2023:6965). Digital health awareness has also emerged as a new determinant of health, enabling individuals to use digital technologies to access health information and employ it in problem solving, reflecting the integration of health awareness with digital awareness ( Qiu et al., 2025:2). The security dimension is concerned with protecting accounts, personal information, and files from any unauthorized interference or access ( Haroun and Hassouna, 2022, p. 250) and is increasingly important in the digital higher education environment, which requires advanced awareness of cybersecurity to ensure the safe use of technologies. Information security is based on the principles of the Triangle (CIA): confidentiality, integrity, availability, non-repudiation and credibility as elements that enhance digital trust. Effective awareness of information security based on understanding personal protocols and responsibilities is also an essential element for protecting organizations, and is not limited to measuring awareness, but includes evaluating the impact of security initiatives in reducing human errors and promoting preventive behaviors ( Bognár & Bottyán, 2024:1–2). In light of the above, it is important and necessary to conduct this research in a new local and digital environment and among new users, namely students of the University of Fallujah in Iraq, with the aim of building responsible digital awareness. Here, a major important question arises that can be raised: (What is the role of social marketing practices in enhancing digital behavior?), and how, through these practices, students can be directed towards using technology and digital platforms in a responsible, healthy and safe manner, which contributes to developing a digital educational environment based on respect, awareness and commitment to digital etiquette.

## Methodology

### Type, design and scope

This study relied on the descriptive analytical approach, due to its ability to describe and analyze the phenomena under study. First, the research variables represented in social marketing practices (social product, social pricing, social promotion, social distribution) and digital etiquette were described in its dimensions (technological dimension, ethical dimension, social dimension, health dimension, security dimension), through a questionnaire form designed for this purpose. The data were then analyzed statistically using appropriate statistical methods to extract indicators and interpret relationships between variables, allowing for a deeper understanding and clear vision of the current reality of the Iraqi universities under study.

### Community and sample

The research community consists of all students of Fallujah University, as they represent the target group for studying the impact of social marketing practices in promoting digital etiquette. A comprehensive inventory method was adopted in selecting the sample, so that the sample included a large number of students to ensure the inclusion of all units related to the subject of the study and to obtain accurate data that reflects the actual reality of digital behavior among students within the university.

### Procedures

According to the research design, the research followed logical and systematic steps, which facilitated the solution of the research problem. The most important research steps included the following points: conceptualizing the idea, stating the problem, designing the research, selecting the sample, collecting data, analyzing it, interpreting it, and discussing the results.

### Data collection techniques and tools

Considering the dimensions and indicators of the study, the questionnaire tool was adopted as the main means of collecting data. This tool was developed to include the social marketing practices variable consisting of (24) items, distributed over its dimensions, with the adoption of the five-point Likert scale to determine the level of response. As for the digital etiquette variable, it included (30) paragraphs, distributed according to its dimensions, and using the same scale. The questionnaire was applied to the study sample members with the aim of obtaining the data necessary for statistical analysis.

### Analyze the results

The study aimed to determine the impact relationship between social marketing practices and digital etiquette for a sample of Fallujah University students. The study was conducted based on questionnaire data, and according to the research variables, the following was observed:

### First: Describe the opinions of the researched sample

This paragraph will describe the identifying information of the respondents, as shown in Table 1 below:

1- **Demographic information**
  ➢ **Gender**: Males accounted for 55% of the respondents, while females accounted for 54%. This demonstrates that the representation of males and females is equal at the university under study, as outlined in Figure 1.
  ➢ **Age**: It is noted that the age group (20 years and younger) obtained a percentage of 4.7%, while the age group (25-21 years) obtained a percentage of 47%, the age group (under 30 years) obtained a percentage of 32.1%, and finally the age group (30 years and older) obtained a percentage of 16.2% for respondents, as outlined in Figure 2.
  ➢ **Academic qualification**: It is noted that the preparatory academic qualification obtained a percentage of 28.8%, the bachelor's academic qualification obtained a percentage of 46%, while the master's academic achievement obtained a percentage of 25.2% for respondents.
The identifying information can be explained conceptually, as outlined in Figure 3.
2- **Describe and diagnose the study variables**
The process of describing and diagnosing the study variables at the macro level and in terms of their dimensions is one of the important steps to determine the level of orientation and tendencies of the researched individuals towards those variables and dimensions. These tendencies are usually measured through the percentage of agreement and the relative importance index (Relative importance index (RII)). This index is used to know the relative importance of the paragraphs and dimensions of the study from the point of view of the research sample in the researched organization. Note that the value of this indicator falls between (0≤RII≤1) and its value can be classified into five levels according to the five-point Likard scale adopted in our study, as shown in Table 2.

**Table 1.** Description of the researched individuals.

Description	Division or category	Number	Ratio
**Gender**	**Male**	**118**	**55%**
	**Female**	**97**	**45%**
	**Total**	**215**	**100%**
**Age**	**20 years and younger**	**10**	**4.7%**
	**25-21 years**	**101**	**47%**
	**Under 30 years old**	**69**	**32.1%**
	**30 years or older**	**35**	**16.2%**
	**Total**	**215**	**100%**
**Academic qualification**	**Preparatory**	**62**	**28.8%**
	**Undergraduate**	**99**	**46%**
	**Master's**	**54**	**25.2%**
	**Total**	**215**	**100%**

**Figure 1.**
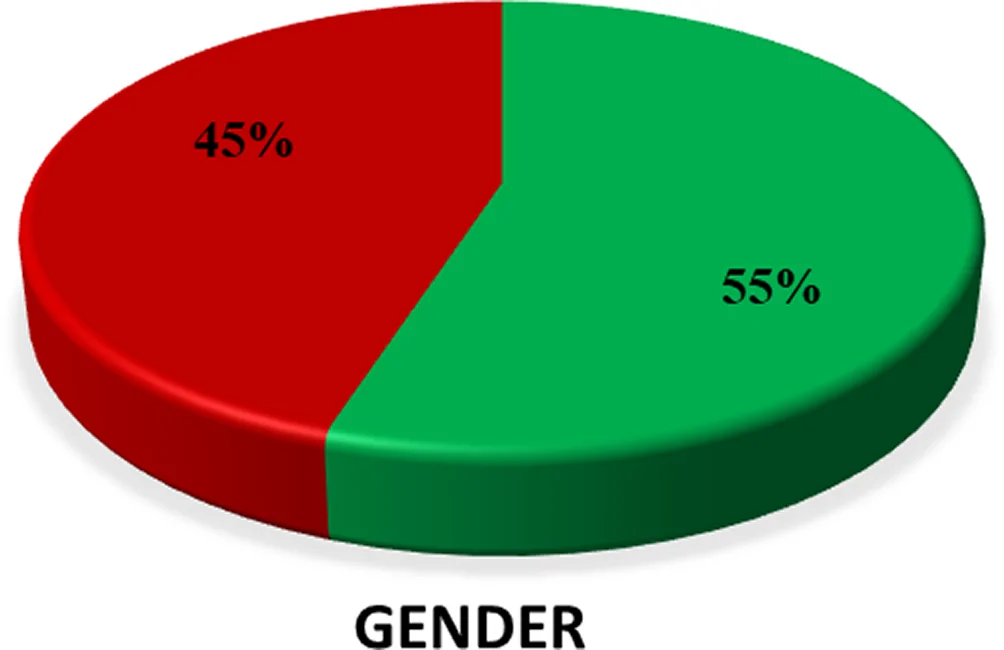
Percentages of identifying information about the gender variable.

**Figure 2.**
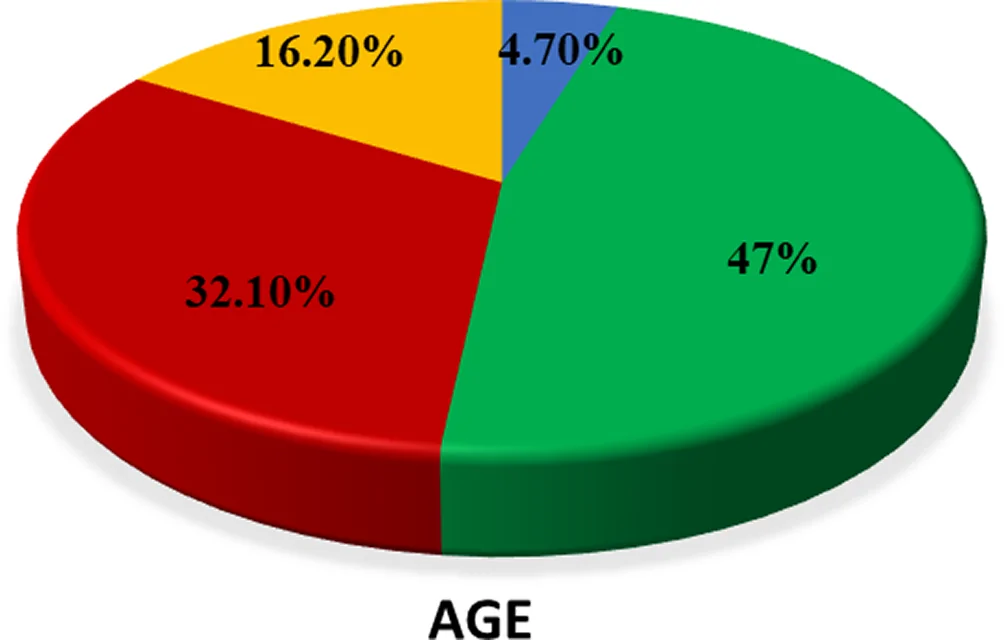
Percentages of identifying information about the age variable.

**Figure 3.**
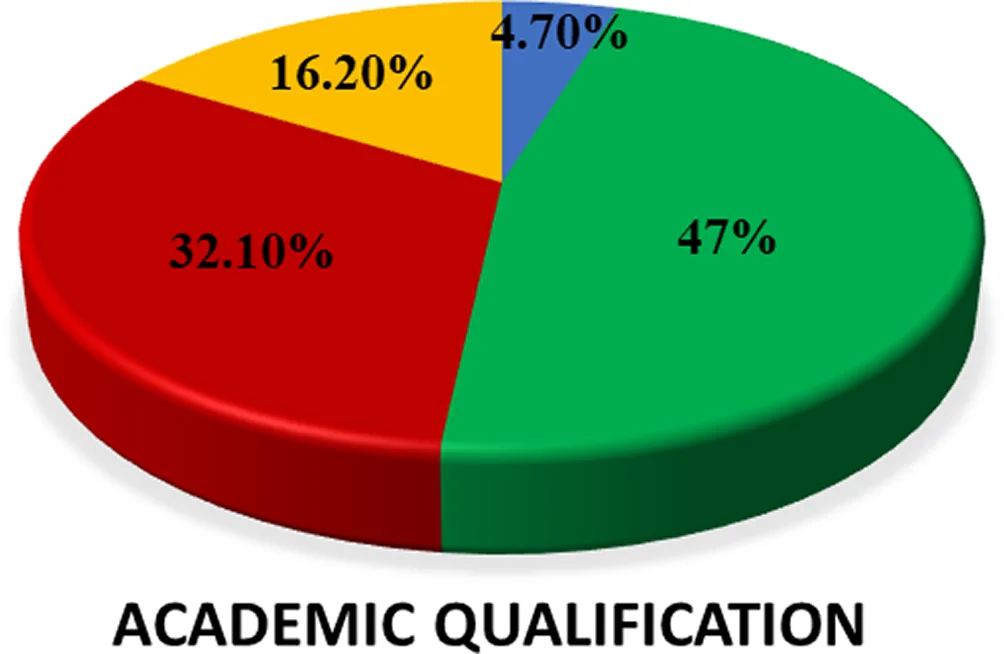
Percentages of identifying information about the Academic qualification variable.

**Table 2.** Levels of importance according to the five-point Likard scale.

Degree of relative importance	RII
*High*	*0.8* ≤ *RII* ≤ *1*
*Medium to high*	*0.6* ≤ *RII < 0.8*
*Medium*	*0.4* ≤ *RII < 0.6*
*Low to medium*	*0.2* ≤ *RII < 0.4*
*Low*	*0* ≤ *RII < 0.2*

1- **Describe and diagnose the independent variable social marketing**
  ➢ **Social product**
The basic statistical measures were found in order to describe and diagnose the paragraphs of the social product dimension, which included paragraphs (X1-X6). Table 3 explains this description. The average dimension was (3.867), while the standard deviation was (0.765) and of relative importance (77.348%), as this percentage is classified as a medium to high percentage as an indicator of relative importance (RII). As for the percentages of (agreement), opinion (agreed to some extent), and (disagreement), their percentages reached (73.93%), (21.57%), and (4.50%), respectively. Note that the highest paragraph is X1 (Fallujah University provides sufficient information about its services that contribute to meeting the social needs of students and subscribers), and the lowest paragraph is X5 (Fallujah University seeks to continuously develop and improve its services to advance the social aspect of students and the surrounding society). Figure 4 explains illustrates the of relative importance of each item in the social product.
  ➢ **Social pricing**
Basic statistical measures were found in order to describe and diagnose paragraphs of the social pricing dimension, which included paragraphs (X7-X12). Table 4 explains this description. The average dimension was (3.760), while the standard deviation was (0.833) and of relative importance (75.194%), as this percentage is classified as a medium to high percentage as an indicator of relative importance (RII). As for the percentages of (agreement), opinion (agreed to some extent), and (disagreement), their percentages reached (70.77%), (20.00%), and (9.23%), respectively. Note that the highest paragraph is X7 (Fallujah University offers attractive facilities and discounts to students and beneficiaries as a type of practice that supports society), and the lowest paragraph is X10 (the prices of services and activities provided by Fallujah University are proportional to the purchasing power of students and beneficiaries). Figure 5 explains illustrates the of relative importance of each item in the social pricing.
  ➢ **Social promotion**
Basic statistical measures were found in order to describe and diagnose paragraphs of the social promotion dimension, which included paragraphs (X13-X18). Table 5 explains this description. The average dimension was (3.890), while the standard deviation was (0.779) and of relative importance (77.798%), as this percentage is classified as a medium to high percentage as an indicator of relative importance (RII). As for the percentages of (agreement), opinion (agreed to some extent), and (disagreement), their percentages reached (74.55%), (19.78%), and (5.67%), respectively. Note that the highest paragraph is X13 (Fallujah University displays advertisements that include sufficient information about the services and offers it provides to students and the community), and the lowest paragraph is X14 (Fallujah University is committed to credibility in advertisements directed to students and beneficiaries, as a way to avoid any marketing deception towards the community). Figure 6 explains illustrates the of relative importance of each item in the social promotion.
  ➢ **Social place**
Basic statistical measures were found in order to describe and diagnose paragraphs of the social space dimension, which included paragraphs (X19-X24). Table 6 explains this description. The average dimension was (3.912), while the standard deviation was (0.796) and of relative importance (78.232%), as this percentage is classified as a medium to high percentage as an indicator of relative importance (RII). As for the percentages of (agreement), opinion (agreed to some extent), and (disagreement), their percentages reached (75.33%), (19.57%), and (5.10%), respectively. Note that the highest paragraph is X19 (Fallujah University provides extensive coverage in all regions and the services it provides, even if this requires additional costs), and the lowest paragraph is X20 (Fallujah University's website is easily accessible by students and beneficiaries). Figure 7 explains illustrates the of relative importance of each item in the social place.
2- **Describe and diagnose the independent variable of digital etiquette**
  ➢ **Technological dimension**
Basic statistical measures were found in order to describe and diagnose paragraphs of the technological dimension, which included paragraphs (y1-y6). Table 7 explains this description. The average dimension was (4.044), while the standard deviation was (0.742) and of relative importance (80.884%), as this percentage is classified as a high percentage as an indicator of relative importance (RII). As for the percentages of (agreement), opinion (agreed to some extent), and (disagreement), their percentages reached (80.07%), (16.42%), and (3.52%), respectively. Note that the highest paragraph is y2 (turn on the camera or microphone during simultaneous meetings for remote communication applications, at the appropriate time), and the lowest paragraph is y6 (speak tactfully when communicating with others in remote communication applications). Figure 8 explains illustrates the of relative importance of each item in the technological dimension.
  ➢ **Moral dimension**
Basic statistical measures were found in order to describe and diagnose paragraphs of the moral dimension, which included paragraphs (X13-X18). Table 8 explains this description. The average dimension was (3.733), while the standard deviation was (0.852) and of relative importance (74.667%), as this percentage is classified as a medium to high percentage as an indicator of relative importance (RII). As for the percentages of (agreement), opinion (agreed to some extent), and (disagreement), their percentages reached (65.97%), (25.02%), and (9.02%), respectively. Note that the highest paragraph is y10 (I do not publish unethical posts that offend others), and the lowest paragraph is y7 (I respect the privacy of electronic platforms and do not mishandle them). Figure 9 explains illustrates the of relative importance of each item in the moral dimension.
  ➢ **Social dimension**
Basic statistical measures were found in order to describe and diagnose paragraphs of the social dimension, which included paragraphs (X13-X18). Table 9 explains this description. The average dimension was (4.018), while the standard deviation was (0.811) and of relative importance (80.357%), as this percentage is classified as a high percentage as an indicator of relative importance (RII). As for the percentages of (agreement), opinion (agreed to some extent), and (disagreement), their percentages reached (81.00%), (14.42%), and (4.58%), respectively. Note that the highest paragraph is y16 (post posters and awareness bulletins on social media), and the lowest paragraph is y13 (commit to addressing individuals in the virtual world in a decent manner). Figure 10 explains illustrates the of relative importance of each item in the social dimension.
  ➢ **Health dimension**
Basic statistical measures were found to describe and diagnose the health dimension items, which included items (X13-X18). Table 10 explains this description. The average dimension was (4.126), while the standard deviation was (0.770) and of relative importance (82.512%), as this percentage is classified as a high percentage as an indicator of relative importance (RII). As for the percentages of (agreement), opinion (agreed to some extent), and (disagreement), their percentages reached (83.53%), (12.87%), and (3.60%), respectively. Note that the highest paragraph is y24 (I isolate myself from others when dealing with cyberspace), and the lowest paragraph is y19 (I am aware of the risks inherent when using digital technology). Figure 11 explains illustrates the of relative importance of each item in the health dimension.
  ➢ **Security dimension**
Basic statistical measures were found in order to describe and diagnose paragraphs of the security dimension, which included paragraphs (X13-X18). Table 11 explains this description. The average dimension was (3.884), while the standard deviation was (0.824) and of relative importance (77.674%), as this percentage is classified as a medium to high percentage as an indicator of relative importance (RII). As for the percentages of (agreement), opinion (agreed to some extent), and (disagreement), their percentages reached (74.95%), (19.23%), and (5.82%), respectively. Note that the highest paragraph is y26 (when dealing with devices and cyberspace, I put passwords that are difficult to predict), and the lowest paragraph is y30 (stay away from using anonymous programs, because they obtain information without the permission of their owner). Figure 12 explains illustrates the of relative importance of each item in the security dimension.

**Table 3.** Frequency distributions, arithmetic means, standard deviations, and the relative importance of the social product dimension.

Paragraphs	Response										Average	Standard deviation	Response intensity %	Ranking
	Totally agree (5)		Agree (4)		Neutral (3)		Idon't agree (2)		Don't totally agree (1)					
	Number	%	Number	%	Number	%	Number	%	Number	%				
**X1**	43	20	129	60	39	18.1	3	1.4	1	0.5	3.977	0.693	79.534	1
**X2**	46	21.4	121	56.3	41	19.1	7	3.2	0	0	3.958	0.732	79.162	2
**X3**	34	15.8	122	56.7	50	23.3	8	3.7	1	0.5	3.837	0.747	76.744	4
**X4**	40	18.6	123	57.2	44	20.5	7	3.2	1	0.5	3.902	0.746	78.046	3
**X5**	31	14.3	116	54	52	24.2	12	5.6	4	1.9	3.735	0.842	74.698	6
**X6**	38	17.7	111	51.6	52	24.2	12	5.6	2	0.9	3.80	0.83	75.906	5
**Overall rate**		17.97		55.97		21.57		3.78		0.72	3.867	0.765	77.348	
**Total**	73.93				21.57		4.50							

**Figure 4.**
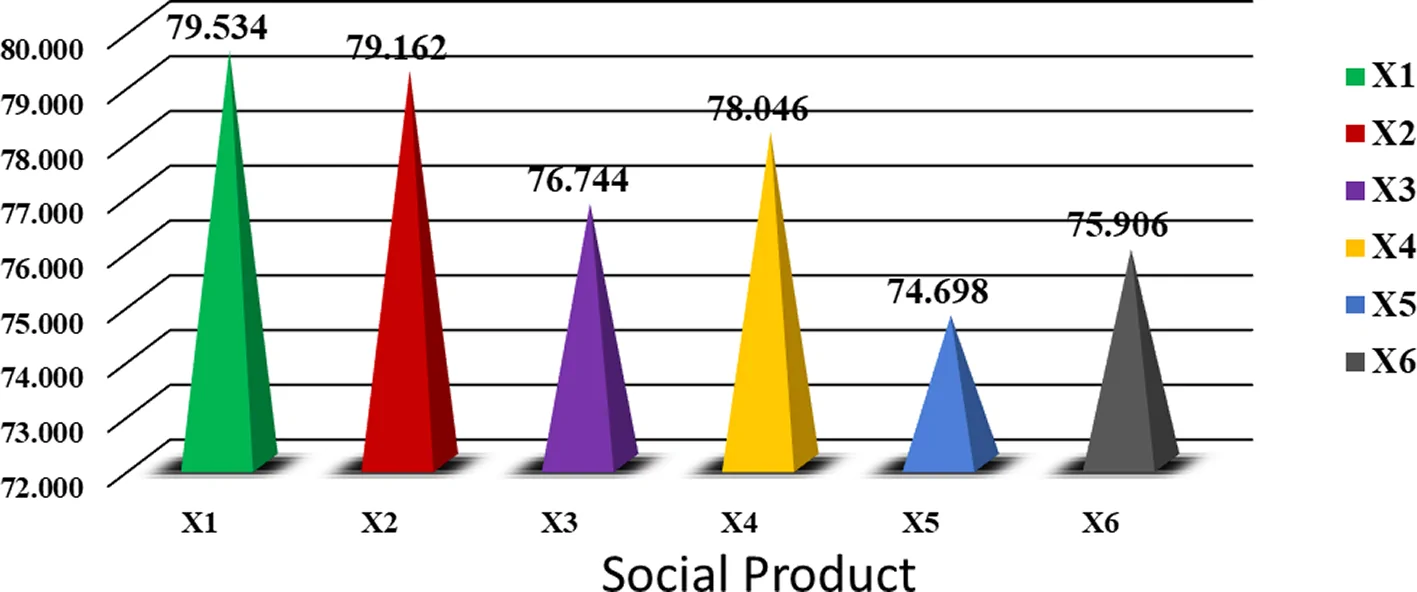
Explains illustrates the relative importance of each item in the social product.

**Table 4.** Description of the social pricing dimension and its results.

Paragraphs	Response										Average	Standard deviation	Response intensity %	Ranking
	Totally agree (5)		Agree (4)		Neutral (3)		Idon't agree (2)		Don't totally agree (1)					
	Number	%	Number	%	Number	%	Number	%	Number	%				
**X7**	43	20	116	54	35	16.2	18	8.4	3	1.4	3.828	0.893	76.558	1
**X8**	27	12.6	108	50.2	52	24.2	23	10.7	5	2.3	3.600	0.921	72.000	5
**X9**	32	14.9	132	61.3	41	19.1	10	4.7	0	0	3.865	0.714	77.302	4
**X10**	23	10.7	110	51.1	50	23.3	31	14.4	1	0.5	3.572	0.882	71.442	6
**X11**	39	18.1	117	54.5	44	20.5	13	6	2	0.9	3.828	0.828	76.558	2
**X12**	34	15.8	132	61.4	36	16.7	12	5.6	1	0.5	3.87	0.760	77.302	3
**Overall rate**		15.35		55.42		20.00		8.30		0.93	3.760	0.833	75.194	
**Total**	70.77				20.00		9.23							

**Figure 5.**
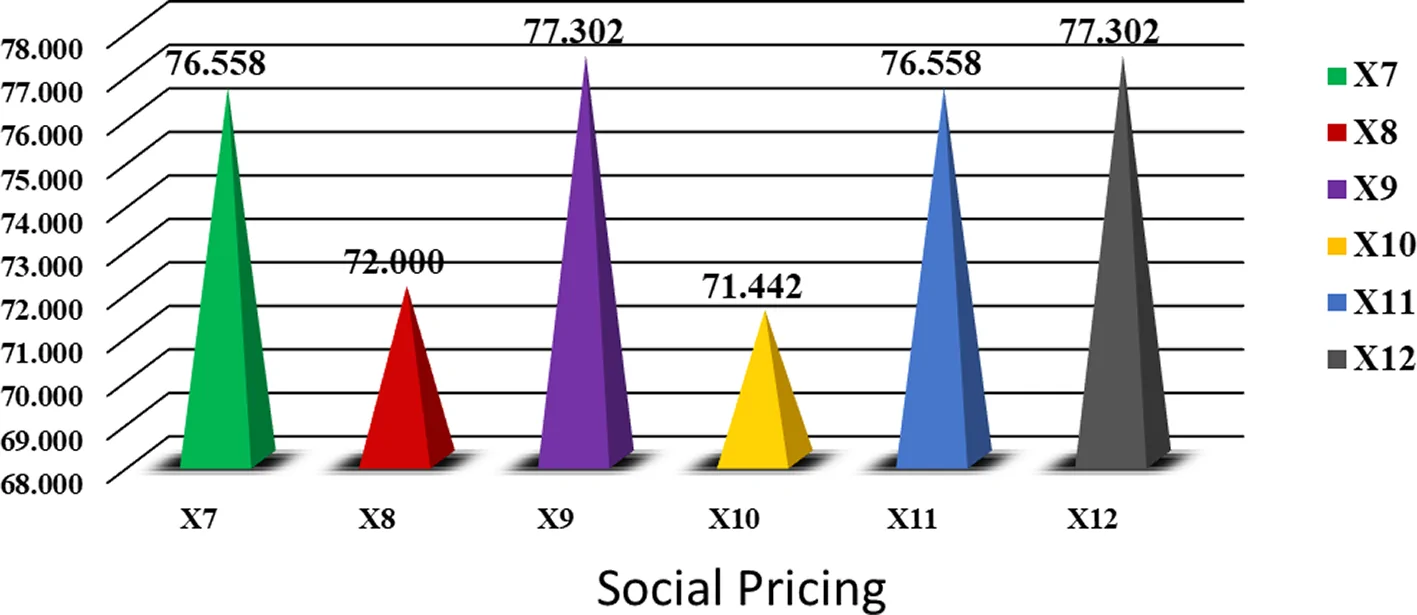
Explains illustrates the relative importance of each item in the social pricing.

**Table 5.** Frequency distributions, arithmetic averages, standard deviations, and the relative importance of the social promotion dimension.

Paragraphs	Response										Average	Standard deviation	Response intensity %	Ranking
	Totally agree (5)		Agree (4)		Neutral (3)		Idon't agree (2)		Don't totally agree (1)					
	Number	%	Number	%	Number	%	Number	%	Number	%				
**X13**	58	27	122	56.7	26	12.1	8	3.7	1	0.5	4.061	0.762	81.210	1
**X14**	28	13	98	45.6	67	31.2	19	8.8	3	1.4	3.600	0.874	72.000	6
**X15**	55	25.6	115	53.5	36	16.7	6	2.8	3	1.4	3.991	0.815	79.814	2
**X16**	51	23.7	113	52.5	41	19.1	9	4.2	1	0.5	3.949	0.798	78.976	4
**X17**	37	17.2	117	54.4	47	21.9	13	6	1	0.5	3.819	0.803	76.372	5
**X18**	40	18.6	128	59.5	38	17.7	8	3.7	1	0.5	3.92	0.74	78.418	3
**Overall rate**		20.85		53.70		19.78		4.87		0.80	3.890	0.799	77.798	
**Total**	74.55				19.78		5.67							

**Figure 6.**
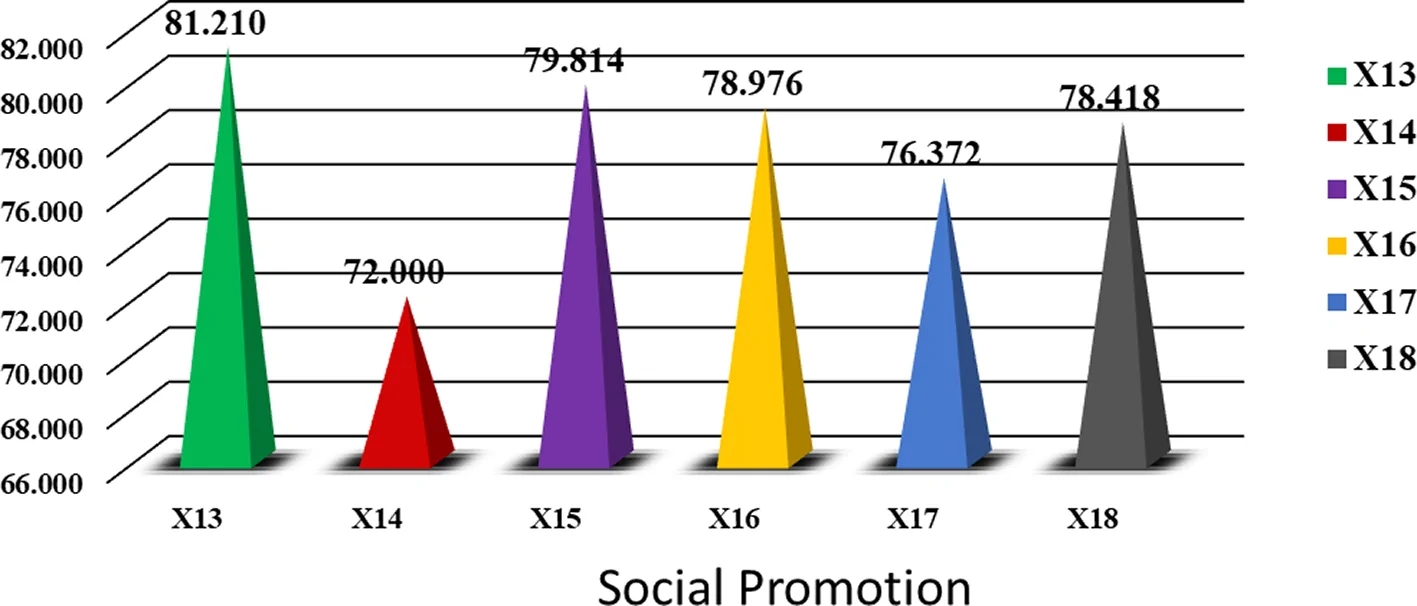
Explains illustrates the relative importance of each item in the social promotion.

**Table 6.** Frequency distributions, arithmetic means, standard deviations, and the relative importance of the social space dimension.

Paragraphs	Response										Average	Standard deviation	Response intensity %	Ranking
	Totally agree (5)		Agree (4)		Neutral (3)		Idon't agree (2)		Don't totally agree (1)					
	Number	%	Number	%	Number	%	Number	%	Number	%				
**X19**	60	27.9	121	56.3	29	13.5	3	1.4	2	0.9	4.088	0.740	81.768	1
**X20**	38	17.7	102	47.4	57	26.5	15	7	3	1.4	3.730	0.882	74.604	6
**X21**	29	13.5	117	54.4	61	28.4	6	2.8	2	0.9	3.767	0.750	75.348	5
**X22**	58	27	114	53	30	14	11	5.1	2	0.9	4.000	0.837	80.000	3
**X23**	42	19.5	118	54.9	39	18.2	14	6.5	2	0.9	3.856	0.839	77.116	4
**X24**	54	25.1	119	55.3	36	16.8	6	2.8	0	0	4.03	0.73	80.558	2
**Overall rate**		21.78		53.55		19.57		4.27		0.83	3.912	0.796	78.232	
**Total**	75.33				19.57		5.10							

**Figure 7.**
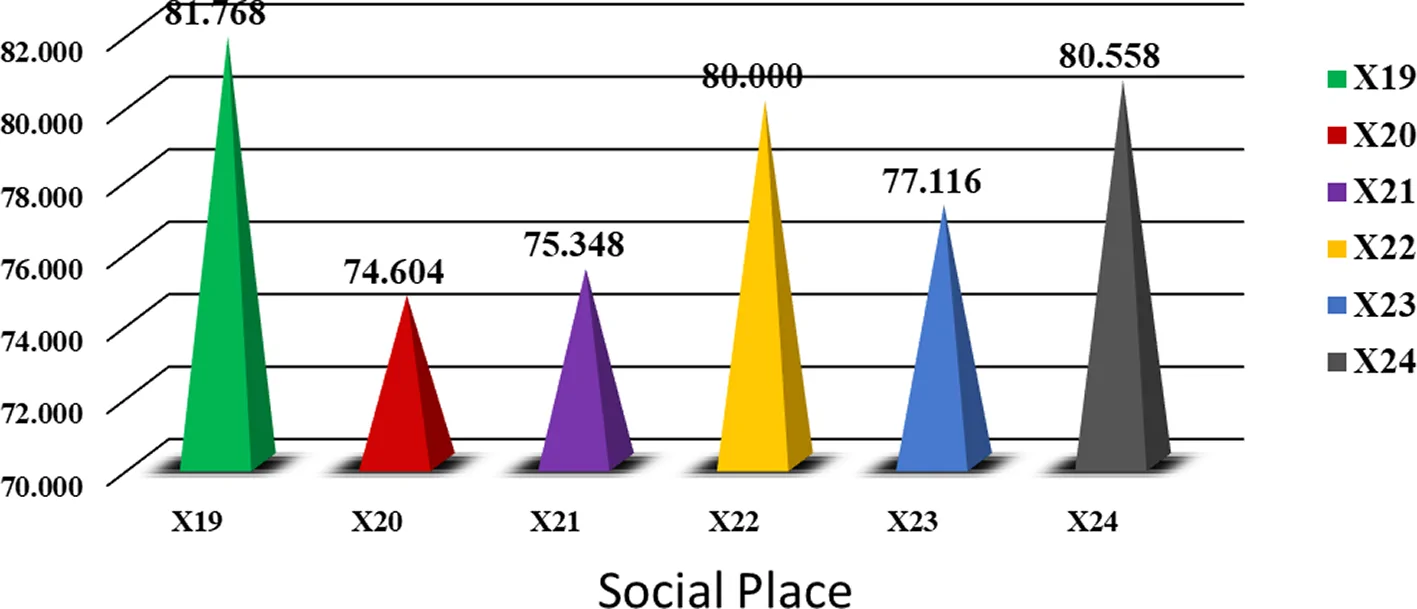
Explains illustrates the relative importance of each item in the social place.

**Table 7.** Frequency distributions, arithmetic means, standard deviations, and the relative importance of the technological dimension.

Paragraphs	Response										Average	Standard deviation	Response intensity %	Ranking
	Totally agree (5)		Agree (4)		Neutral (3)		Idon't agree (2)		Don't totally agree (1)					
	Number	%	Number	%	Number	%	Number	%	Number	%				
**y1**	58	27	128	59.5	26	12.1	3	1.4	0	0	4.121	0.659	82.418	3
**y2**	83	38.6	109	50.7	21	9.7	1	0.5	1	0.5	4.265	0.690	85.302	1
**y3**	82	38.1	110	51.2	21	9.8	2	0.9	0	0	4.265	0.669	85.302	2
**y4**	59	27.4	109	50.7	42	19.5	4	1.9	1	0.5	4.028	0.767	80.558	4
**y5**	51	23.7	123	57.2	33	15.3	7	3.3	1	0.5	4.005	0.752	80.094	5
**y6**	32	14.9	89	41.4	69	32.1	22	10.2	3	1.4	3.58	0.91	71.628	6
**Overall rate**		28.28		51.78		16.42		3.03		0.48	4.044	0.742	80.884	
**Total**	80.07				16.42		3.52							

**Figure 8.**
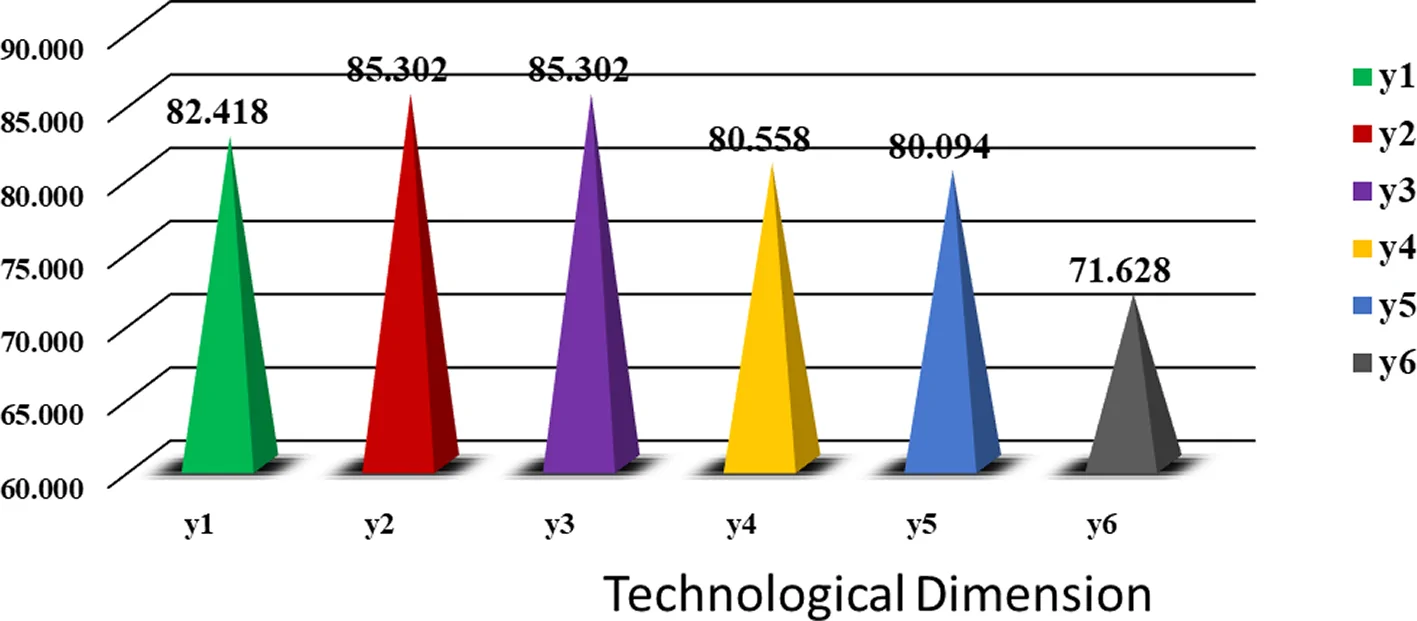
Explains illustrates the relative importance of each item in the technological dimension.

**Table 8.** Frequency distributions, arithmetic averages, standard deviations, and relative importance of the ethical dimension.

Paragraphs	Response										Average	Standard deviation	Response intensity %	Ranking
	Totally agree (5)		Agree (4)		Neutral (3)		Idon't agree (2)		Don't totally agree (1)					
	Number	%	Number	%	Number	%	Number	%	Number	%				
**y7**	24	11.2	86	40	77	35.7	24	11.2	4	1.9	3.474	0.901	69.488	6
**y8**	28	13	95	44.2	64	29.8	26	12.1	2	0.9	3.563	0.899	71.256	4
**y9**	26	12.1	90	41.9	67	31.1	26	12.1	6	2.8	3.484	0.951	69.674	5
**y10**	63	29.3	114	53	29	13.5	7	3.3	2	0.9	4.065	0.800	81.302	1
**y11**	47	21.8	116	54	43	20	7	3.3	2	0.9	3.926	0.794	78.512	2
**y12**	40	18.6	122	56.7	43	20	9	4.2	1	0.5	3.89	0.77	77.768	3
**Overall rate**		17.67		48.30		25.02		7.70		1.32	3.733	0.852	74.667	
**Total**	65.97				25.02		9.02							

**Figure 9.**
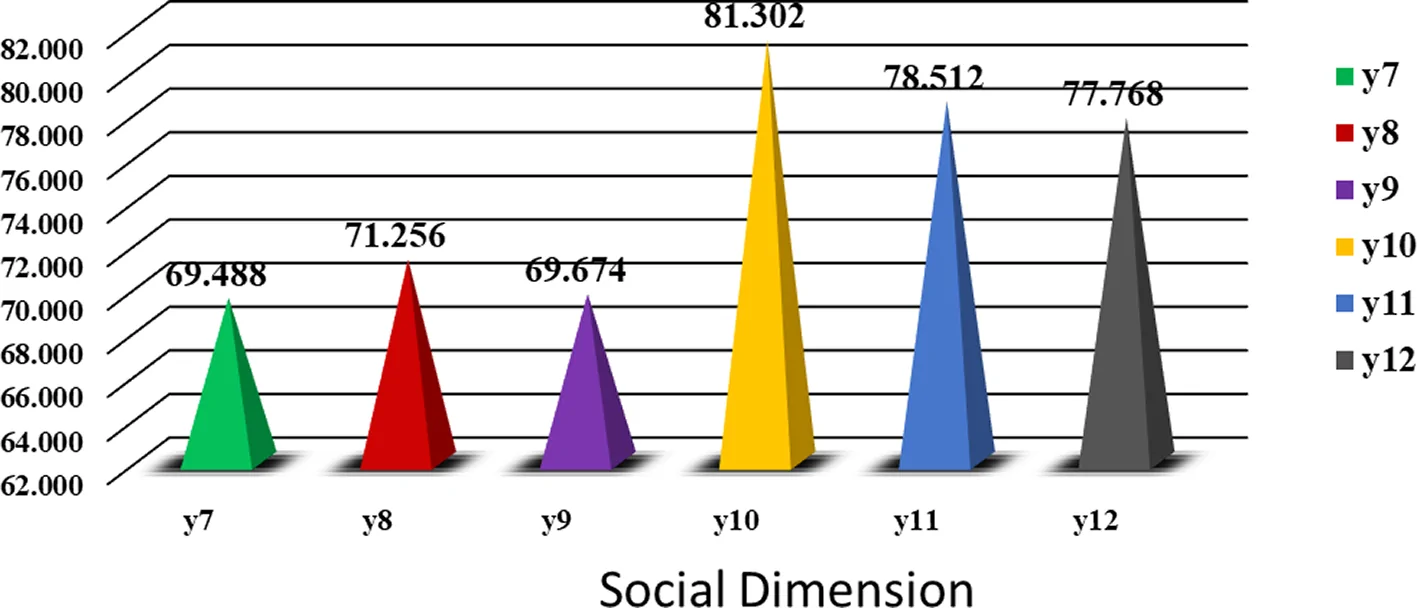
Explains illustrates the relative importance of each item in the moral dimension.

**Table 9.** Frequency distributions, arithmetic averages, standard deviations, and relative importance of the social dimension.

Paragraphs	Response										Average	Standard deviation	Response intensity %	Ranking
	Totally agree (5)		Agree (4)		Neutral (3)		Idon't agree (2)		Don't totally agree (1)					
	Number	%	Number	%	Number	%	Number	%	Number	%				
**y13**	51	23.7	109	50.7	39	18.2	11	5.1	5	2.3	3.884	0.907	77.674	6
**y14**	68	31.6	110	51.2	25	11.6	8	3.7	4	1.9	4.070	0.865	81.396	3
**y15**	63	29.3	120	55.8	23	10.7	4	1.9	5	2.3	4.079	0.825	81.582	2
**y16**	69	32.1	121	56.3	20	9.3	3	1.4	2	0.9	4.172	0.725	83.442	1
**y17**	47	21.9	120	55.8	39	18.1	7	3.3	2	0.9	3.944	0.783	78.884	5
**y18**	48	22.3	119	55.3	40	18.6	7	3.3	1	0.5	3.96	0.76	79.162	4
**Overall rate**		26.82		54.18		14.42		3.12		1.47	4.018	0.811	80.357	
**Total**	81.00				14.42		4.58							

**Figure 10.**
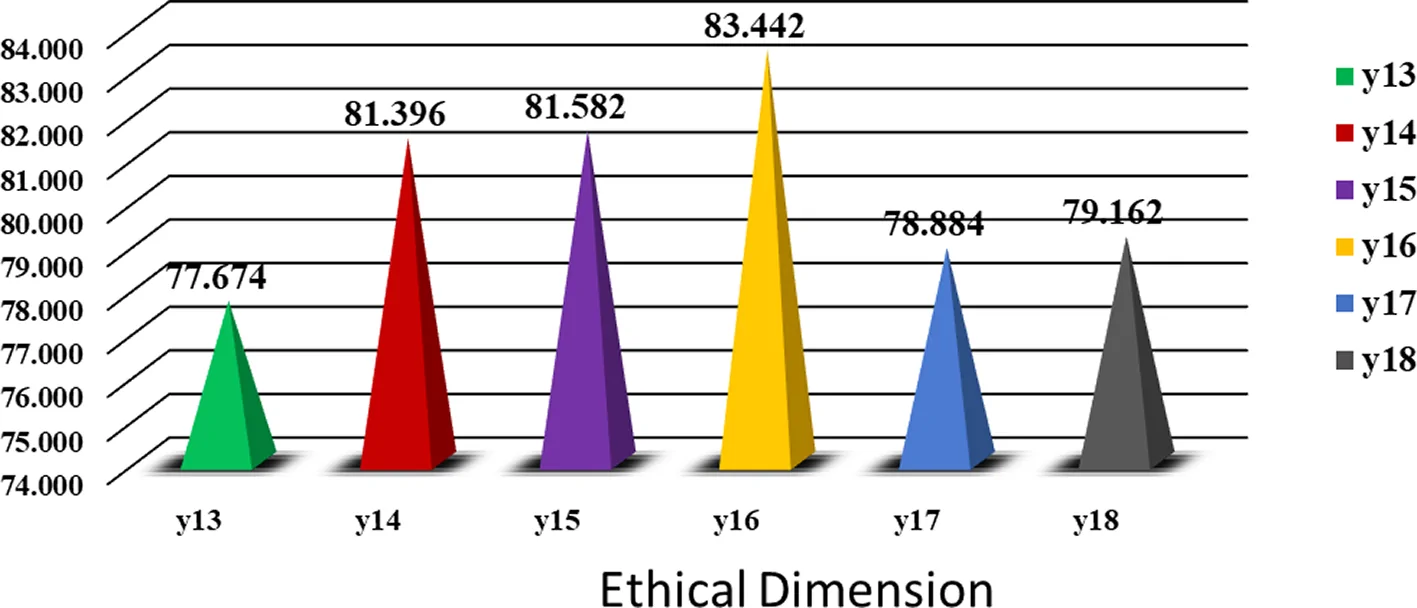
Explains illustrates the relative importance of each item in the social dimension.

**Table 10.** Frequency distributions, arithmetic means, standard deviations, and relative importance of the health dimension.

Paragraphs	Response										Average	Standard deviation	Response intensity %	Ranking
	Totally agree (5)		Agree (4)		Neutral (3)		Idon't agree (2)		Don't totally agree (1)					
	Number	%	Number	%	Number	%	Number	%	Number	%				
**y19**	46	21.4	119	55.3	37	17.2	10	4.7	3	1.4	3.907	0.832	78.140	6
**y20**	68	31.6	110	51.2	29	13.4	7	3.3	1	0.5	4.102	0.785	82.046	4
**y21**	57	26.4	127	59.1	26	12.1	4	1.9	1	0.5	4.093	0.704	81.860	5
**y22**	74	34.4	113	52.6	23	10.7	5	2.3	0	0	4.191	0.714	83.814	3
**y23**	89	41.3	89	41.4	30	14	7	3.3	0	0	4.209	0.802	84.186	2
**y24**	91	42.3	95	44.2	21	9.8	8	3.7	0	0	4.25	0.78	85.024	1
**Overall rate**		32.90		50.63		12.87		3.20		0.40	4.126	0.770	82.512	
**Total**	83.53				12.87		3.60							

**Figure 11.**
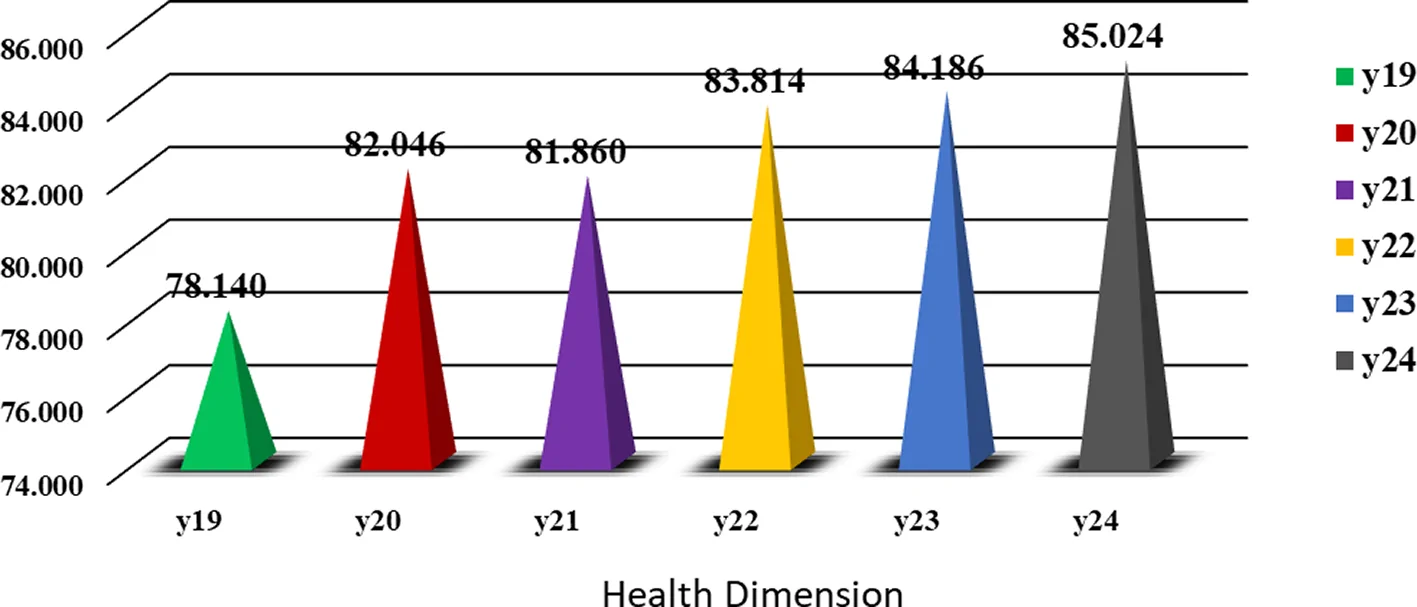
Explains illustrates the relative importance of each item in the health dimension.

**Table 11.** Frequency distributions, arithmetic averages, standard deviations, and the relative importance of the security dimension.

Paragraphs	Response										Average	Standard deviation	Response intensity %	Ranking
	Totally agree (5)		Agree (4)		Neutral (3)		Idon't agree (2)		Don't totally agree (1)					
	Number	%	Number	%	Number	%	Number	%	Number	%				
**y25**	59	27.4	117	54.4	30	14	6	2.8	3	1.4	4.037	0.808	80.744	2
**y26**	58	27	122	56.7	32	14.9	2	0.9	1	0.5	4.088	0.702	81.768	1
**y27**	42	19.5	118	54.9	46	21.4	6	2.8	3	1.4	3.884	0.797	77.674	3
**y28**	43	20	111	51.6	52	24.2	5	2.3	4	1.9	3.856	0.827	77.116	4
**y29**	38	17.7	118	54.9	43	20	14	6.5	2	0.9	3.819	0.831	76.372	5
**y30**	30	14	111	51.6	45	20.9	20	9.3	9	4.2	3.62	0.98	72.372	6
**Overall rate**		20.93		54.02		19.23		4.10		1.72	3.884	0.824	77.674	
**Total**	74.95				19.23		5.82							

**Figure 12.**
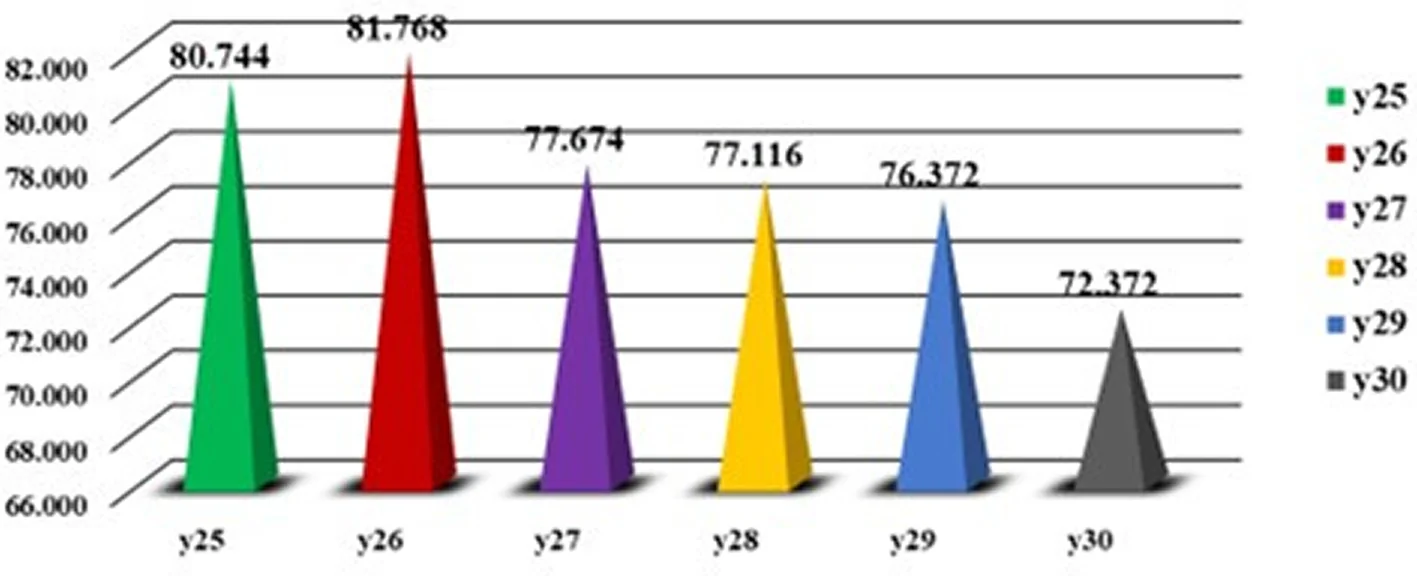
Relative importance of the security dimension.

### Relative importance

From Table 12 above, it is noted that the digital etiquette variable received the highest comparative relative importance, which amounted to (79,200%), while the social marketing variable received relative importance amounting to (77,140%) for the sample studied.

**Table 12.** The relative importance of the study variables.

*T*	*Dimensions*	*Arithmetic mean*	*Standard deviation*	*Relative importance%*	*Ranking*
***1***	***Social marketing***	***3.857***	***0.436***	***77.140***	***2***
***2***	***Digital etiquette***	***3.960***	***0.455***	***79.200***	***1***

### Fifth: Measuring the stability of the questionnaire (Reliability test)

( Feldt & Brennan, 1989) classified the values of the reliability test into two levels. If the values are greater than (70%), they are considered high-level values, while the values that are less than (70%) are considered low-level values.

The stratified alpha coefficient was found as shown in Table 13 below:

**Table 13.** Measuring the stability of the study variables.

*key variables*	*Alpha stratified coefficient for combined dimensions*
***Alpha stratified coefficient for combined dimensions*** α _st._	***0.94***

It is noted that the value of the stratified alpha coefficient reached 0.94, which is greater than 0.70% for the study variables. Thus, it can be said, through the stratified alpha coefficient, that there is a stability force for the study variables.

### Internal consistency of study dimensions

From Table 14, it is noted that the correlation coefficient values for the social marketing variable appeared between (0.576-0.721), which is greater than (0.3) ( Wu, M. et al. (2016)). It is also noted that the correlation coefficient values for the digital etiquette variable appeared between (0.504-0.813), which is also greater than (0.3), which indicates that there is a moral correlation between the paragraphs and the dimensions they represent.

**Table 14.** Internal consistency of variable dimensions.

Internal consistency							
Variables	sub-dimensions	Paragraph sequence					
		First	Second	Third	Fourth	Fifth	Sixth
		The degree to which the paragraph relates to the dimension					
		(P-value)					
**Social marketing**	**Social product**	0.658	0.665	0.694	0.66	0.606	0.688
		0.000	0.000	0.000	0.000	0.000	0.000
	**Social pricing**	0.656	0.695	0.576	0.701	0.674	0.649
		0.000	0.000	0.000	0.000	0.000	0.000
	**Social promotion**	0.612	0.656	0.703	0.721	0.671	0.651
		0.000	0.000	0.000	0.000	0.000	0.000
	**Social place**	0.599	0.649	0.686	0.59	0.65	0.652
		0.000	0.000	0.000	0.000	0.000	0.000
**Digital etiquette**	**Technological dimension**	0.685	0.644	0.666	0.772	0.746	0.504
		0.000	0.000	0.000	0.000	0.000	0.000
	**Ethical dimension**	0.787	0.79	0.803	0.572	0.66	0.659
		0.000	0.000	0.000	0.000	0.000	0.000
	**Social dimension**	0.678	0.711	0.691	0.615	0.654	0.654
		0.000	0.000	0.000	0.000	0.000	0.000
	**Health dimension**	0.702	0.685	0.646	0.793	0.79	0.801
		0.000	0.000	0.000	0.000	0.000	0.000
	**Security dimension**	0.656	0.677	0.77	0.813	0.8	0.729
		0.000	0.000	0.000	0.000	0.000	0.000

### Common Method Bias (CMB)

One of the most important sources for testing the bias of the common method is (the lack of diversification of the sources from which data is collected, the answer is yes regardless of the question, the questions on the form are characterized by their large number, which in turn leads to the length of the questionnaire form). The (Harman single factor test) test was used in the common method bias test, which indicated ( Bagozzi & Yi, 1991) that if the value of this test is greater than (50%), this is evidence of a bias in the common method, and the value of this test was found according to SPSS software. The presence of the bias problem leads to the absence of strong correlations between variables and thus does not reflect the relationship between variables, but rather may have other reasons, which leads to obtaining misleading results.

The test value of the studied data (%CMB = 25.453), which is less than (50%), thus shows that the data does not suffer from common method bias.

### Natural distribution test

When conducting hypothesis tests, it requires the verification of several hypotheses. The most important of these is to impose the return of variables to the normal probability distribution. The normal distribution test is one of the tests on which determining estimation methods and testing hypotheses are based. Failure to achieve the assumption of a normal probability distribution is a violation. In this case, traditional methods cannot be used as the most feasible method in the process of estimating and testing hypotheses. Rather, the researcher must use alternative methods that do not require the availability of this assumption. A test (Kolmogorov Smirnov) was conducted to verify this hypothesis.

From the results shown in Table 15, it is noted that all dimensions had a P value of less than 0.05, which means that these dimensions do not return to the normal probability distribution. It can also be noted that the p value of the two variables (social marketing, digital etiquette) appeared less than 0.05, which indicates that these variables do not return to the normal probability distribution either.

**Table 15.** Testing the normal distribution of study variables and dimensions.

Kolmogorov-Smirnov				
Type	Description	Statistic	N	P-value
**Dimensions**	**Social product**	**0.138**	**215**	**0.000**
	**Social pricing**	**0.167**	**215**	**0.000**
	**Social promotion**	**0.121**	**215**	**0.000**
	**Social place**	**0.132**	**215**	**0.000**
	**Technological dimension**	**0.136**	**215**	**0.000**
	**Ethical dimension**	**0.108**	**215**	**0.000**
	**Social dimension**	**0.132**	**215**	**0.000**
	**Health dimension**	**0.129**	**215**	**0.000**
	**Security dimension**	**0.129**	**215**	**0.000**
**Variables**	**Social marketing**	**0.079**	**215**	**0.002**
	**Digital etiquette**	**0.075**	**215**	**0.005**

Figures 13 & 14 explains the non-return of the two main variables (social marketing and digital etiquette).

**Figure 13.**
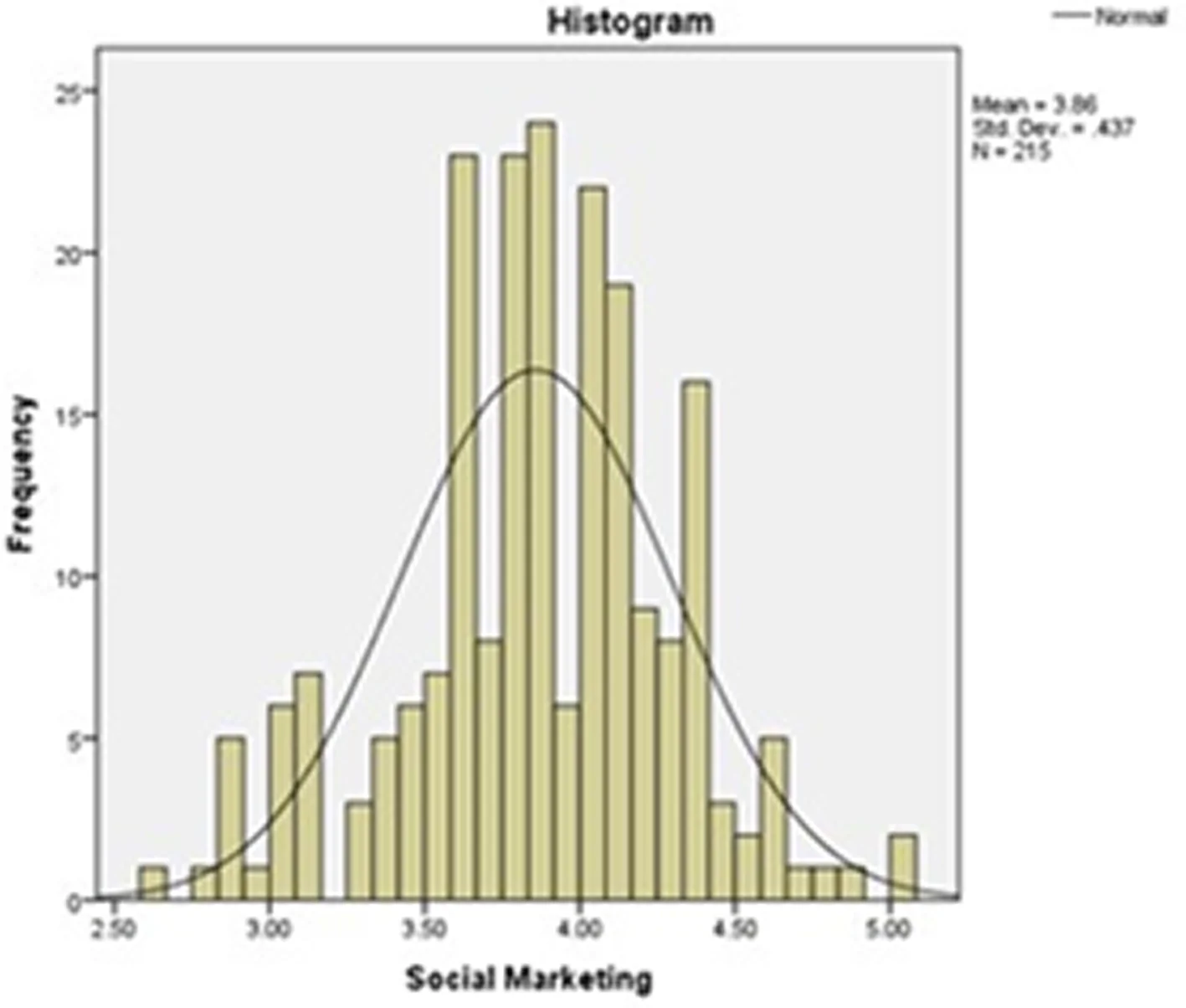
Distribution of the social marketing variable.

**Figure 14.**
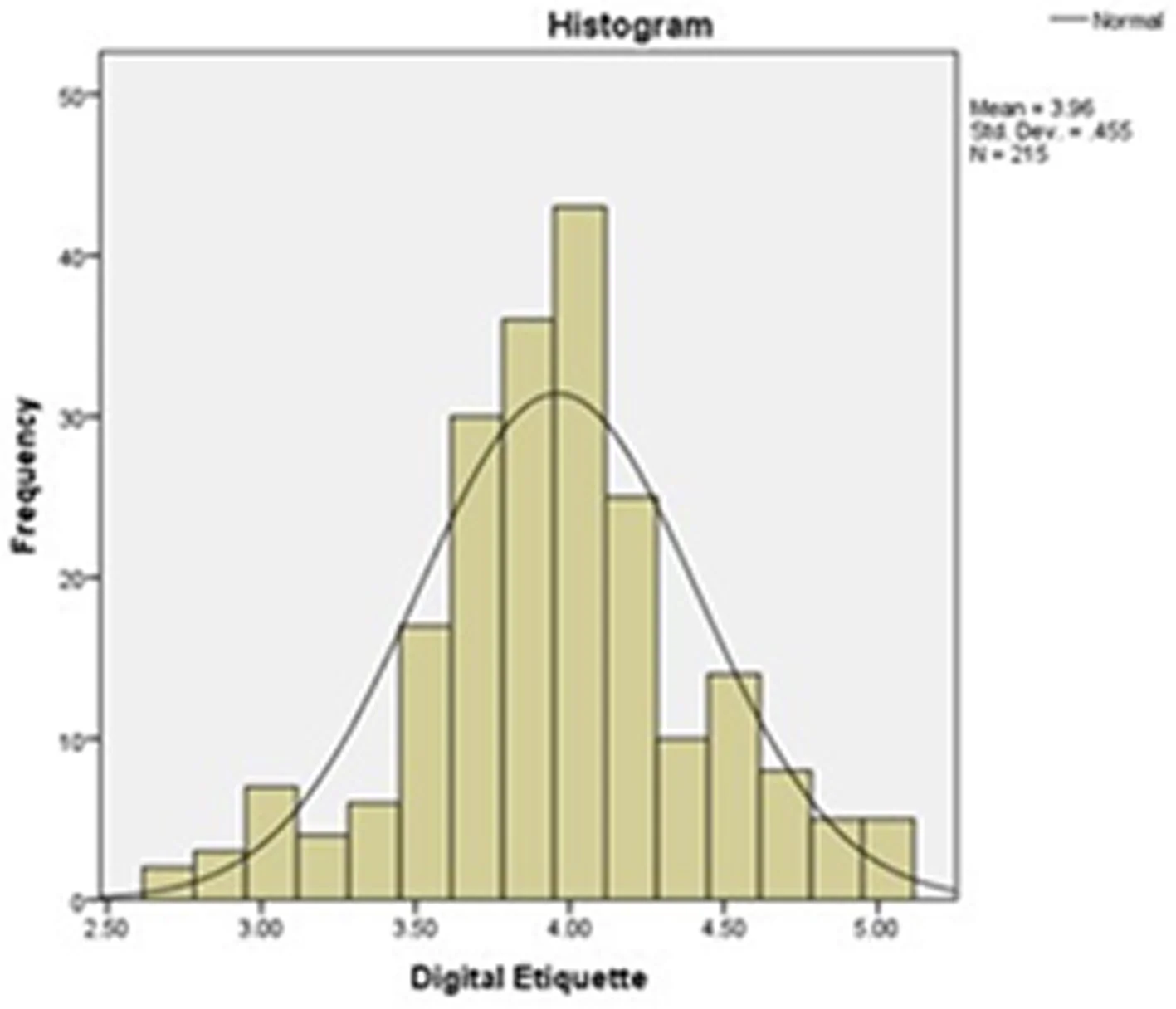
Distribution of the digital etiquette variable.

### Confirmatory factor analysis

The aim of conducting a confirmatory factor analysis of the study variables is to know the correlation relationships between the observations (questions) and the dimension they represent, in addition to knowing the correlation relationships between the latent variables (dimensions) of the variables studied, and thus judging the suitability of the model placed by the researcher with the sample model.

Figure 15 shows the saturation values (Factor Loading) (correlation) of the observed variables (questions) with the indicative (latent) variables related to the study variables, the values of which are shown on the single-headed arrow between the question and the latent variable, as well as the values of the correlation coefficients between each pair of latent variables, the values of which are shown on the double-headed arrow.

**Figure 15.**
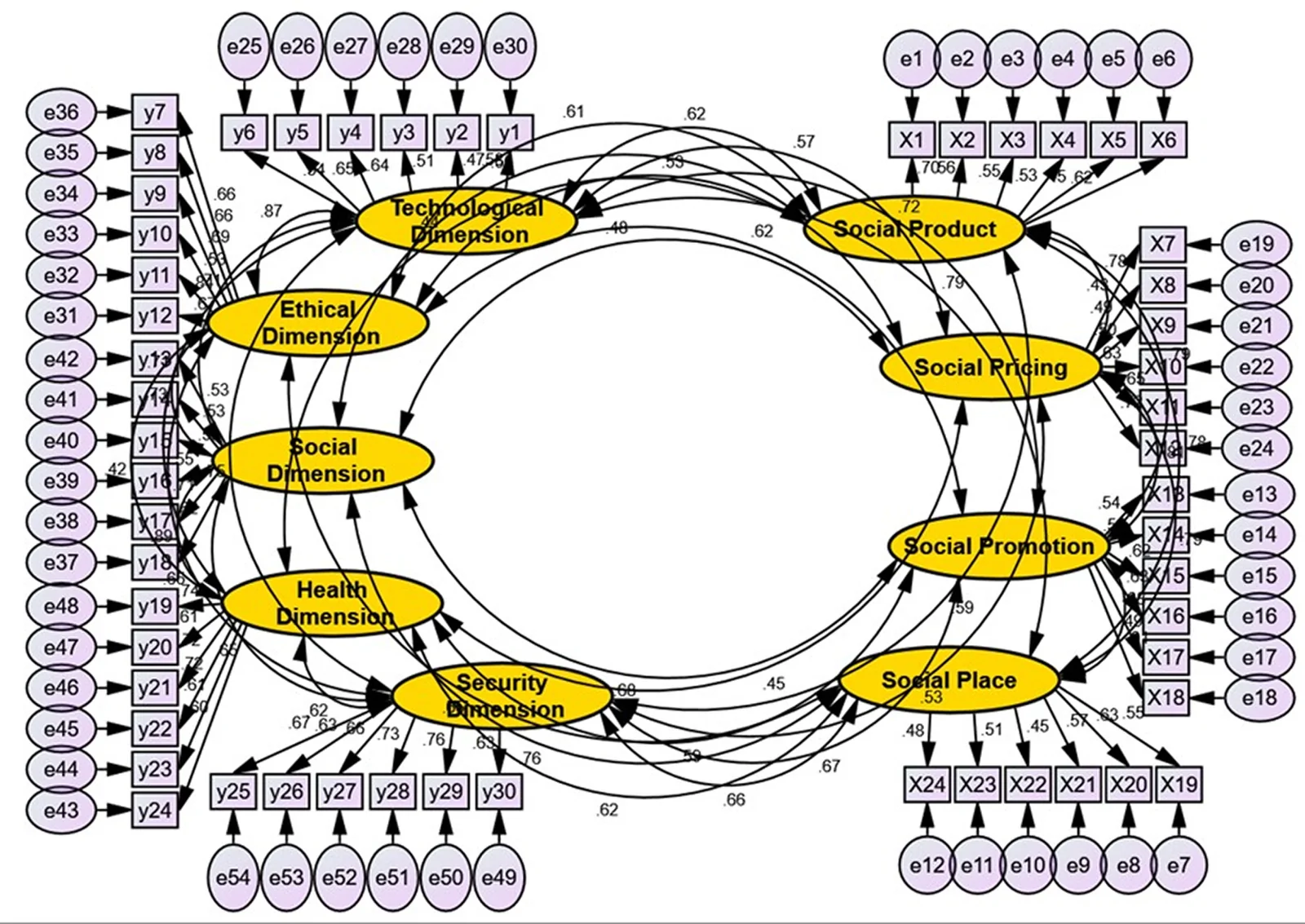
Confirmatory factor analysis of the study variables.

The hypothetical diagram shown in the methodology section determines the significance of the model proposed by the researcher and its conformity with the study sample model. Where conformity quality indicators are verified, which are based on the acceptance limits used by most researchers ( McDonald RP, & MHR, 2002). Through matching quality indicators, it is noted that all indicators are within their acceptable limits, as shown in Table 16 below:

**Table 16.** Conformity quality indicators for confirmatory factor analysis.

Indicators	Value	Result
CMIN/DF	0.60	Identical
GFI	0.93	Identical
AGFI	0.92	Identical
PGFI	0.84	Identical
NFI	0.91	Identical
RFI	0.91	Identical
RMR	0.050	Identical

Standard regression coefficient values

( Hair, et al. 2016) established saturation values determined by sample size. Through Table 17, which shows the values of the standard regression coefficients (SRW-saturation) in addition to the probability values (P-value) accompanying them. All paragraphs appeared to be significant because the probability values (P-value) accompanying them were less than (0.05). As for the saturation values (SRW), they all appeared greater than (0.40).

**Table 17.** Standard regression coefficient values for confirmatory factor analysis results.

Parameter			SRW	P-value	Parameter			SRW	P-value
**X1**	**←**	**Social product**	0.695	0.001	**y1**	**←**	**Technological dimension**	0.579	0.002
**X2**	**←**		0.558	0.002	**y2**	**←**		0.471	0.002
**X3**	**←**		0.55	0.002	**y3**	**←**		0.506	0.001
**X4**	**←**		0.532	0.002	**y4**	**←**		0.636	0.003
**X5**	**←**		0.454	0.002	**y5**	**←**		0.646	0.002
**X6**	**←**		0.624	0.001	**y6**	**←**		0.537	0.003
**X7**	**←**	**Social pricing**	0.428	0.003	**y7**	**←**	**Ethical dimension**	0.663	0.002
**X8**	**←**		0.49	0.002	**y8**	**←**		0.662	0.003
**X9**	**←**		0.495	0.002	**y9**	**←**		0.685	0.001
**X10**	**←**		0.627	0.003	**y10**	**←**		0.532	0.002
**X11**	**←**		0.653	0.002	**y11**	**←**		0.714	0.001
**X12**	**←**		0.716	0.002	**y12**	**←**		0.665	0.003
**X13**	**←**	**Social promotion**	0.54	0.001	**y13**	**←**	**Social dimension**	0.531	0.003
**X14**	**←**		0.518	0.003	**y14**	**←**		0.533	0.002
**X15**	**←**		0.624	0.004	**y15**	**←**		0.528	0.002
**X16**	**←**		0.625	0.003	**y16**	**←**		0.548	0.002
**X17**	**←**		0.675	0.003	**y17**	**←**		0.711	0.002
**X18**	**←**		0.486	0.004	**y18**	**←**		0.622	0.003
**X19**	**←**	**Social place**	0.549	0.002	**y19**	**←**	**Health dimension**	0.736	0.002
**X20**	**←**		0.627	0.002	**y20**	**←**		0.607	0.001
**X21**	**←**		0.57	0.005	**y21**	**←**		0.725	0.002
**X22**	**←**		0.45	0.003	**y22**	**←**		0.724	0.002
**X23**	**←**		0.508	0.002	**y23**	**←**		0.606	0.004
**X24**	**←**		0.482	0.003	**y24**	**←**		0.603	0.003
					**y25**	**←**	**Security dimension**	0.672	0.002
					**y26**	**←**		0.631	0.002
					**y27**	**←**		0.662	0.004
					**y28**	**←**		0.727	0.002
					**y29**	**←**		0.755	0.004
					**y30**	**←**		0.633	0.003

## Testing study hypotheses

### Analysis of impact hypotheses

Testing impact hypotheses is equivalent to testing correlation hypotheses in terms of importance, so impact hypotheses will be analyzed through regression analysis. Regression analysis determines the amount and direction of the effect of an independent variable on another dependent variable

**Second main hypothesis: There is a statistically significant moral impact of social marketing practices on digital etiquette**. Table 18 below shows the results of testing the fourth main hypothesis:

**Table 18.** Impact results of the social marketing variable in digital etiquette.

Independent variable	Direction of impact	Dependent variable	Regression coefficient Estimate(β)	Standard error of the regression coefficient Se.(β)	95% Confidence Interval		P-value	Result
**Social marketing**	**→**	**Digital etiquette**	1.081	0.317	0.695	1.947	0.002	Acceptance

It is noted that the effect of the social marketing variable on the digital etiquette variable is a direct effect through the positive sign of the regression coefficient Estimate (β), which is due to the social marketing variable, which has a value of (1.081), and the standard error value of the regression coefficient is (0.317). Also, both the lower (Lower) and upper (Upper) confidence limits (95%Confidence Interval), which were worth (1.947, 0.695), were similar in signals at a significant level (0.05). It is also noted that this effect is a morally significant effect through the P value of (0.002), which is less than (0.05), which means that there is a moral effect of the social marketing variable on the digital etiquette variable.

Figure 16 explains the effect of the social marketing variable on the digital etiquette variable.

**Figure 16.**
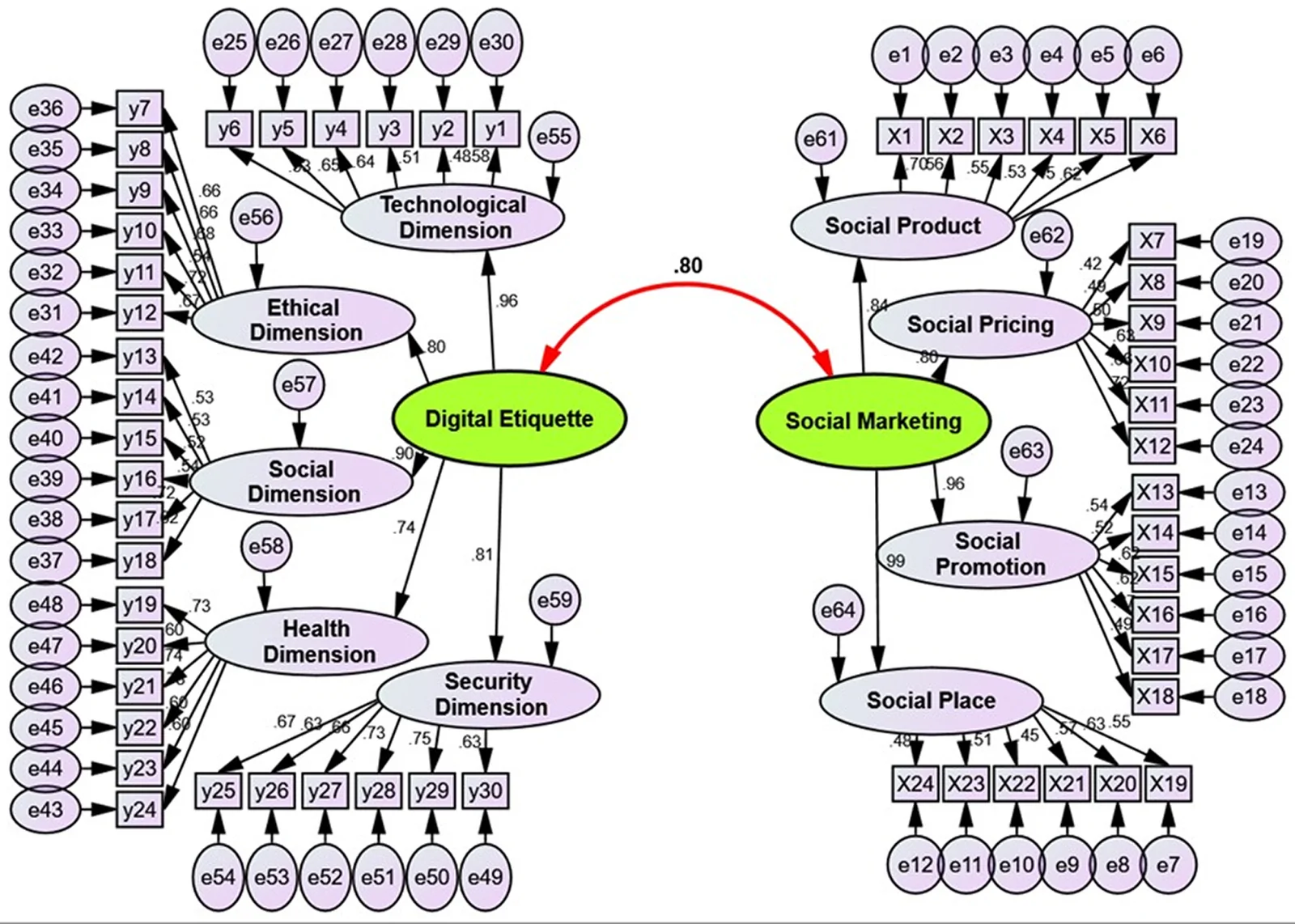
Effect of social marketing on digital etiquette.

The following sub-hypotheses emerge from the fourth main hypothesis:

**The first sub-hypothesis:** There is a statistically significant moral impact of the social product on the dimensions of digital etiquette (technological, ethical, social, health, security).

**The second sub-hypothesis:** There is a statistically significant moral impact of social pricing on the dimensions of digital etiquette (technological, ethical, social, health, security).

**Sub-hypothesis 3**: There is a statistically significant moral impact of social promotion on the dimensions of digital etiquette (technological, ethical, social, health, security).

**Fourth sub-hypothesis:** There is a statistically significant moral impact of social distribution on the dimensions of digital etiquette (technological, ethical, social, health, security).

Table 19 below shows the results of testing the sub-hypotheses above:

**Table 19.** Impact results for the dimensions of the social marketing variable in the digital etiquette variabl.

Independent variable	Direction of impact	Dependent variable	Regression coefficient Estimate(β)	Standard error of the regression coefficient Se.(β)	95% Confidence Interval		Probability value P-value	Result
**Social product**	**→**	**Digital etiquette**	0.522	0.132	0.307	0.809	0.003	acceptance
**Social pricing**	**→**		0.690	0.274	0.367	1.366	0.003	acceptance
**Social promotion**	**→**		0.764	0.192	0.479	1.198	0.003	acceptance
**Social place**	**→**		0.796	0.178	0.530	1.261	0.002	acceptance

It is noted that the effect of the social product dimension on the digital etiquette variable is a direct effect through the positive sign of the regression coefficient Estimate (β), which is due to the social product dimension, which has a value of (0.522), and the standard error value of the regression coefficient is (0.132). Also, both the lower (Lower) and upper (Upper) confidence limits (95% Confidence Interval), which were worth (0.809, 0.307), were similar in signals at a significant level (0.05). It is also noted that this effect is a morally significant effect through the P value of (0.003), which is less than (0.05), which means that there is a moral effect of the social product dimension in the digital etiquette variable.

It is also noted that the effect of the social pricing dimension on the digital etiquette variable is a direct effect through the positive sign of the regression coefficient Estimate (β), which is due to the social pricing dimension, which has a value of (0.690), and the standard error value of the regression coefficient is (0.274). Also, both the lower (Lower) and upper (Upper) confidence limits (95% Confidence Interval), whose values were (1.366, 0.367), were similar in signals at a significant level (0.05). It is also noted that this effect is a morally significant effect through the P value of (0.003), which is less than (0.05), which means that there is a moral effect of the social pricing dimension in the digital etiquette variable.

It is also noted that the effect of the social promotion dimension on the digital etiquette variable is a direct effect through the positive sign of the regression coefficient Estimate (β), which is due to the social promotion dimension, which has a value of (0.764), and the standard error value of the regression coefficient is (0.192). Also, both the lower (Lower) and upper (Upper) confidence limits (95% Confidence Interval), which had a value of (1.198, 0.479), were similar in signals at a significant level (0.05). It is also noted that this effect is a morally significant effect through the P value of (0.003), which is less than (0.05), which means that there is a moral effect of the social promotion dimension in the digital etiquette variable.

Finally, it is noted that the effect of the social place dimension on the digital etiquette variable is a direct effect through the positive sign of the regression coefficient Estimate (β), which is due to the social place dimension, which has a value of (0.796), and the standard error value of the regression coefficient is (0.178). Also, both the lower (Lower) and upper (Upper) confidence limits (95% Confidence Interval), which were worth (1.261, 0.530), were similar in signals at a significant level (0.05). It is also noted that this effect is a morally significant effect through the P value of (0.002), which is less than (0.05), which means that there is a moral effect of the social place dimension in the digital etiquette variable.

Figure 17 explains the effect of the dimensions of the social marketing variable on the digital etiquette variable.

**Figure 17.**
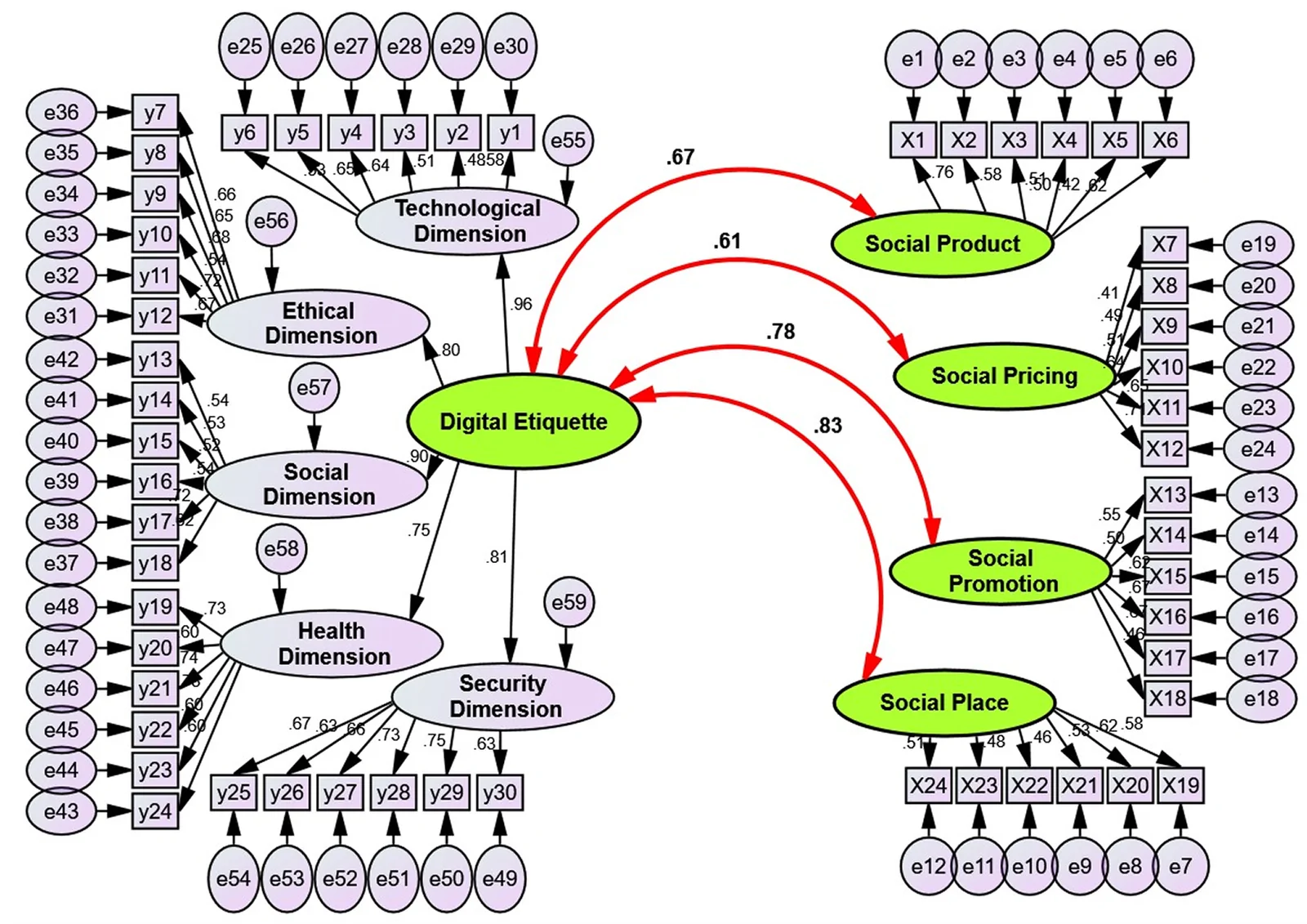
Effect of social marketing dimensions on digital etiquette.

### Statistical conclusions

1- In comparing the relative importance of the two variables studied, the digital etiquette variable appeared to be more relatively important than the social marketing variable in this study.
2- By measuring the stability of the questionnaire, it appeared that the value of the stratified alpha coefficient was 0.94, greater than 0.70, and this indicates the strength of stability of the dimensions studied.
3- By testing the bias of the common method, it became clear that there is no problem of bias in the common method.
4- By testing the return of the two variables to a society with a normal distribution, it became clear that the variables social marketing and digital etiquette do not have a normal probability distribution.
5- When conducting the confirmatory factor analysis, indicators of conformity quality were obtained, which appeared for the specified values, and no paragraph of the questionnaire was deleted.
6- When finding the effect, there appeared a moral effect of the social marketing variable on digital etiquette.
7- When finding the effect, there appeared a moral effect of all dimensions of the social marketing variable on the digital etiquette variable.

## Discussion

The results of the current study reflect the increasing level of awareness among Fallujah University students of the importance of adhering to digital etiquette in light of the shift towards digital education. These results are consistent with previous literature that confirmed that digital behaviors are greatly influenced by awareness practices, media campaigns, and social marketing programs adopted by educational institutions. It is noted that the social space and social promotion dimension having the highest impact coefficients indicates the importance of providing an accessible digital environment, supported by effective awareness messages. This consistency comes with what was indicated by ( Arenas-Escaso et al., 2024) and ( Saud, 2022) about the role of digital promotion in promoting responsible behavior within cyberspace The strength of the impact of social pricing on digital behavior also confirms that the investments made by the university in digital support tools, awareness programs, and reducing service costs are linked to improving students' commitment to ethical, health, and security behaviors.

On the part of students, a strong commitment has emerged in the technological and health dimension, which is consistent with the World Health Organization reports (2025) on the need to enhance awareness of the health risks associated with excessive use of technology, and shows students' readiness to respond to awareness initiatives.

In general, the study confirms that social marketing practices are not just promotional activities, but rather influential tools in shaping positive digital behaviors and promoting a responsible learning environment.

### Recommendations

Based on the results of the study and analysis of digital reality at the University of Fallujah, the researchers recommend the following:

1. Enhancing the adoption by Iraqi universities of social marketing practices that contribute to enhancing digital etiquette among students, by employing innovative methods in spreading digital behavioral awareness, such as the use of multimedia, awareness videos, and infographics, in a way that enhances the delivery of ethical messages to target groups more effectively.
2. Strengthening partnership between academic leaders (deanships of colleges, scientific departments, electronic media division) in consolidating digital values and responsibility.
3. Including mandatory training programs for new students that address digital etiquette, cybersecurity, and digital identity management, ensuring early awareness building among students about how to use educational platforms and digital technologies in a safe and ethical manner.
4. Investing in developing technological infrastructure and modernizing digital systems and platforms within universities, ensuring improved quality of digital interaction and enhancing a responsible behavioral environment.
5. Building human resources capabilities by training academic and administrative cadres on digital awareness methods, to ensure an effective and sustainable impact on student behavior.
6. Develop periodic monitoring and evaluation systems to measure the impact of social marketing practices on improving digital behavior, and identify the strengths and weaknesses of awareness programs, which helps improve them and direct them towards students' needs.
7. Expanding the study of other variables affecting digital behavior, such as digital well-being, technical culture, institutional and governmental support, and psychological and social factors, to allow for a deeper understanding of digital behavior in the university environment.
8. Conducting similar studies in other Iraqi universities for the purpose of comparison, which contributes to developing unified educational policies that promote ethical digital behavior at the national level.

## Ethical considerations

This study involved human participants and was conducted in accordance with accepted ethical research standards and the principles outlined in the Declaration of Helsinki. Ethical approval was obtained from the Scientific Research Ethics Committee, University of Fallujah, Iraq (Approval No. HOF.HUM.2025.001). Written informed consent was obtained from all participants prior to their participation. All participants were informed about the purpose of the study, the voluntary nature of their participation, their right to withdraw at any time without consequences, and the confidentiality of their data.

## Data Availability

The data supporting the findings of this study are openly available in Zenodo at: https://doi.org/10.5281/zenodo.18475239, Hammadi, A. A., & Reham, M. H. (2026). The Impact of Social Marketing Practices in Promoting Digital Etiquette A Descriptive and Analytical Study on a Sample of Fallujah University Students [Data set]. Zenodo These data are available under the terms of the Creative Commons Zero “No rights reserved” data waiver (CC0 4.0 Public Domain Dedication). This study is an observational survey-based research and follows the STROBE reporting guidelines. No CONSORT or ARRIVE checklists are required, as the study does not involve clinical trials or animal experiments.
