# The impact of social marketing practices in promoting digital etiquette: a descriptive and analytical study on a sample of Fallujah University students

**DOI:** 10.12688/f1000research.173990.2

**Published:** 2026-08-24

**Authors:** Reham Moatasem Hamza, Ahmed Abbas Hammadi

**Affiliations:** 1 Department of Public Administration, College of Administration and Economics, University of Fallujah, Iraq; 2 Department of Business Administration, College of Administration and Economics, University of Fallujah, Iraq

**Keywords:** social marketing practices, digital etiquette.

## Abstract

**Background:**

The research aims to know the role of social marketing practices in enhancing digital etiquette at the University of Fallujah, through a descriptive and analytical study on a sample of students amounting to (215) students. The research problem was the deficiency in some students’ commitment to digital etiquette. The importance of the study is highlighted in analyzing the impact of social marketing tools on raising awareness of ethical values on the Internet and elsewhere. The research recommends the necessity for Iraqi universities to adopt innovative social marketing practices to enhance digital responsibility, and develop technological infrastructure and human resources in line with the modern digital environment.

**Methodology:**

The research followed the descriptive analytical approach, and the data were collected via a questionnaire. The data were analyzed using the program (SPSS V.24) based on arithmetic means, standard deviations, the simple correlation coefficient (Pearson), and multiple linear regression testing to verify the influence relationships between the study variables.

**Results:**

Analytical results indicate that the university regularly adopts social marketing tools and activates its four dimensions (social product, social pricing, social promotion, and social distribution), which has had a positive impact on digital transformation. The data showed an increase in the level of commitment to digital etiquette among students by (25%) according to the approved digital behavior standards, with a significant moral impact (p < 0.05) of social marketing practices in promoting positive digital behaviors and reducing irresponsible behaviors.

**Conclusions:**

Social marketing practices play an important role in raising awareness among Fallujah University students about the importance of responsible digital behavior. The use of various methods such as awareness videos, graphics, and direct interaction with students also helped deliver ethical messages more effectively. The study confirms that social marketing is a real catalyst for social change in the digital environment.

## Introduction

The rapid evolution of digital technologies has reshaped communication and marketing practices by expanding the use of multiple digital channels. These platforms provide effective opportunities for influencing individuals’ attitudes and behaviors, making them valuable tools for social marketing initiatives aimed at promoting responsible digital practices and strengthening digital etiquette ( Al-Qotaje et al., 2026). As the world today is witnessing rapid transformations led by modern technology and the digitization of societies, which has made digital behavior an essential element in shaping the behavior of individuals ( Causio et al., 2025). Internet technologies have become an integral part of daily life, providing broad possibilities for communication, but at the same time they require high responsibility and commitment to digital etiquette as a socio-cultural phenomenon resulting from the development of digital civilization This development has contributed to reshaping traditional social values and norms, and has highlighted the need for digital etiquette as a normative and ethical regulator of communication in the virtual environment ( Issembayeva & Seyitkazy, 2025).

In contemporary literature, several theoretical frameworks have been proposed to explain digital behavior, the most prominent of which are the digital citizenship framework and behavior change theories. Digital citizenship focuses on identifying the knowledge, values, and skills necessary for responsible participation in the digital environment, while behavior change theories focus on explaining the psychological and cognitive factors that influence the adoption or avoidance of behavior. Despite the importance of these frameworks in explaining digital behavior, However, it focuses primarily on explaining behavior or determining its characteristics, and does not adequately provide practical intervention mechanisms through which educational institutions can influence and enhance the targeted behavior in the long term. In contrast, social marketing is characterized as an applied framework directed towards bringing about voluntary behavioral change that achieves social benefit. Social marketing gains its distinction from other frameworks explaining behavior through the reference standards set by Andreasen (2002). Which includes focusing on behavior change, conducting target audience research, segmentation, exchange, marketing mix, and analyzing behavioral competition. These criteria help distinguish between social marketing and other frameworks of behavioral or social change, because they are not limited to explaining behavior only, but rather provide practical mechanisms for designing interventions capable of influencing and enhancing the target behavior ( Suggs & Speranza, 2022). The success of digital transformation extends beyond the availability of technological infrastructure, as it is also shaped by organizational values and behavioral practices that encourage transparency, accountability, participation, and the responsible adoption of digital technologies ( Ghareeb et al., 2026). The effectiveness of digital environments extends beyond technological advancement, as it is also influenced by users’ trust, awareness, and responsible behavioral practices ( Awni et al., 2026). Accordingly, digital etiquette can be conceptualized as a critical behavioral framework that promotes ethical and responsible digital participation. In this context, social marketing is regarded as a strategic approach for shaping individuals’ attitudes, enhancing awareness, and encouraging socially desirable digital behaviors. From this perspective, social marketing is not adopted in the present study merely as an interpretive framework, but as an intervention-oriented framework that offers practical mechanisms for strengthening digital etiquette among university students, particularly within digitally mediated environments.

In light of the Iraqi Ministry of Higher Education and Scientific Research’s move towards digitization, local university education is witnessing a rapid shift towards a digital environment, which has produced new practices and behaviors among students within e-learning spaces. Despite the opportunities this shift provides for interaction, creativity, and knowledge exchange, it may reveal aspects of the shortcomings in some students’ commitment to digital etiquette Whether in communicating via academic platforms or in using electronic resources in a responsible and ethical manner, such as publishing inappropriate comments, sharing information without permission, etc. The US Development Bank report (2025) indicates that digital behaviors require users and developers to practice conscious and responsible behavior that protects users from misuse of technology, such as cyberbullying and exploitation of personal data, and in return promotes the development of technologies that support and enhance social welfare and human rights ( Latorre et al., 2025). The World Health Organization also directly confirmed this problem, as it indicated in its report issued in 2025 that: «Research has reinforced the existence of multiple online risks to the mental health, physical health, and well-being of young people, with a strong consensus that the stages of biological and cognitive development of children and adolescents make them particularly vulnerable to online harm» ( WHO, 2025). This reflects the complex nature of digital behavior and highlights the need for effective interventions that promote awareness and safe use of technology.

The current study was based on the Theory of Planned Behavior as an appropriate framework for explaining the role of social marketing practices in promoting digital etiquette, as it assumes that behavioral intention represents the direct determinant of behavior, and is affected by three main variables: orientation towards behavior, subjective standards, and perceived behavioral control ( Li et al., 2024). Trend refers to the individual’s evaluation of behavior, while subjective standards reflect the social pressures and expectations that influence his decisions, while perceived behavioral control expresses the individual’s awareness of his ability to practice behavior in light of the skills and resources he possesses. The theory confirms that enhancing these variables contributes to strengthening behavioral intention and increasing the likelihood of practicing the targeted behavior ( Pinheiro et al., 2023: 1880-1882). Studies have also indicated the importance of including moral commitment within the model to increase its ability to explain behaviors of an ethical nature (Li et al., 2024). Therefore, the impact of social marketing practices in promoting digital etiquette can be explained by creating positive attitudes towards responsible digital behavior, promoting social norms that support it, and developing individuals’ awareness of their ability to adhere to it. Bandora’s Social Learning Theory is also a complementary framework for explaining the acquisition of desired digital behaviors, as it assumes that individuals learn through observation, imitation, and modeling within the social environment ( Al-Mandlawi, 2024). The learning process takes place through four main stages: attention, retention, imitation, and motivation ( Istiqomah, 2021). From this perspective, social marketing practices can be viewed as tools that contribute to disseminating and enhancing positive digital behavioral models, helping individuals acquire digital etiquette through observation, imitation, and social reinforcement. This makes the theories of planned behavior and social learning a suitable theoretical framework for explaining the relationship between social marketing practices and digital etiquette.

In this context, social marketing practices have emerged as an influential approach for promoting positive behavioral change. By encouraging socially desirable behaviors and discouraging harmful ones, these practices contribute to improving individual well-being and enhancing the welfare of society as a whole ( Galiano-Coronil et al., 2024). This growing recognition has increased scholarly interest in understanding the theoretical foundations and historical development of social marketing.

The origins of social marketing can be traced back to the early 1950s, when Wiebe raised the question of whether marketing principles could be applied to address social problems by asking, “Why can’t brotherhood be sold like soap?” In an article published in Public Opinion Quarterly during the winter of 1951–1952, he argued that applying marketing practices in nonprofit campaigns could contribute to more effective solutions to social problems, thereby laying the foundation for the emergence of social marketing as a distinct field of study ( Joy, 2019).

Social marketing seeks to promote the public good by encouraging positive behavioral change through the application of behavioral, psychological, and marketing principles to address societal problems ( Shekhar & Venugopal, 2025). Its importance also lies in raising public awareness of social and health ills through field campaigns that aim to bring about change in public behavior and enhance its awareness of issues affecting society ( Hasnaa & Ryan, 2023). In the same context, social marketing was established as a strategy to address social problems, as it reflects the application of advanced marketing techniques to social issues that can be solved through behavior change. It has been used in many successful projects that aimed to change healthy behavior within society, in addition to its role in creating supportive public opinion that motivates society to participate positively in solving the problems it faces This is done by encouraging individuals to move away from negativity and indifference and enhancing their role in bringing about change and development within society ( Arif & Ahmed, 2025).

Khatti-Dizabadi et al. (2024) identified four key dimensions of social marketing practices: social product, social pricing, social distribution, and social promotion. The social product may take the form of a service, an idea, or a desired behavioral change, such as initiatives aimed at reducing digital stress, limiting excessive smartphone use, and promoting digital well-being. In this regard, social products play an important role in fostering digital etiquette by encouraging individuals to adopt responsible online behaviors and adhere to ethical standards in digital interactions, thereby contributing to a safer and more respectful digital environment ( Arenas-Escaso et al., 2024; Orzikulova et al., 2024).

The social product also refers to the issue or topic that the campaign seeks to promote or change, as it must be relevant to the needs and problems of society and provide effective solutions to improve the lives of individuals ( Kharfan & Khairuddin, 2024). Within the framework of recent digital developments, Orzikulova et al. (2024) developed Time2Stop, an intelligent and adaptive system based on artificial intelligence to reduce the overuse of smartphones, which represents an example of modern digital social products. A study ( Arenas-Escaso et al., 2024) also indicated that digital initiatives such as free digital tourism can contribute to enhancing social well-being and improving quality of life. The social price, it is represented by the effort made to adopt the new behavior ( Kegoro, 2020), such as self-control to use the phone, which is an influential factor in reducing over-reliance on technology ( Li et al., 2025), The role of pricing in social marketing strategy is often secondary, as social campaigns mainly aim to educate the consumer and rationalize his behavior, in addition to serving individuals and society. That is, the primary goal is not to achieve profits, and if some social products are priced, this is often a symbolic price to cover costs, and sometimes to give appreciation and respect to this product ( Hasnaa and Ryan, 2023).

While social promotion (Social Promotion) focuses on using various means of communication to spread awareness and influence the behavior of individuals in a way that supports the achievement of social goals. Fayaz (2022) showed that promotion is the process of delivering information to the target audience, and a study ( Zhao et al., 2024) indicated that the PauseNow application represents a digital intervention to enhance digital well-being, as it promotes digital healthy behavior through continuous reminders and alerts that redirect the user towards his original intentions,Social promotion focuses on raising awareness by using media and awareness messages to promote healthy digital behaviors through preventive digital applications and interventions ( Saud, 2022; Akbar et al., 2023; Zhao et al., 2024; Wu et al., 2025).

Kegoro (2020) focused on the fact that the social distribution dimension is measured by indicators such as adaptability, consumption time, and delivery method, ensuring that interventions reach the target group effectively. For example, Apple and Google have introduced a range of features and apps to help reduce distraction and enhance the user’s digital well-being, such as SPACE and GDW apps, which provide a digital environment that helps users eliminate technology distraction and balance phone and life use ( Almourad et al., 2021). The study ( Sivakumar et al., 2023) aims to investigate the effectiveness of social media as digital channels for knowledge exchange between college and university students, and its role in supporting the educational process. The results showed that digital platforms represent an effective means of disseminating information and exchanging knowledge, enhancing students’ motivation and improving their academic performance, and providing an appropriate digital environment to encourage students’ participation and interaction Making it a living example of effective digital distribution channels for social products and services in higher education institutions.

In today’s interconnected world, understanding digital etiquette is essential to maintaining safe and respectful online interactions ( Ab Wahab, 2024), and after reviewing many studies, research and scientific literature related to the concept of digital etiquette such as the study of Abdul Tawab (2020), Al-Shehri (2021), and Haroun and Hassouna (2022). The researchers summarized it in five basic dimensions that represent the main axes on which digital behavior is built. The technological dimension is represented in the individual’s awareness of using information technology, and his mastery of the rules of digital communication such as writing email and using platforms in a professional manner, while the ethical dimension is concerned with adhering to ethical rules derived from religion, law, and societal norms, while respecting privacy and the rights of others, avoiding abuse, protecting personal information, and reviewing content before publishing it to ensure that it is not misused ( Abdel Tawab, 2020; Al-Shehri, 2021; Haroun & Hassouna, 2022). The social dimension includes polite behaviors that regulate individuals’ interaction within the digital environment, such as taking responsibility, effective communication, respecting other opinions, and positive participation that reflects the spirit of cooperation within the digital community ( Abdul Tawab, 2020; Al-Shehri, 2021; Haroun & Hassouna, 2022). Social media is a tool for instilling social values and teaching acceptable interaction styles ( Neumann & Rhodes, 2023), but it can present challenges such as overreliance and digital bullying in its various forms, making digital moral and social education essential to ensure respectful and responsible interaction within the digital space ( Rini et al., 2023). The health dimension includes the behaviors that individuals should practice while using digital technology, as part of digital etiquette, such as healthy sitting, organizing the duration of use, and paying attention to basic meals to ensure a balance between healthy living and digital use. This dimension is linked to awareness of the risks resulting from excessive use of technology, as it may lead to depression, anxiety, isolation, and sleep disorders. The World Health Organization has classified Internet addiction as one of the global problems affecting sleep, motivation, memory, relationships, and quality of life, due to its similarity to addiction to harmful substances ( Dresp-Langley & Hutt, 2022). To address these effects, healthy behavior change (HBC) interventions have emerged that adopt psychological and behavioral approaches to improve diet, physical activity, sleep, and reduce the use of harmful substances. Although traditional interventions are effective, they are expensive, making digital alternatives more scalable ( Sawyer et al., 2023). Digital health awareness has also emerged as a new determinant of health, enabling individuals to use digital technologies to access health information and employ it in problem solving, reflecting the integration of health awareness with digital awareness ( Qiu et al., 2025). The security dimension is concerned with protecting accounts, personal information, and files from any unauthorized interference or access ( Haroun & Hassouna, 2022) and is increasingly important in the digital higher education environment, which requires advanced awareness of cyber security to ensure the safe use of technologies. Information security is based on the principles of the Triangle (CIA): confidentiality, integrity, availability, non-repudiation and credibility as elements that enhance digital trust. Effective awareness of information security based on understanding personal protocols and responsibilities is also an essential element for protecting organizations, and is not limited to measuring awareness, but includes evaluating the impact of security initiatives in reducing human errors and promoting preventive behaviors ( Bognár & Bottyán, 2024).

Despite the growing interest in studying digital etiquette within educational settings, most previous studies have primarily focused on describing the level of digital behavior among students or analyzing its influencing factors, without moving toward adopting applied approaches capable of achieving actual and sustainable behavioral change within the digital university environment. In contrast, the literature on social marketing has largely concentrated on traditional areas such as health, environmental issues, and preventive behaviors, with limited attention given to its application in the field of digital behavior, particularly digital etiquette. Furthermore, there is a noticeable lack—within the researchers’ knowledge of Arab and Iraqi studies that have directly examined the relationship between the dimensions of social marketing practices (social product, price, promotion, and distribution) and digital etiquette among university students. Accordingly, there is a need to develop a framework that links these two variables and to empirically test this relationship within a local digital academic context, which the current study seeks to address. In light of the above, it is important and necessary to conduct this research in a new local and digital environment and among new users, namely students of the University of Fallujah in Iraq, with the aim of building responsible digital awareness. Here, a major important question arises that can be raised: (What is the role of social marketing practices in enhancing digital behavior?), and how, through these practices, students can be directed towards using technology and digital platforms in a responsible, healthy and safe manner, which contributes to developing a digital educational environment based on respect, awareness and commitment to digital etiquette.

Therefore, the theoretical contribution of the current study is to expand the scope of social marketing applications from traditional fields related to health and the environment to the digital and educational field, by testing its ability to explain and enhance digital etiquette among university students, which contributes to enriching the literature related to digital behavior and provides a theoretical basis that can be relied upon in future studies.

## Methodology

### Sample size determination procedure

Each proposed structural model requires a specific sample size. To determine the appropriate sample size for the proposed model, the website provided in the following link was used ( Westland, J. C., 2010), which is widely applied in scientific research. The website requires specifying the number of questionnaire items and the number of dimensions for each main construct. Based on these inputs, the website determines the minimum required sample size for the proposed structural model, as presented in Table 1.

**Table 1.** Determining the minimum sample size required for applying structural equation modeling.

Inputs	Accepted values	Selected values
Anticipated Effect Size	0.10 = Small 0.30 = Medium 0.50 = Large	0.30
Desired Statistical Power Level	Should not be less than 0.80	0.80
Number of Latent Variables (Dimensions)	—	11
Number of Observed Variables (Questionnaire Items)	—	66
Probability Level (Significance Level)	0.01 0.05 0.10	0.05
Outputs		
Minimum Sample Size to Detect Effect	195	
Minimum Sample Size for Model Structure	200	
Recommended Minimum Sample Size	200	

The minimum required sample size for the proposed structural model was determined to be 200 respondents. Table 1 illustrates the required information entered into the website to estimate the appropriate sample size. Accordingly, 225 questionnaires were randomly distributed among the selected sample to achieve greater accuracy and reliability of the research results. A total of 220 questionnaires were retrieved from respondents. After excluding incomplete questionnaires, 215 valid responses were obtained and used for the statistical analysis.

### Type, design and scope

This study relied on the descriptive analytical approach, due to its ability to describe and analyze the phenomena under study. First, the research variables represented in social marketing practices (social product, social pricing, social promotion, social distribution) and digital etiquette were described in its dimensions (technological dimension, ethical dimension, social dimension, health dimension, security dimension), through a questionnaire form designed for this purpose. The data were then analyzed statistically using appropriate statistical methods to extract indicators and interpret relationships between variables, allowing for a deeper understanding and clear vision of the current reality of the Iraqi universities under study.

### Community and sample

The research community consists of all students of Fallujah University, as they represent the target group for studying the impact of social marketing practices in promoting digital etiquette. A comprehensive inventory method was adopted in selecting the sample, so that the sample included a large number of students to ensure the inclusion of all units related to the subject of the study and to obtain accurate data that reflects the actual reality of digital behavior among students within the university.

### Procedures

According to the research design, the research followed logical and systematic steps, which facilitated the solution of the research problem. The most important research steps included the following points: conceptualizing the idea, stating the problem, designing the research, selecting the sample, collecting data, analyzing it, interpreting it, and discussing the results.

### Data collection techniques and tools

Considering the dimensions and indicators of the study, the questionnaire tool was adopted as the main means of collecting data. This tool was developed to include the social marketing practices variable consisting of (24) items, distributed over its dimensions, with the adoption of the five-point Likert scale to determine the level of response. As for the digital etiquette variable, it included (30) paragraphs, distributed according to its dimensions, and using the same scale. The questionnaire was applied to the study sample members with the aim of obtaining the data necessary for statistical analysis.

### Analyze the results

The study aimed to determine the impact relationship between social marketing practices and digital etiquette for a sample of Fallujah University students. The study was conducted based on questionnaire data, and according to the research variables, the following was observed:

### First: Describe the opinions of the researched sample

This paragraph will describe the identifying information of the respondents, as shown in Table 2 below:

1- **Demographic information**
  ➢ **Gender**: Males accounted for 55% of the respondents, while females accounted for 45%. This demonstrates that the representation of males and females is equal at the university under study, as outlined in Figure 1.
  ➢ **Age**: It is noted that the age group (20 years and younger) obtained a percentage of 4.7%, while the age group (25-21 years) obtained a percentage of 47%, the age group (under 30 years) obtained a percentage of 32.1%, and finally the age group (30 years and older) obtained a percentage of 16.2% for respondents, as outlined in Figure 2.
  ➢ **Academic qualification**: It is noted that the preparatory academic qualification obtained a percentage of 28.8%, the bachelor’s academic qualification obtained a percentage of 46%, while the master’s academic achievement obtained a percentage of 25.2% for respondents.
The identifying information can be explained conceptually, as outlined in Figure 3.
2- **Describe and diagnose the study variables**
The process of describing and diagnosing the study variables at the macro level and in terms of their dimensions is one of the important steps to determine the level of orientation and tendencies of the researched individuals towards those variables and dimensions. These tendencies are usually measured through the percentage of agreement and the relative importance index (Relative importance index (RII)). This index is used to know the relative importance of the paragraphs and dimensions of the study from the point of view of the research sample in the researched organization. Note that the value of this indicator falls between (0≤RII≤1) and its value can be classified into five levels according to the five-point Likard scale adopted in our study, as shown in Table 3.

**Table 2.** Description of the researched individuals.

Description	Division or category	Number	Ratio
**Gender**	**Male**	**118**	**55%**
	**Female**	**97**	**45%**
	**Total**	**215**	**100%**
**Age**	**20 years and younger**	**10**	**4.7%**
	**25-21 years**	**101**	**47%**
	**Under 30 years old**	**69**	**32.1%**
	**30 years or older**	**35**	**16.2%**
	**Total**	**215**	**100%**
**Academic qualification**	**Preparatory**	**62**	**28.8%**
	**Undergraduate**	**99**	**46%**
	**Master's**	**54**	**25.2%**
	**Total**	**215**	**100%**

**Figure 1.**
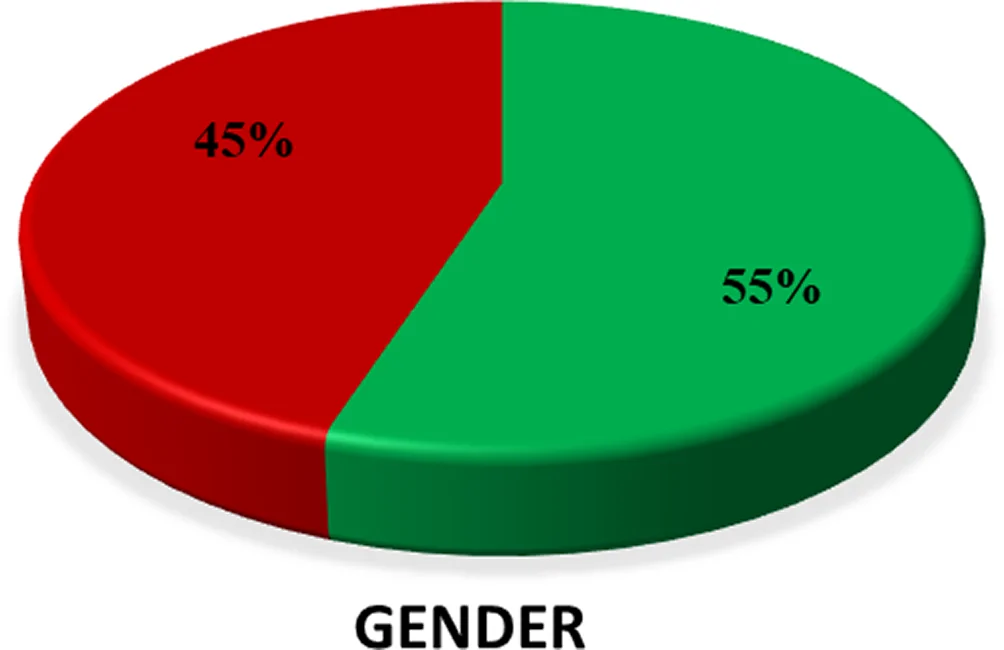
Percentages of identifying information about the gender variable.

**Figure 2.**
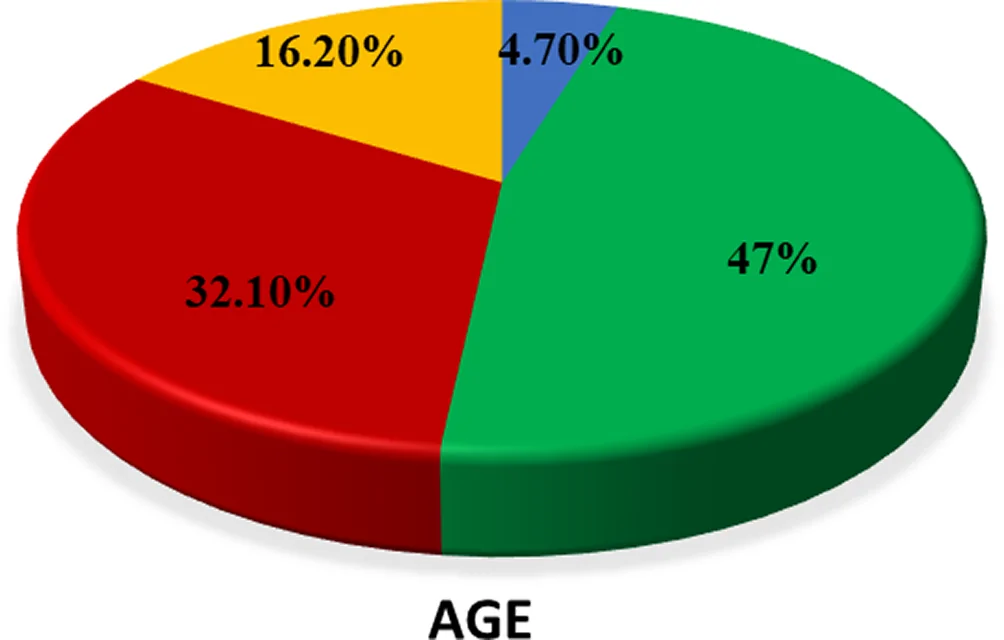
Percentages of identifying information about the age variable.

**Figure 3.**
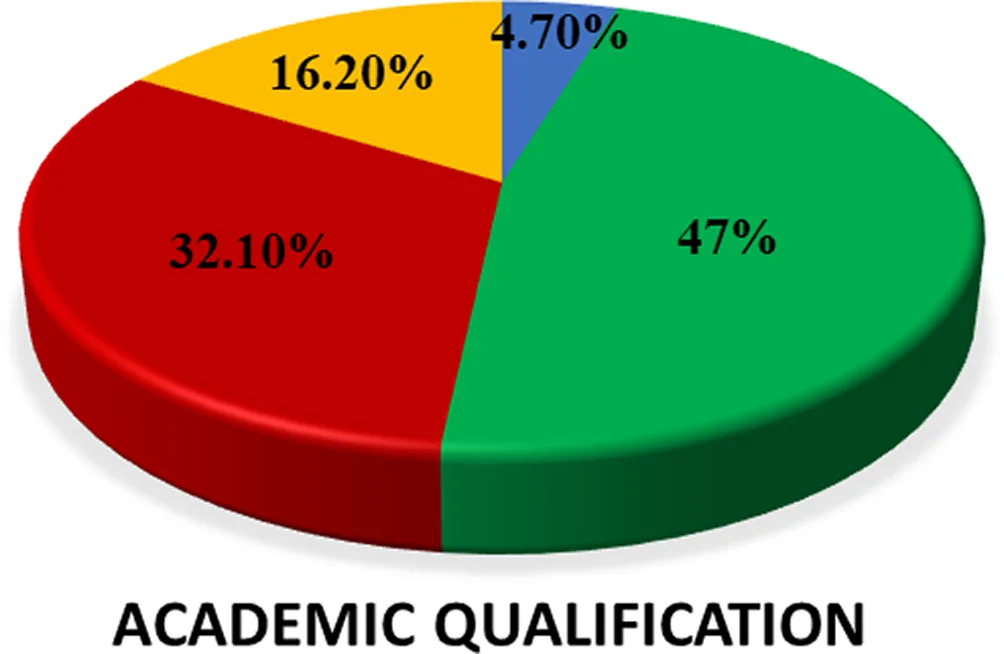
Percentages of identifying information about the Academic qualification variable.

**Table 3.** Levels of importance according to the five-point Likard scale.

Degree of relative importance	RII
*High*	*0.8* ≤ *RII* ≤ *1*
*Medium to high*	*0.6* ≤ *RII < 0.8*
*Medium*	*0.4* ≤ *RII < 0.6*
*Low to medium*	*0.2* ≤ *RII < 0.4*
*Low*	*0* ≤ *RII < 0.2*

1- **Describe and diagnose the independent variable social marketing**
  ➢ **Social product**
The basic statistical measures were found in order to describe and diagnose the paragraphs of the social product dimension, which included paragraphs (X1-X6). Table 4 explains this description. The average dimension was (3.867), while the standard deviation was (0.765) and of relative importance (77.348%), as this percentage is classified as a medium to high percentage as an indicator of relative importance (RII). As for the percentages of (agreement), opinion (agreed to some extent), and (disagreement), their percentages reached (73.93%), (21.57%), and (4.50%), respectively. Note that the highest paragraph is X1 (Fallujah University provides sufficient information about its services that contribute to meeting the social needs of students and subscribers), and the lowest paragraph is X5 (Fallujah University seeks to continuously develop and improve its services to advance the social aspect of students and the surrounding society). Figure 4 explains illustrates the of relative importance of each item in the social product.
  ➢ **Social pricing**
Basic statistical measures were found in order to describe and diagnose paragraphs of the social pricing dimension, which included paragraphs (X7-X12). Table 5 explains this description. The average dimension was (3.760), while the standard deviation was (0.833) and of relative importance (75.194%), as this percentage is classified as a medium to high percentage as an indicator of relative importance (RII). As for the percentages of (agreement), opinion (agreed to some extent), and (disagreement), their percentages reached (70.77%), (20.00%), and (9.23%), respectively. Note that the highest paragraph is X7 (Fallujah University offers attractive facilities and discounts to students and beneficiaries as a type of practice that supports society), and the lowest paragraph is X10 (the prices of services and activities provided by Fallujah University are proportional to the purchasing power of students and beneficiaries). Figure 5 explains illustrates the of relative importance of each item in the social pricing.
  ➢ **Social promotion**
Basic statistical measures were found in order to describe and diagnose paragraphs of the social promotion dimension, which included paragraphs (X13-X18). Table 6 explains this description. The average dimension was (3.890), while the standard deviation was (0.779) and of relative importance (77.798%), as this percentage is classified as a medium to high percentage as an indicator of relative importance (RII). As for the percentages of (agreement), opinion (agreed to some extent), and (disagreement), their percentages reached (74.55%), (19.78%), and (5.67%), respectively. Note that the highest paragraph is X13 (Fallujah University displays advertisements that include sufficient information about the services and offers it provides to students and the community), and the lowest paragraph is X14 (Fallujah University is committed to credibility in advertisements directed to students and beneficiaries, as a way to avoid any marketing deception towards the community). Figure 6 explains illustrates the of relative importance of each item in the social promotion.
  ➢ **Social place**
Basic statistical measures were found in order to describe and diagnose paragraphs of the social space dimension, which included paragraphs (X19-X24). Table 7 explains this description. The average dimension was (3.912), while the standard deviation was (0.796) and of relative importance (78.232%), as this percentage is classified as a medium to high percentage as an indicator of relative importance (RII). As for the percentages of (agreement), opinion (agreed to some extent), and (disagreement), their percentages reached (75.33%), (19.57%), and (5.10%), respectively. Note that the highest paragraph is X19 (Fallujah University provides extensive coverage in all regions and the services it provides, even if this requires additional costs), and the lowest paragraph is X20 (Fallujah University’s website is easily accessible by students and beneficiaries). Figure 7 explains illustrates the of relative importance of each item in the social place.
2- **Describe and diagnose the independent variable of digital etiquette**
  ➢ **Technological dimension**
Basic statistical measures were found in order to describe and diagnose paragraphs of the technological dimension, which included paragraphs (y1-y6). Table 8 explains this description. The average dimension was (4.044), while the standard deviation was (0.742) and of relative importance (80.884%), as this percentage is classified as a high percentage as an indicator of relative importance (RII). As for the percentages of (agreement), opinion (agreed to some extent), and (disagreement), their percentages reached (80.07%), (16.42%), and (3.52%), respectively. Note that the highest paragraph is y2 (turn on the camera or microphone during simultaneous meetings for remote communication applications, at the appropriate time), and the lowest paragraph is y6 (speak tactfully when communicating with others in remote communication applications). Figure 8 explains illustrates the of relative importance of each item in the technological dimension.
  ➢ **Moral dimension**
Basic statistical measures were found in order to describe and diagnose paragraphs of the moral dimension, which included paragraphs (X13-X18). Table 9 explains this description. The average dimension was (3.733), while the standard deviation was (0.852) and of relative importance (74.667%), as this percentage is classified as a medium to high percentage as an indicator of relative importance (RII). As for the percentages of (agreement), opinion (agreed to some extent), and (disagreement), their percentages reached (65.97%), (25.02%), and (9.02%), respectively. Note that the highest paragraph is y10 (I do not publish unethical posts that offend others), and the lowest paragraph is y7 (I respect the privacy of electronic platforms and do not mishandle them). Figure 9 explains illustrates the of relative importance of each item in the moral dimension.
  ➢ **Social dimension**
Basic statistical measures were found in order to describe and diagnose paragraphs of the social dimension, which included paragraphs (X13-X18). Table 10 explains this description. The average dimension was (4.018), while the standard deviation was (0.811) and of relative importance (80.357%), as this percentage is classified as a high percentage as an indicator of relative importance (RII). As for the percentages of (agreement), opinion (agreed to some extent), and (disagreement), their percentages reached (81.00%), (14.42%), and (4.58%), respectively. Note that the highest paragraph is y16 (post posters and awareness bulletins on social media), and the lowest paragraph is y13 (commit to addressing individuals in the virtual world in a decent manner). Figure 10 explains illustrates the of relative importance of each item in the social dimension.
  ➢ **Health dimension**
Basic statistical measures were found to describe and diagnose the health dimension items, which included items (X13-X18). Table 11 explains this description. The average dimension was (4.126), while the standard deviation was (0.770) and of relative importance (82.512%), as this percentage is classified as a high percentage as an indicator of relative importance (RII). As for the percentages of (agreement), opinion (agreed to some extent), and (disagreement), their percentages reached (83.53%), (12.87%), and (3.60%), respectively. Note that the highest paragraph is y24 (I isolate myself from others when dealing with cyberspace), and the lowest paragraph is y19 (I am aware of the risks inherent when using digital technology). Figure 11 explains illustrates the of relative importance of each item in the health dimension.
  ➢ **Security dimension**
Basic statistical measures were found in order to describe and diagnose paragraphs of the security dimension, which included paragraphs (X13-X18). Table 12 explains this description. The average dimension was (3.884), while the standard deviation was (0.824) and of relative importance (77.674%), as this percentage is classified as a medium to high percentage as an indicator of relative importance (RII). As for the percentages of (agreement), opinion (agreed to some extent), and (disagreement), their percentages reached (74.95%), (19.23%), and (5.82%), respectively. Note that the highest paragraph is y26 (when dealing with devices and cyberspace, I put passwords that are difficult to predict), and the lowest paragraph is y30 (stay away from using anonymous programs, because they obtain information without the permission of their owner). Figure 12 explains illustrates the of relative importance of each item in the security dimension.

**Table 4.** Frequency distributions, arithmetic means, standard deviations, and the relative importance of the social product dimension.

Paragraphs	Response										Average	Standard deviation	Response intensity %	Ranking
	Totally agree (5)		Agree (4)		Neutral (3)		I don't agree (2)		Don't totally agree (1)					
	Number	%	Number	%	Number	%	Number	%	Number	%				
**X1**	43	20	129	60	39	18.1	3	1.4	1	0.5	3.977	0.693	79.534	1
**X2**	46	21.4	121	56.3	41	19.1	7	3.2	0	0	3.958	0.732	79.162	2
**X3**	34	15.8	122	56.7	50	23.3	8	3.7	1	0.5	3.837	0.747	76.744	4
**X4**	40	18.6	123	57.2	44	20.5	7	3.2	1	0.5	3.902	0.746	78.046	3
**X5**	31	14.3	116	54	52	24.2	12	5.6	4	1.9	3.735	0.842	74.698	6
**X6**	38	17.7	111	51.6	52	24.2	12	5.6	2	0.9	3.80	0.83	75.906	5
**Overall rate**		17.97		55.97		21.57		3.78		0.72	3.867	0.765	77.348	
**Total**	73.93				21.57		4.50							

**Figure 4.**
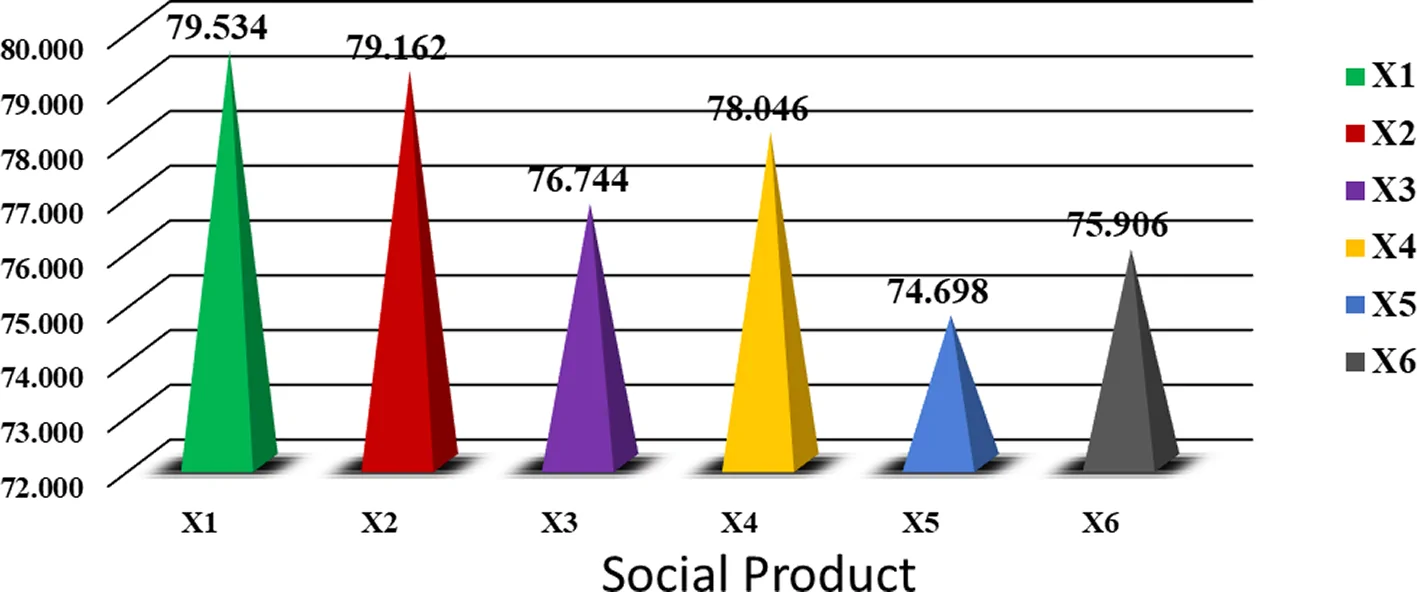
Explains illustrates the relative importance of each item in the social product.

**Table 5.** Description of the social pricing dimension and its results.

Paragraphs	Response										Average	Standard deviation	Response intensity %	Ranking
	Totally agree (5)		Agree (4)		Neutral (3)		I don't agree (2)		Don't totally agree (1)					
	Number	%	Number	%	Number	%	Number	%	Number	%				
**X7**	43	20	116	54	35	16.2	18	8.4	3	1.4	3.828	0.893	76.558	1
**X8**	27	12.6	108	50.2	52	24.2	23	10.7	5	2.3	3.600	0.921	72.000	5
**X9**	32	14.9	132	61.3	41	19.1	10	4.7	0	0	3.865	0.714	77.302	4
**X10**	23	10.7	110	51.1	50	23.3	31	14.4	1	0.5	3.572	0.882	71.442	6
**X11**	39	18.1	117	54.5	44	20.5	13	6	2	0.9	3.828	0.828	76.558	2
**X12**	34	15.8	132	61.4	36	16.7	12	5.6	1	0.5	3.87	0.760	77.302	3
**Overall rate**		15.35		55.42		20.00		8.30		0.93	3.760	0.833	75.194	
**Total**	70.77				20.00		9.23							

**Figure 5.**
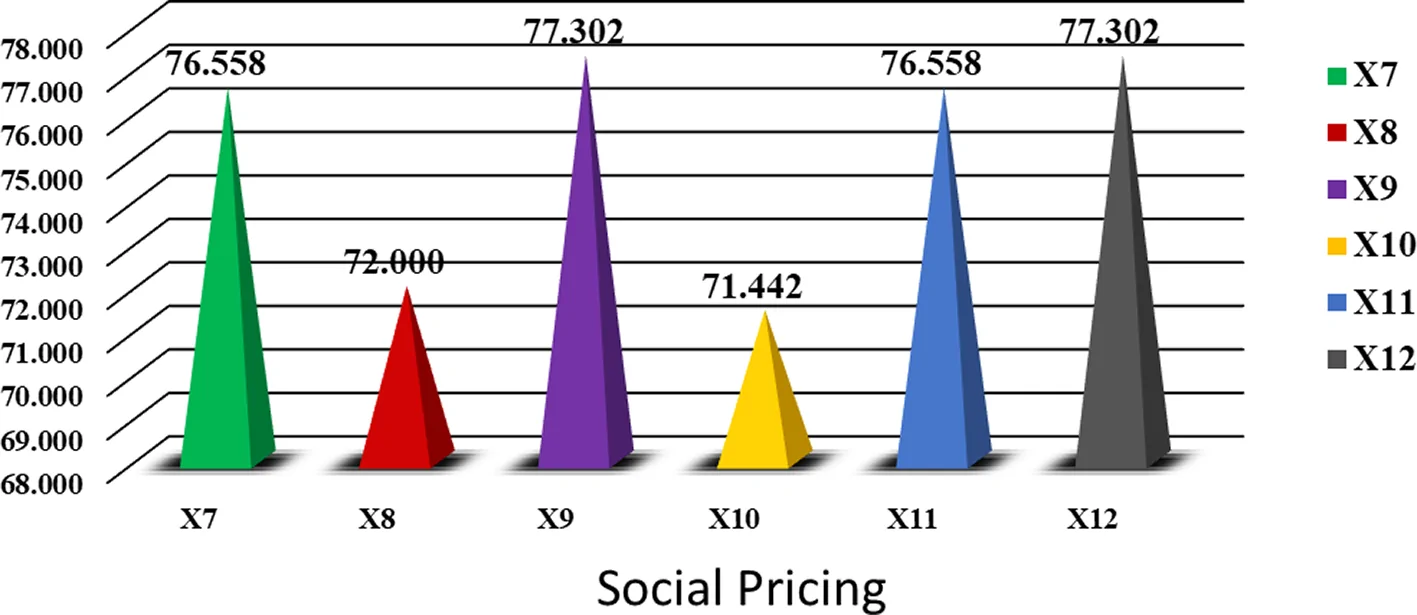
Explains illustrates the relative importance of each item in the social pricing.

**Table 6.** Frequency distributions, arithmetic averages, standard deviations, and the relative importance of the social promotion dimension.

Paragraphs	Response										Average	Standard deviation	Response intensity %	Ranking
	Totally agree (5)		Agree (4)		Neutral (3)		I don't agree (2)		Don't totally agree (1)					
	Number	%	Number	%	Number	%	Number	%	Number	%				
**X13**	58	27	122	56.7	26	12.1	8	3.7	1	0.5	4.061	0.762	81.210	1
**X14**	28	13	98	45.6	67	31.2	19	8.8	3	1.4	3.600	0.874	72.000	6
**X15**	55	25.6	115	53.5	36	16.7	6	2.8	3	1.4	3.991	0.815	79.814	2
**X16**	51	23.7	113	52.5	41	19.1	9	4.2	1	0.5	3.949	0.798	78.976	4
**X17**	37	17.2	117	54.4	47	21.9	13	6	1	0.5	3.819	0.803	76.372	5
**X18**	40	18.6	128	59.5	38	17.7	8	3.7	1	0.5	3.92	0.74	78.418	3
**Overall rate**		20.85		53.70		19.78		4.87		0.80	3.890	0.799	77.798	
**Total**	74.55				19.78		5.67							

**Figure 6.**
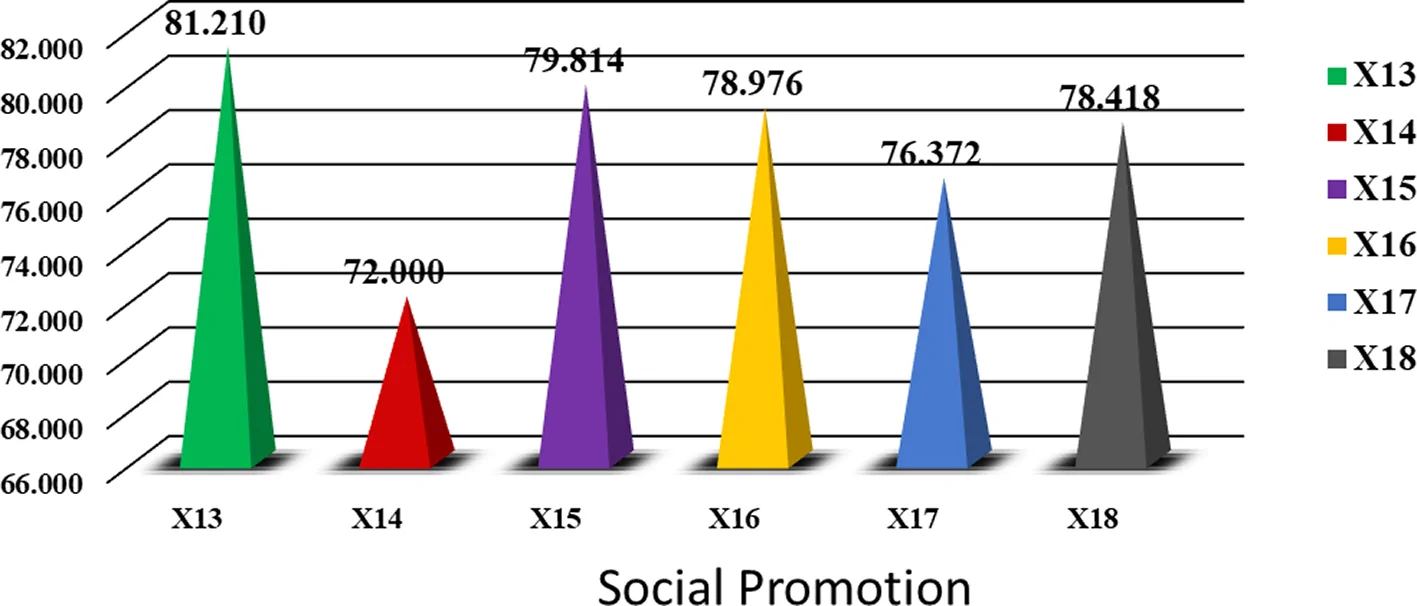
Explains illustrates the relative importance of each item in the social promotion.

**Table 7.** Frequency distributions, arithmetic means, standard deviations, and the relative importance of the social space dimension.

Paragraphs	Response										Average	Standard deviation	Response intensity %	Ranking
	Totally agree (5)		Agree (4)		Neutral (3)		I don't agree (2)		Don't totally agree (1)					
	Number	%	Number	%	Number	%	Number	%	Number	%				
**X19**	60	27.9	121	56.3	29	13.5	3	1.4	2	0.9	4.088	0.740	81.768	1
**X20**	38	17.7	102	47.4	57	26.5	15	7	3	1.4	3.730	0.882	74.604	6
**X21**	29	13.5	117	54.4	61	28.4	6	2.8	2	0.9	3.767	0.750	75.348	5
**X22**	58	27	114	53	30	14	11	5.1	2	0.9	4.000	0.837	80.000	3
**X23**	42	19.5	118	54.9	39	18.2	14	6.5	2	0.9	3.856	0.839	77.116	4
**X24**	54	25.1	119	55.3	36	16.8	6	2.8	0	0	4.03	0.73	80.558	2
**Overall rate**		21.78		53.55		19.57		4.27		0.83	3.912	0.796	78.232	
**Total**	75.33				19.57		5.10							

**Figure 7.**
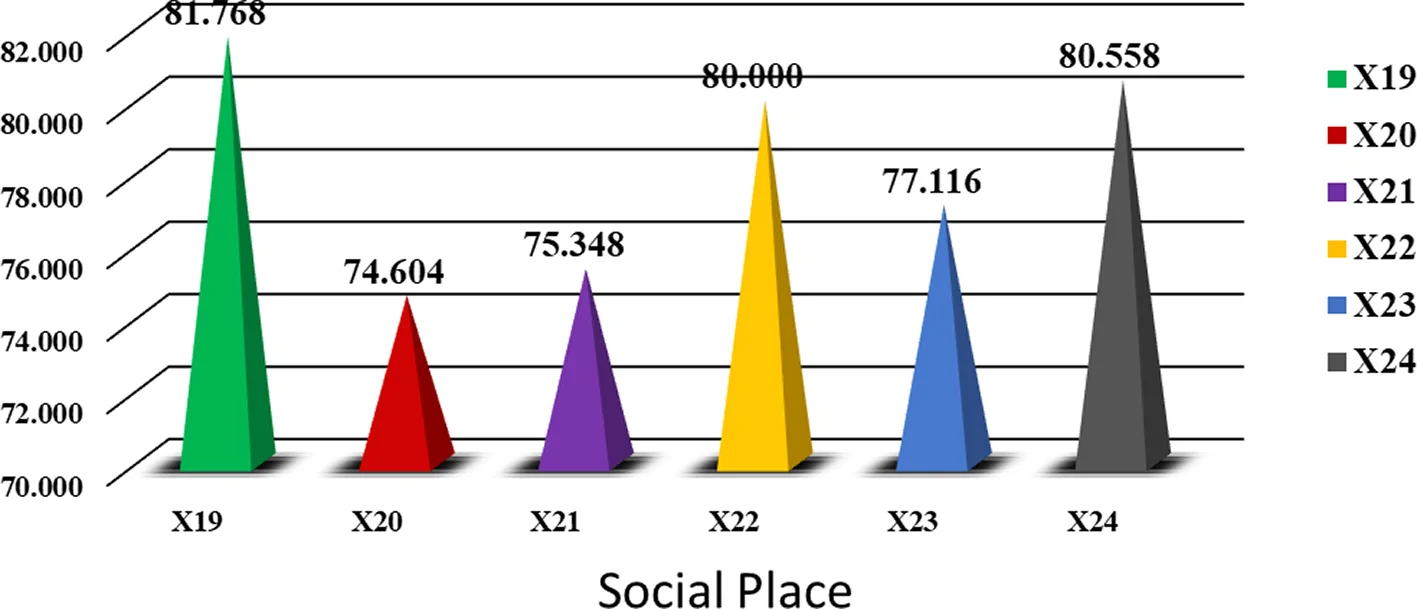
Explains illustrates the relative importance of each item in the social place.

**Table 8.** Frequency distributions, arithmetic means, standard deviations, and the relative importance of the technological dimension.

Paragraphs	Response										Average	Standard deviation	Response intensity %	Ranking
	Totally agree (5)		Agree (4)		Neutral (3)		I don't agree (2)		Don't totally agree (1)					
	Number	%	Number	%	Number	%	Number	%	Number	%				
**y1**	58	27	128	59.5	26	12.1	3	1.4	0	0	4.121	0.659	82.418	3
**y2**	83	38.6	109	50.7	21	9.7	1	0.5	1	0.5	4.265	0.690	85.302	1
**y3**	82	38.1	110	51.2	21	9.8	2	0.9	0	0	4.265	0.669	85.302	2
**y4**	59	27.4	109	50.7	42	19.5	4	1.9	1	0.5	4.028	0.767	80.558	4
**y5**	51	23.7	123	57.2	33	15.3	7	3.3	1	0.5	4.005	0.752	80.094	5
**y6**	32	14.9	89	41.4	69	32.1	22	10.2	3	1.4	3.58	0.91	71.628	6
**Overall rate**		28.28		51.78		16.42		3.03		0.48	4.044	0.742	80.884	
**Total**	80.07				16.42		3.52							

**Figure 8.**
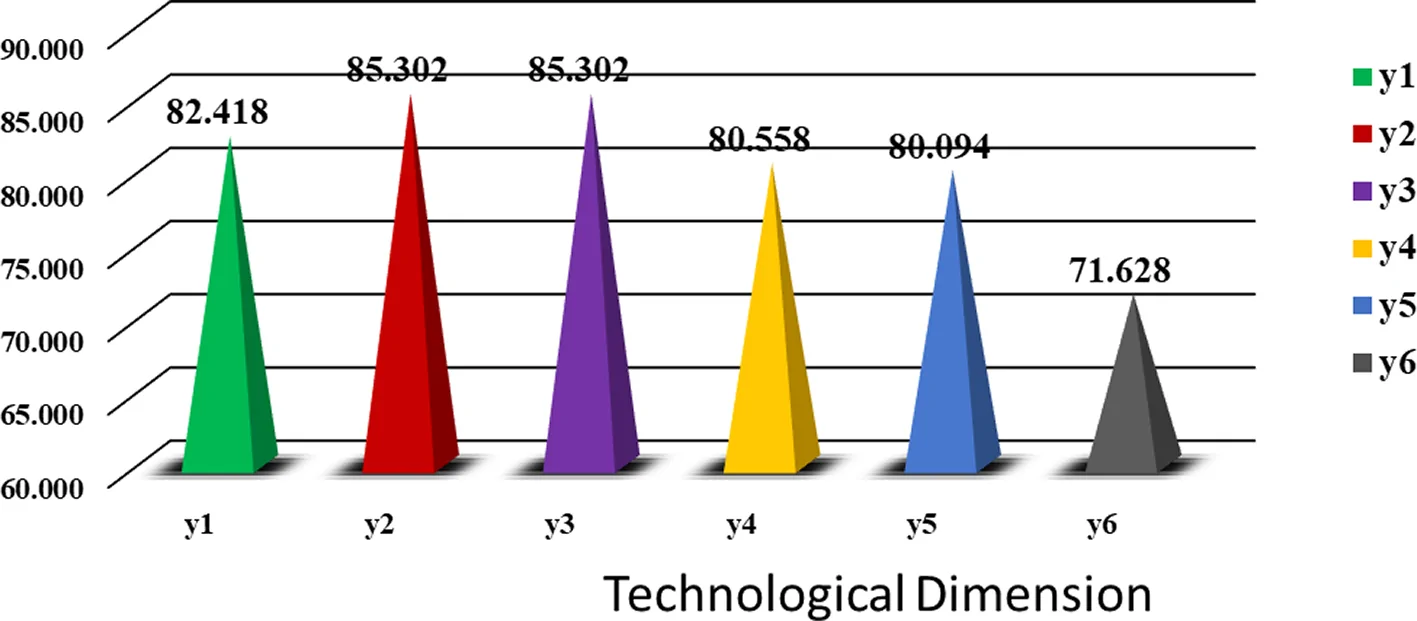
Explains illustrates the relative importance of each item in the technological dimension.

**Table 9.** Frequency distributions, arithmetic averages, standard deviations, and relative importance of the ethical dimension.

Paragraphs	Response										Average	Standard deviation	Response intensity %	Ranking
	Totally agree (5)		Agree (4)		Neutral (3)		I don't agree (2)		Don't totally agree (1)					
	Number	%	Number	%	Number	%	Number	%	Number	%				
**y7**	24	11.2	86	40	77	35.7	24	11.2	4	1.9	3.474	0.901	69.488	6
**y8**	28	13	95	44.2	64	29.8	26	12.1	2	0.9	3.563	0.899	71.256	4
**y9**	26	12.1	90	41.9	67	31.1	26	12.1	6	2.8	3.484	0.951	69.674	5
**y10**	63	29.3	114	53	29	13.5	7	3.3	2	0.9	4.065	0.800	81.302	1
**y11**	47	21.8	116	54	43	20	7	3.3	2	0.9	3.926	0.794	78.512	2
**y12**	40	18.6	122	56.7	43	20	9	4.2	1	0.5	3.89	0.77	77.768	3
**Overall rate**		17.67		48.30		25.02		7.70		1.32	3.733	0.852	74.667	
**Total**	65.97				25.02		9.02							

**Figure 9.**
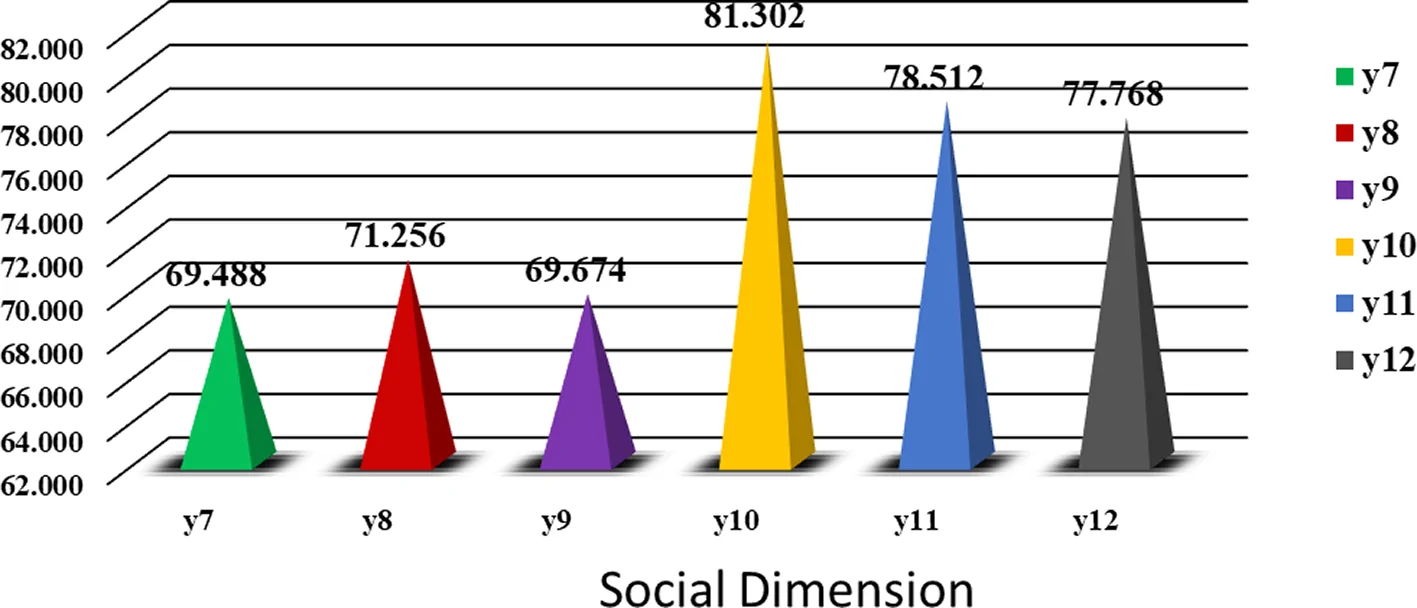
Explains illustrates the relative importance of each item in the moral dimension.

**Table 10.** Frequency distributions, arithmetic averages, standard deviations, and relative importance of the social dimension.

Paragraphs	Response										Average	Standard deviation	Response intensity %	Ranking
	Totally agree (5)		Agree (4)		Neutral (3)		I don't agree (2)		Don't totally agree (1)					
	Number	%	Number	%	Number	%	Number	%	Number	%				
**y13**	51	23.7	109	50.7	39	18.2	11	5.1	5	2.3	3.884	0.907	77.674	6
**y14**	68	31.6	110	51.2	25	11.6	8	3.7	4	1.9	4.070	0.865	81.396	3
**y15**	63	29.3	120	55.8	23	10.7	4	1.9	5	2.3	4.079	0.825	81.582	2
**y16**	69	32.1	121	56.3	20	9.3	3	1.4	2	0.9	4.172	0.725	83.442	1
**y17**	47	21.9	120	55.8	39	18.1	7	3.3	2	0.9	3.944	0.783	78.884	5
**y18**	48	22.3	119	55.3	40	18.6	7	3.3	1	0.5	3.96	0.76	79.162	4
**Overall rate**		26.82		54.18		14.42		3.12		1.47	4.018	0.811	80.357	
**Total**	81.00				14.42		4.58							

**Figure 10.**
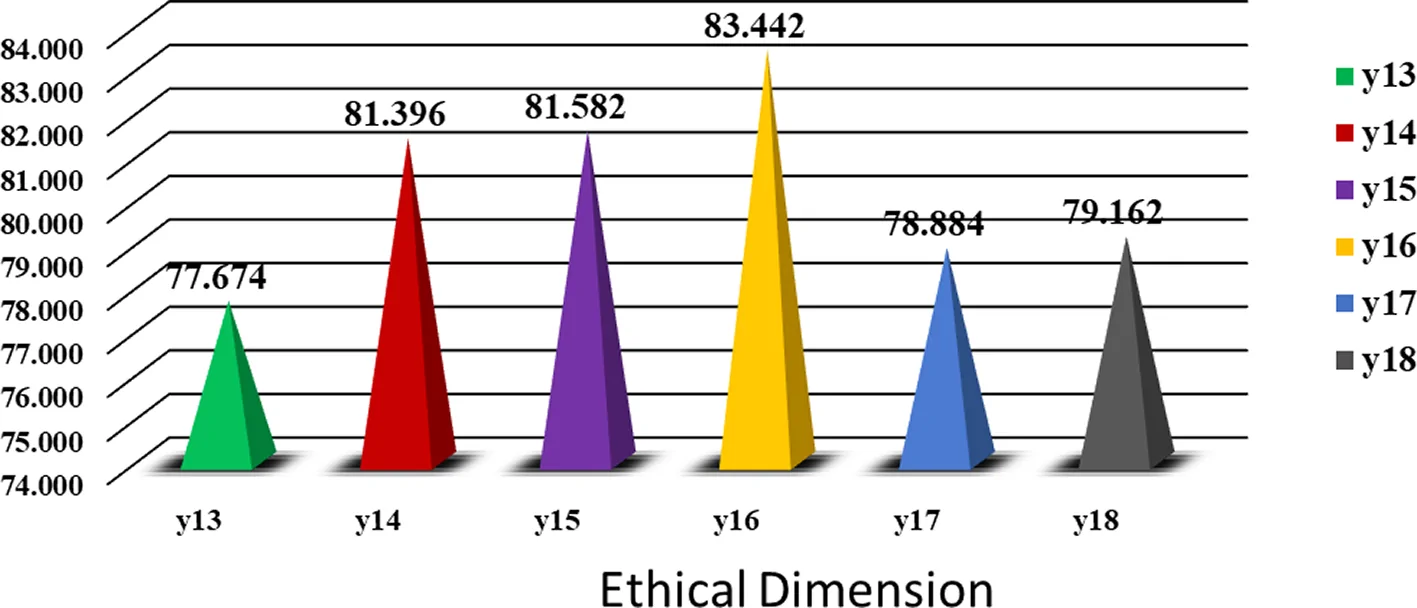
Explains illustrates the relative importance of each item in the social dimension.

**Table 11.** Frequency distributions, arithmetic means, standard deviations, and relative importance of the health dimension.

Paragraphs	Response										Average	Standard deviation	Response intensity %	Ranking
	Totally agree (5)		Agree (4)		Neutral (3)		I don't agree (2)		Don't totally agree (1)					
	Number	%	Number	%	Number	%	Number	%	Number	%				
**y19**	46	21.4	119	55.3	37	17.2	10	4.7	3	1.4	3.907	0.832	78.140	6
**y20**	68	31.6	110	51.2	29	13.4	7	3.3	1	0.5	4.102	0.785	82.046	4
**y21**	57	26.4	127	59.1	26	12.1	4	1.9	1	0.5	4.093	0.704	81.860	5
**y22**	74	34.4	113	52.6	23	10.7	5	2.3	0	0	4.191	0.714	83.814	3
**y23**	89	41.3	89	41.4	30	14	7	3.3	0	0	4.209	0.802	84.186	2
**y24**	91	42.3	95	44.2	21	9.8	8	3.7	0	0	4.25	0.78	85.024	1
**Overall rate**		32.90		50.63		12.87		3.20		0.40	4.126	0.770	82.512	
**Total**	83.53				12.87		3.60							

**Figure 11.**
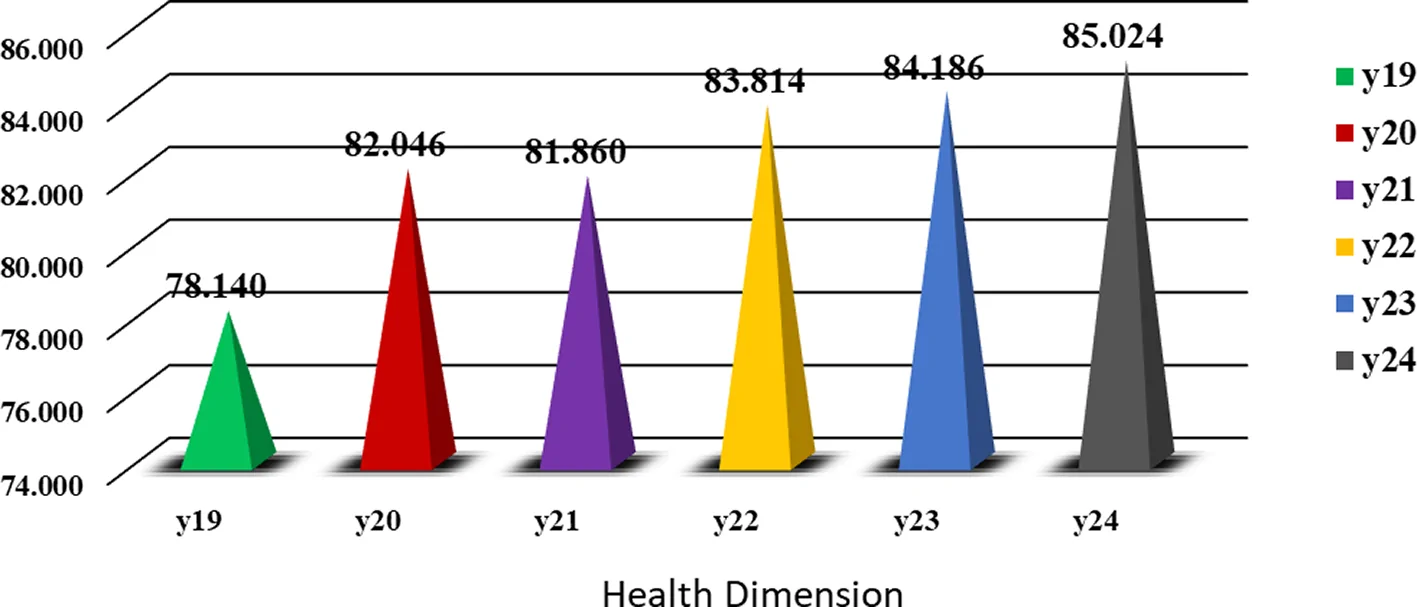
Explains illustrates the relative importance of each item in the health dimension.

**Table 12.** Frequency distributions, arithmetic averages, standard deviations, and the relative importance of the security dimension.

Paragraphs	Response										Average	Standard deviation	Response intensity %	Ranking
	Totally agree (5)		Agree (4)		Neutral (3)		I don't agree (2)		Don't totally agree (1)					
	Number	%	Number	%	Number	%	Number	%	Number	%				
**y25**	59	27.4	117	54.4	30	14	6	2.8	3	1.4	4.037	0.808	80.744	2
**y26**	58	27	122	56.7	32	14.9	2	0.9	1	0.5	4.088	0.702	81.768	1
**y27**	42	19.5	118	54.9	46	21.4	6	2.8	3	1.4	3.884	0.797	77.674	3
**y28**	43	20	111	51.6	52	24.2	5	2.3	4	1.9	3.856	0.827	77.116	4
**y29**	38	17.7	118	54.9	43	20	14	6.5	2	0.9	3.819	0.831	76.372	5
**y30**	30	14	111	51.6	45	20.9	20	9.3	9	4.2	3.62	0.98	72.372	6
**Overall rate**		20.93		54.02		19.23		4.10		1.72	3.884	0.824	77.674	
**Total**	74.95				19.23		5.82							

**Figure 12.**
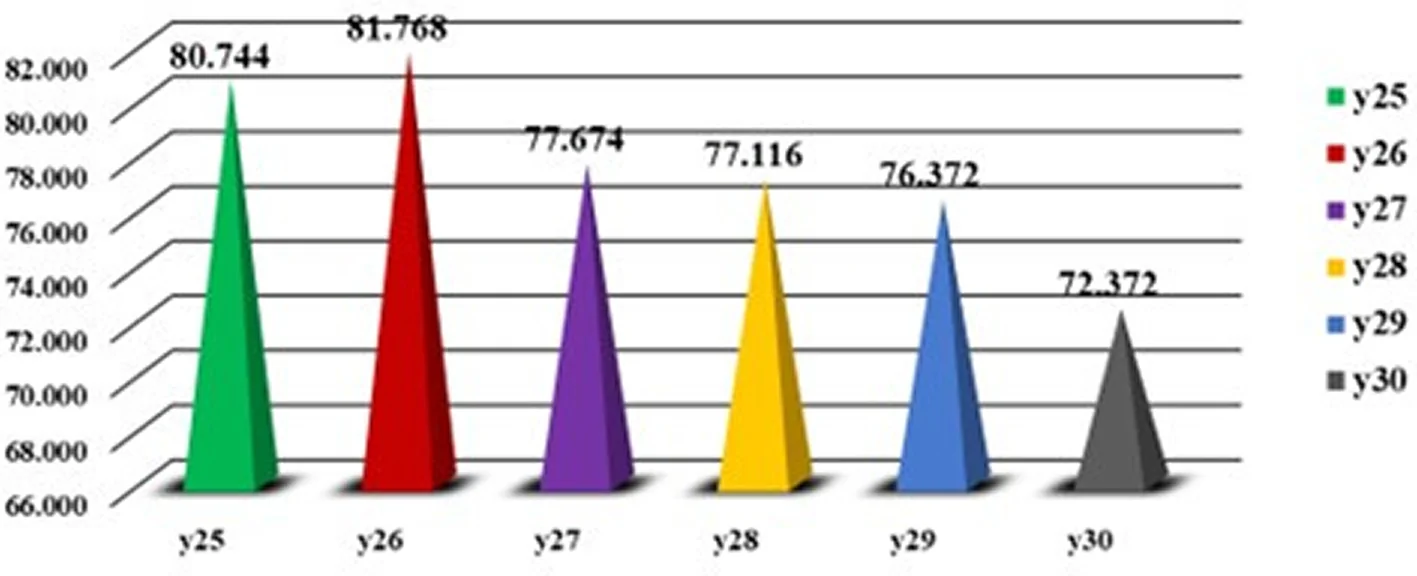
Relative importance of the security dimension.

### Relative importance

From Table 13 above, it is noted that the digital etiquette variable received the highest comparative relative importance, which amounted to (79,200%), while the social marketing variable received relative importance amounting to (77,140%) for the sample studied.

**Table 13.** The relative importance of the study variables.

*T*	*Dimensions*	*Arithmetic mean*	*Standard deviation*	*Relative importance%*	*Ranking*
***1***	***Social marketing***	***3.857***	***0.436***	***77.140***	***2***
***2***	***Digital etiquette***	***3.960***	***0.455***	***79.200***	***1***

### Fifth: Measuring the stability of the questionnaire (Reliability test)

Feldt and Brennan (1989) classified the values of the reliability test into two levels. If the values are greater than (70%), they are considered high-level values, while the values that are less than (70%) are considered low-level values.

The stratified alpha coefficient was found as shown in Table 14 below:

**Table 14.** Measuring the stability of the study variables.

*key variables*	*Alpha stratified coefficient for combined dimensions*
***Alpha stratified coefficient for combined dimensions*** α _st._	***0.94***

It is noted that the value of the stratified alpha coefficient reached 0.94, which is greater than 0.70% for the study variables. Thus, it can be said, through the stratified alpha coefficient, that there is a stability force for the study variables.

### Internal consistency of study dimensions

From Table 15, it is noted that the correlation coefficient values for the social marketing variable appeared between (0.576-0.721), which is greater than (0.3) ( Wu, M. et al., 2016). It is also noted that the correlation coefficient values for the digital etiquette variable appeared between (0.504-0.813), which is also greater than (0.3), which indicates that there is a moral correlation between the paragraphs and the dimensions they represent.

**Table 15.** Internal consistency of variable dimensions.

Internal consistency							
Variables	sub-dimensions	Paragraph sequence					
		First	Second	Third	Fourth	Fifth	Sixth
		The degree to which the paragraph relates to the dimension					
		(P-value)					
**Social marketing**	**Social product**	0.658	0.665	0.694	0.66	0.606	0.688
		0.000	0.000	0.000	0.000	0.000	0.000
	**Social pricing**	0.656	0.695	0.576	0.701	0.674	0.649
		0.000	0.000	0.000	0.000	0.000	0.000
	**Social promotion**	0.612	0.656	0.703	0.721	0.671	0.651
		0.000	0.000	0.000	0.000	0.000	0.000
	**Social place**	0.599	0.649	0.686	0.59	0.65	0.652
		0.000	0.000	0.000	0.000	0.000	0.000
**Digital etiquette**	**Technological dimension**	0.685	0.644	0.666	0.772	0.746	0.504
		0.000	0.000	0.000	0.000	0.000	0.000
	**Ethical dimension**	0.787	0.79	0.803	0.572	0.66	0.659
		0.000	0.000	0.000	0.000	0.000	0.000
	**Social dimension**	0.678	0.711	0.691	0.615	0.654	0.654
		0.000	0.000	0.000	0.000	0.000	0.000
	**Health dimension**	0.702	0.685	0.646	0.793	0.79	0.801
		0.000	0.000	0.000	0.000	0.000	0.000
	**Security dimension**	0.656	0.677	0.77	0.813	0.8	0.729
		0.000	0.000	0.000	0.000	0.000	0.000

### Common Method Bias (CMB)

One of the most important sources for testing the bias of the common method is (the lack of diversification of the sources from which data is collected, the answer is yes regardless of the question, the questions on the form are characterized by their large number, which in turn leads to the length of the questionnaire form). The (Harman single factor test) test was used in the common method bias test, which indicated ( Bagozzi & Yi, 1991) that if the value of this test is greater than (50%), this is evidence of a bias in the common method, and the value of this test was found according to SPSS software. The presence of the bias problem leads to the absence of strong correlations between variables and thus does not reflect the relationship between variables, but rather may have other reasons, which leads to obtaining misleading results.

The test value of the studied data (%CMB = 25.453), which is less than (50%), thus shows that the data does not suffer from common method bias.

### Natural distribution test

When conducting hypothesis tests, it requires the verification of several hypotheses. The most important of these is to impose the return of variables to the normal probability distribution. The normal distribution test is one of the tests on which determining estimation methods and testing hypotheses are based. Failure to achieve the assumption of a normal probability distribution is a violation. In this case, traditional methods cannot be used as the most feasible method in the process of estimating and testing hypotheses. Rather, the researcher must use alternative methods that do not require the availability of this assumption. A test (Kolmogorov Smirnov) was conducted to verify this hypothesis.

From the results shown in Table 16, it is noted that all dimensions had a P value of less than 0.05, which means that these dimensions do not return to the normal probability distribution. It can also be noted that the p value of the two variables (social marketing, digital etiquette) appeared less than 0.05, which indicates that these variables do not return to the normal probability distribution either.

**Table 16.** Testing the normal distribution of study variables and dimensions.

Kolmogorov-Smirnov				
Type	Description	Statistic	N	P-value
**Dimensions**	**Social product**	**0.138**	**215**	**0.000**
	**Social pricing**	**0.167**	**215**	**0.000**
	**Social promotion**	**0.121**	**215**	**0.000**
	**Social place**	**0.132**	**215**	**0.000**
	**Technological dimension**	**0.136**	**215**	**0.000**
	**Ethical dimension**	**0.108**	**215**	**0.000**
	**Social dimension**	**0.132**	**215**	**0.000**
	**Health dimension**	**0.129**	**215**	**0.000**
	**Security dimension**	**0.129**	**215**	**0.000**
**Variables**	**Social marketing**	**0.079**	**215**	**0.002**
	**Digital etiquette**	**0.075**	**215**	**0.005**

Figures 13 & 14 explains the non-return of the two main variables (social marketing and digital etiquette).

**Figure 13.**
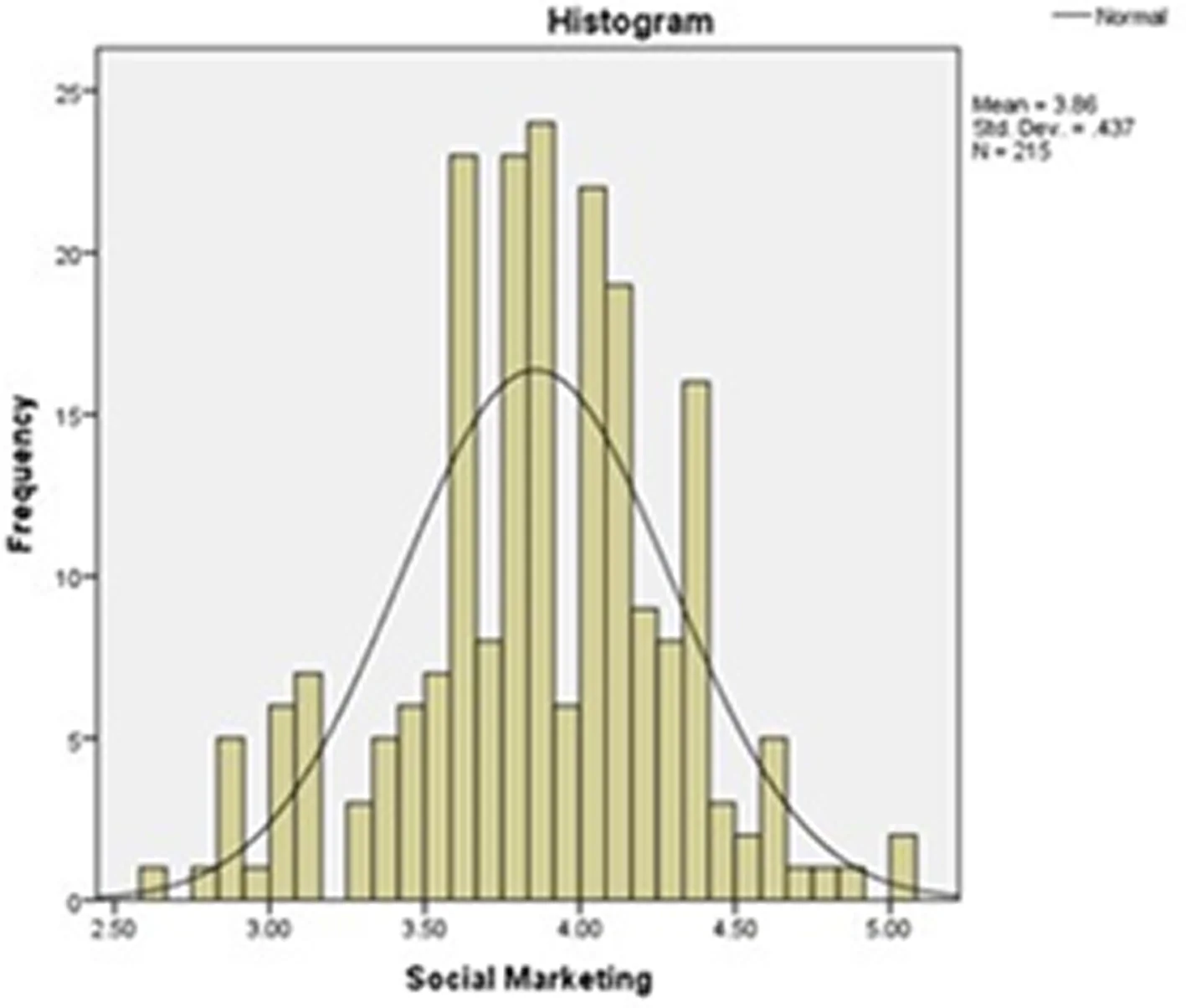
Distribution of the social marketing variable.

**Figure 14.**
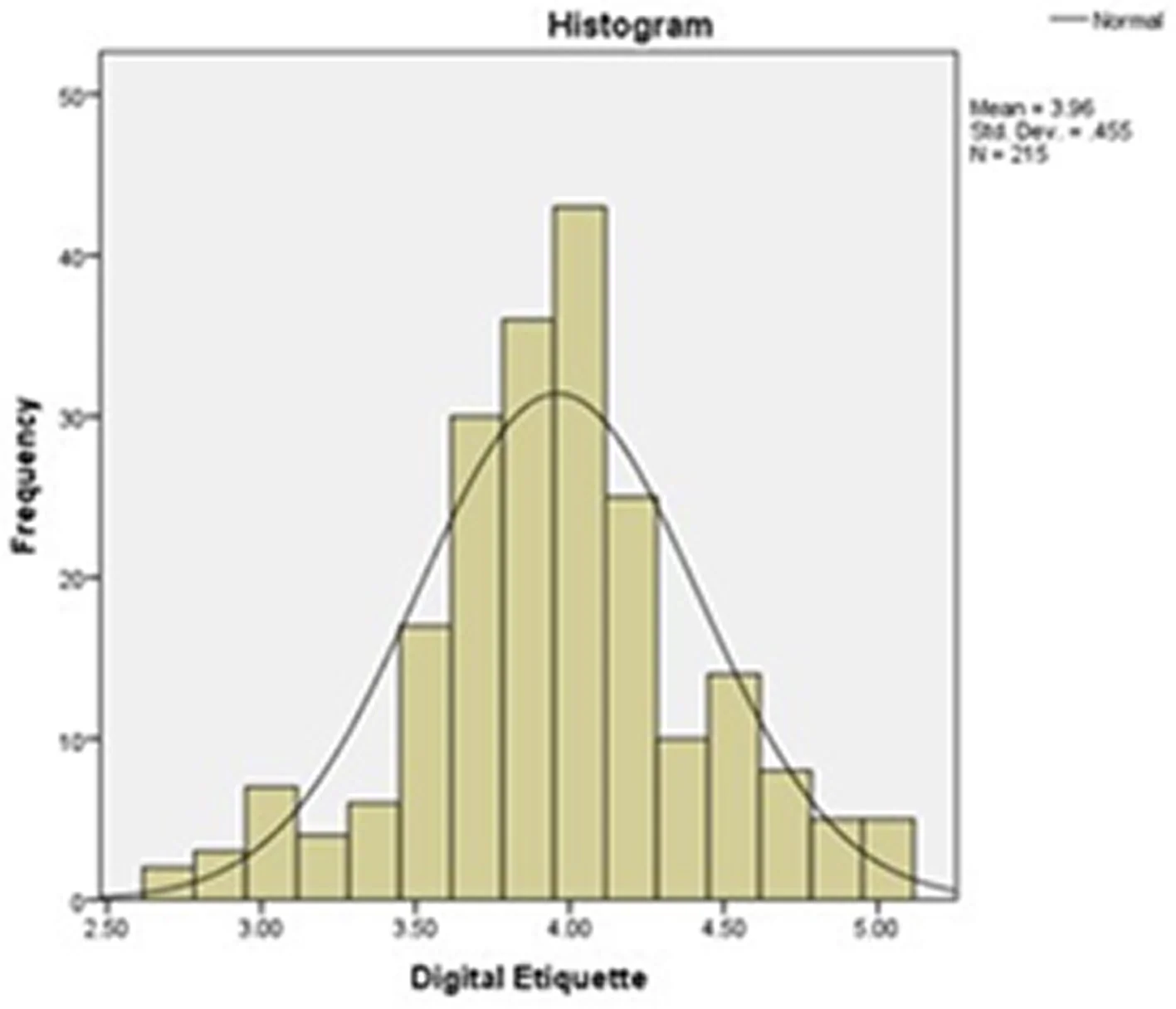
Distribution of the digital etiquette variable.

### Confirmatory factor analysis

The aim of conducting a confirmatory factor analysis of the study variables is to know the correlation relationships between the observations (questions) and the dimension they represent, in addition to knowing the correlation relationships between the latent variables (dimensions) of the variables studied, and thus judging the suitability of the model placed by the researcher with the sample model.

Figure 15 shows the saturation values (Factor Loading) (correlation) of the observed variables (questions) with the indicative (latent) variables related to the study variables, the values of which are shown on the single-headed arrow between the question and the latent variable, as well as the values of the correlation coefficients between each pair of latent variables, the values of which are shown on the double-headed arrow.

**Figure 15.**
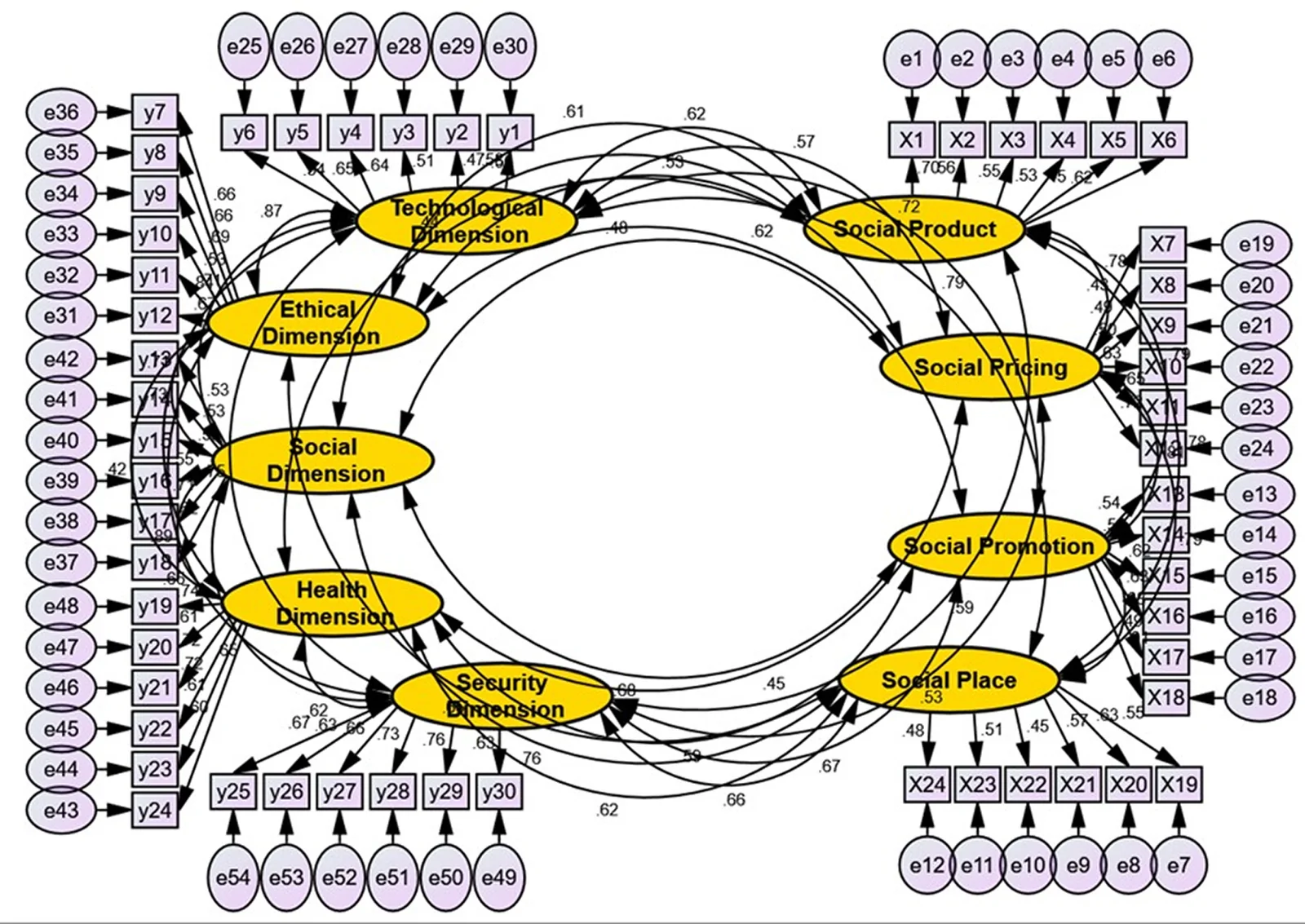
Confirmatory factor analysis of the study variables.

The hypothetical diagram shown in the methodology section determines the significance of the model proposed by the researcher and its conformity with the study sample model. Where conformity quality indicators are verified, which are based on the acceptance limits used by most researchers ( McDonald RP, & MHR, 2002). Through matching quality indicators, it is noted that all indicators are within their acceptable limits, as shown in Table 17 below:

**Table 17.** Conformity quality indicators for confirmatory factor analysis.

Indicators	Value	Result
CMIN/DF	0.60	Identical
GFI	0.93	Identical
AGFI	0.92	Identical
PGFI	0.84	Identical
NFI	0.91	Identical
RFI	0.91	Identical
RMR	0.050	Identical

Standard regression coefficient values

Hair et al. (2016) established saturation values determined by sample size. Through Table 18, which shows the values of the standard regression coefficients (SRW-saturation) in addition to the probability values (P-value) accompanying them. All paragraphs appeared to be significant because the probability values (P-value) accompanying them were less than (0.05). As for the saturation values (SRW), they all appeared greater than (0.40).

**Table 18.** Standard regression coefficient values for confirmatory factor analysis results.

Parameter			SRW	P-value	Parameter			SRW	P-value
**X1**	**←**	**Social product**	0.695	0.001	**y1**	**←**	**Technological dimension**	0.579	0.002
**X2**	**←**		0.558	0.002	**y2**	**←**		0.471	0.002
**X3**	**←**		0.55	0.002	**y3**	**←**		0.506	0.001
**X4**	**←**		0.532	0.002	**y4**	**←**		0.636	0.003
**X5**	**←**		0.454	0.002	**y5**	**←**		0.646	0.002
**X6**	**←**		0.624	0.001	**y6**	**←**		0.537	0.003
**X7**	**←**	**Social pricing**	0.428	0.003	**y7**	**←**	**Ethical dimension**	0.663	0.002
**X8**	**←**		0.49	0.002	**y8**	**←**		0.662	0.003
**X9**	**←**		0.495	0.002	**y9**	**←**		0.685	0.001
**X10**	**←**		0.627	0.003	**y10**	**←**		0.532	0.002
**X11**	**←**		0.653	0.002	**y11**	**←**		0.714	0.001
**X12**	**←**		0.716	0.002	**y12**	**←**		0.665	0.003
**X13**	**←**	**Social promotion**	0.54	0.001	**y13**	**←**	**Social dimension**	0.531	0.003
**X14**	**←**		0.518	0.003	**y14**	**←**		0.533	0.002
**X15**	**←**		0.624	0.004	**y15**	**←**		0.528	0.002
**X16**	**←**		0.625	0.003	**y16**	**←**		0.548	0.002
**X17**	**←**		0.675	0.003	**y17**	**←**		0.711	0.002
**X18**	**←**		0.486	0.004	**y18**	**←**		0.622	0.003
**X19**	**←**	**Social place**	0.549	0.002	**y19**	**←**	**Health dimension**	0.736	0.002
**X20**	**←**		0.627	0.002	**y20**	**←**		0.607	0.001
**X21**	**←**		0.57	0.005	**y21**	**←**		0.725	0.002
**X22**	**←**		0.45	0.003	**y22**	**←**		0.724	0.002
**X23**	**←**		0.508	0.002	**y23**	**←**		0.606	0.004
**X24**	**←**		0.482	0.003	**y24**	**←**		0.603	0.003
					**y25**	**←**	**Security dimension**	0.672	0.002
					**y26**	**←**		0.631	0.002
					**y27**	**←**		0.662	0.004
					**y28**	**←**		0.727	0.002
					**y29**	**←**		0.755	0.004
					**y30**	**←**		0.633	0.003

## Testing study hypotheses

### Analysis of impact hypotheses

Testing impact hypotheses is equivalent to testing correlation hypotheses in terms of importance, so impact hypotheses will be analyzed through regression analysis. Regression analysis determines the amount and direction of the effect of an independent variable on another dependent variable

**Second main hypothesis: There is a statistically significant moral impact of social marketing practices on digital etiquette**. Table 19 below shows the results of testing the fourth main hypothesis:

**Table 19.** Impact results of the social marketing variable in digital etiquette.

Independent variable	Direction of impact	Dependent variable	Regression coefficient Estimate(β)	Standard error of the regression coefficient Se.(β)	95% Confidence Interval		P-value	Result
**Social marketing**	**→**	**Digital etiquette**	1.081	0.317	0.695	1.947	0.002	Acceptance

It is noted that the effect of the social marketing variable on the digital etiquette variable is a direct effect through the positive sign of the regression coefficient Estimate (β), which is due to the social marketing variable, which has a value of (1.081), and the standard error value of the regression coefficient is (0.317). Also, both the lower (Lower) and upper (Upper) confidence limits (95%Confidence Interval), which were worth (1.947, 0.695), were similar in signals at a significant level (0.05). It is also noted that this effect is a morally significant effect through the P value of (0.002), which is less than (0.05), which means that there is a moral effect of the social marketing variable on the digital etiquette variable. Moreover, the R ^2^ value was found to be (0.64), indicating that 64% of the variance in digital ethics is explained by the changes in social marketing. Regarding the effect size, it reached (0.801), representing the Standardized Regression Weights.

Figure 16 explains the effect of the social marketing variable on the digital etiquette variable.

**Figure 16.**
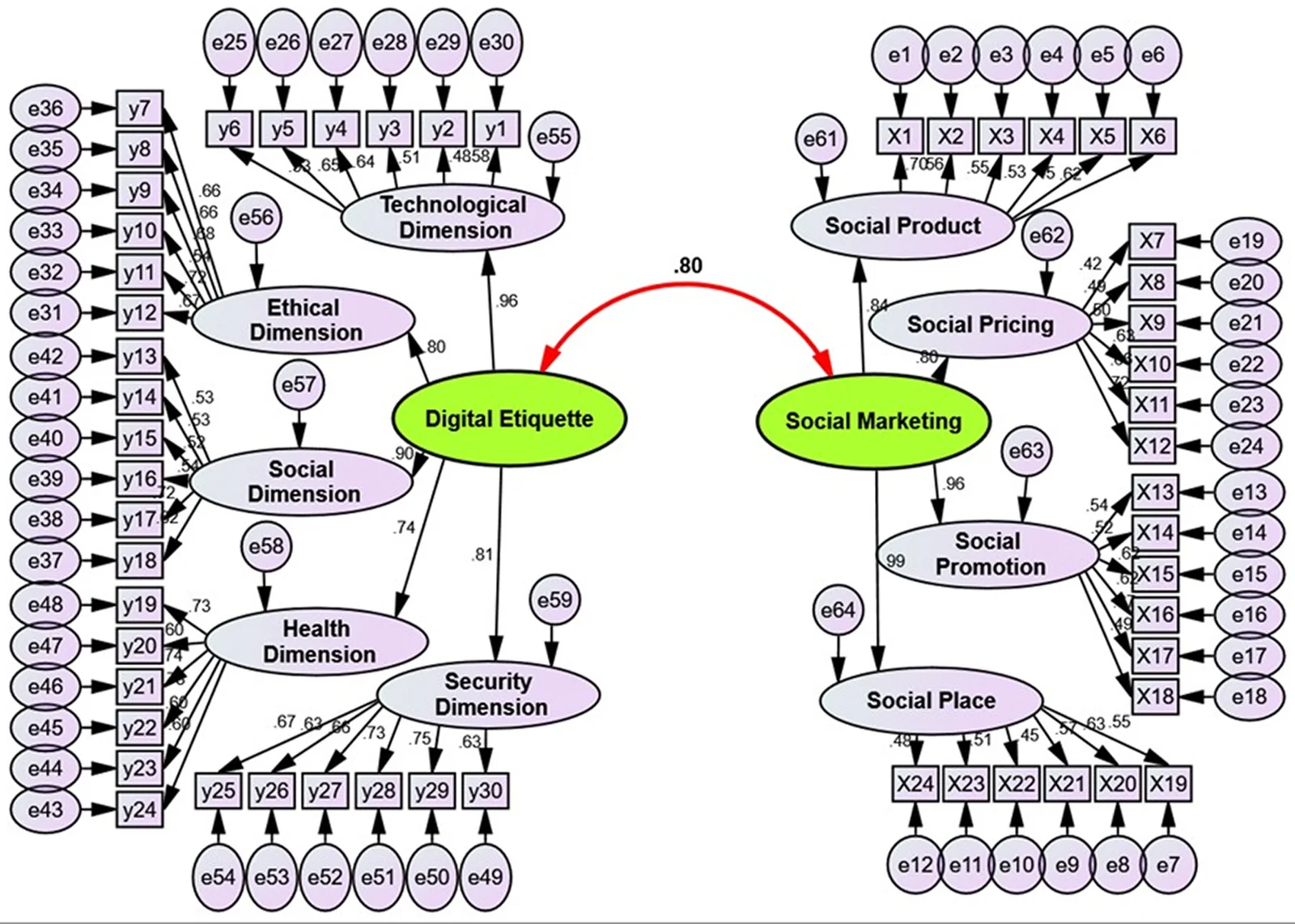
Effect of social marketing on digital etiquette.

The following sub-hypotheses emerge from the fourth main hypothesis:

**The first sub-hypothesis:** There is a statistically significant moral impact of the social product on the dimensions of digital etiquette (technological, ethical, social, health, security).

**The second sub-hypothesis:** There is a statistically significant moral impact of social pricing on the dimensions of digital etiquette (technological, ethical, social, health, security).

**Sub-hypothesis 3**: There is a statistically significant moral impact of social promotion on the dimensions of digital etiquette (technological, ethical, social, health, security).

**Fourth sub-hypothesis:** There is a statistically significant moral impact of social distribution on the dimensions of digital etiquette (technological, ethical, social, health, security).

Table 20 below shows the results of testing the sub-hypotheses above:

**Table 20.** Impact results for the dimensions of the social marketing variable in the digital etiquette variable.

Independent variable	Direction of impact	Dependent variable	Regression coefficient Estimate(β)	Standard error of the regression coefficient Se.(β)	95% Confidence Interval		Probability value P-value	Result
**Social product**	**→**	**Digital etiquette**	0.522	0.132	0.307	0.809	0.003	acceptance
**Social pricing**	**→**		0.690	0.274	0.367	1.366	0.003	acceptance
**Social promotion**	**→**		0.764	0.192	0.479	1.198	0.003	acceptance
**Social place**	**→**		0.796	0.178	0.530	1.261	0.002	acceptance
***R* ^2^**	**0.64**							

It is noted that the effect of the social product dimension on the digital etiquette variable is a direct effect through the positive sign of the regression coefficient Estimate (β), which is due to the social product dimension, which has a value of (0.522), and the standard error value of the regression coefficient is (0.132). Also, both the lower (Lower) and upper (Upper) confidence limits (95% Confidence Interval), which were worth (0.809, 0.307), were similar in signals at a significant level (0.05). It is also noted that this effect is a morally significant effect through the P value of (0.003), which is less than (0.05), which means that there is a moral effect of the social product dimension in the digital etiquette variable. The effect size value was (0.668), indicating the standardized regression weight of the relationship between the variables.

It is also noted that the effect of the social pricing dimension on the digital etiquette variable is a direct effect through the positive sign of the regression coefficient Estimate (β), which is due to the social pricing dimension, which has a value of (0.690), and the standard error value of the regression coefficient is (0.274). Also, both the lower (Lower) and upper (Upper) confidence limits (95% Confidence Interval), whose values were (1.366, 0.367), were similar in signals at a significant level (0.05). It is also noted that this effect is a morally significant effect through the P value of (0.003), which is less than (0.05), which means that there is a moral effect of the social pricing dimension in the digital etiquette variable. The effect size value was (0.612), indicating the standardized regression weight of the relationship between the variables.

It is also noted that the effect of the social promotion dimension on the digital etiquette variable is a direct effect through the positive sign of the regression coefficient Estimate (β), which is due to the social promotion dimension, which has a value of (0.764), and the standard error value of the regression coefficient is (0.192). Also, both the lower (Lower) and upper (Upper) confidence limits (95% Confidence Interval), which had a value of (1.198, 0.479), were similar in signals at a significant level (0.05). It is also noted that this effect is a morally significant effect through the P value of (0.003), which is less than (0.05), which means that there is a moral effect of the social promotion dimension in the digital etiquette variable. The effect size value was (0.718), indicating the standardized regression weight of the relationship between the variables.

Finally, it is noted that the effect of the social place dimension on the digital etiquette variable is a direct effect through the positive sign of the regression coefficient Estimate (β), which is due to the social place dimension, which has a value of (0.796), and the standard error value of the regression coefficient is (0.178). Also, both the lower (Lower) and upper (Upper) confidence limits (95% Confidence Interval), which were worth (1.261, 0.530), were similar in signals at a significant level (0.05). It is also noted that this effect is a morally significant effect through the P value of (0.002), which is less than (0.05), which means that there is a moral effect of the social place dimension in the digital etiquette variable. Moreover, the R ^2^ value was found to be (0.64), indicating that 64% of the variance in digital ethics is explained by social marketing. The effect size value was (0.829), as represented by the Standardized Regression Weight (SRW), reflecting the strength of the relationship between social marketing and digital ethics.

Figure 17 explains the effect of the dimensions of the social marketing variable on the digital etiquette variable.

**Figure 17.**
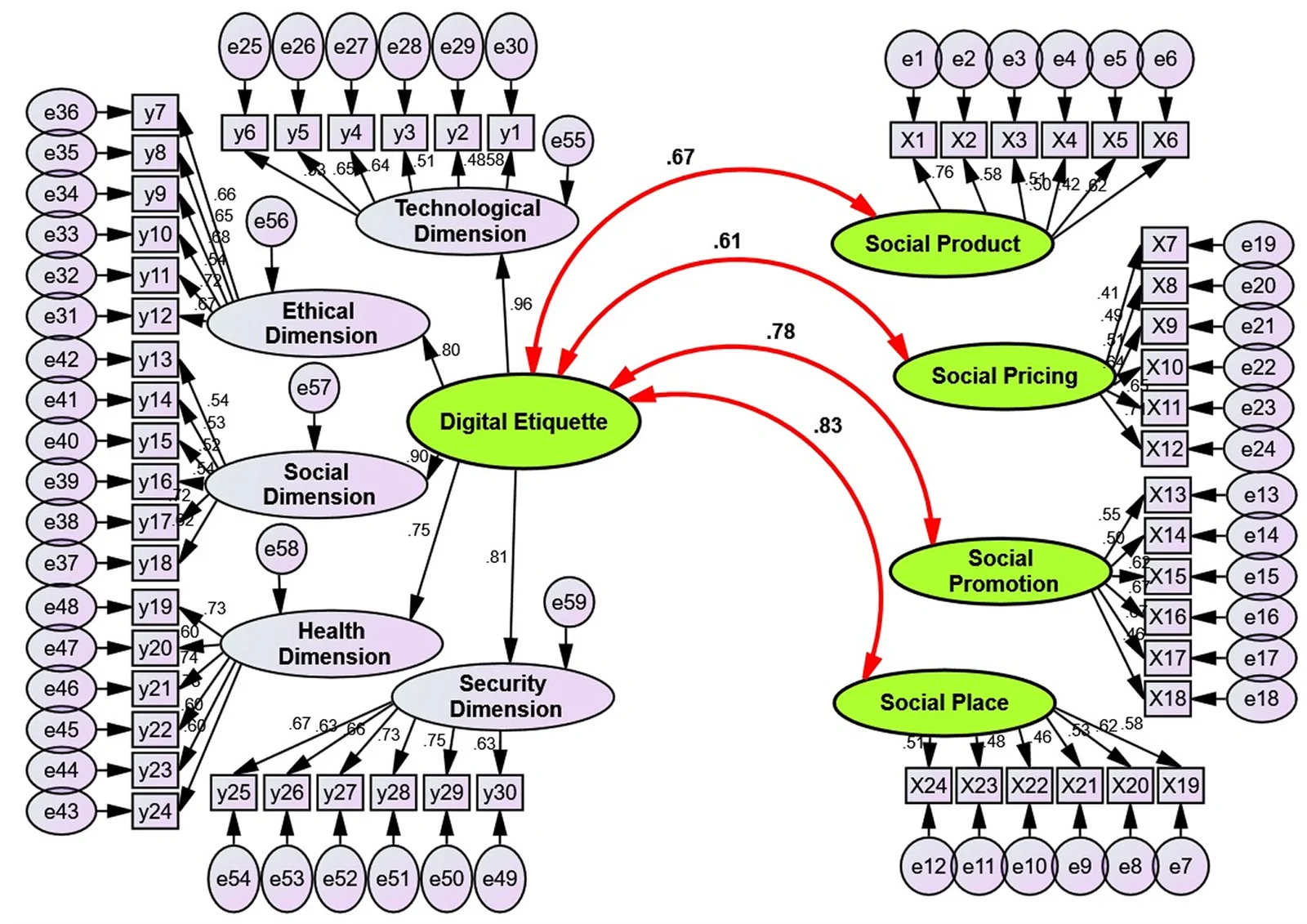
Effect of social marketing dimensions on digital etiquette.

## Conclusions

1. The theoretical contribution of the present study lies in extending the scope of social marketing applications beyond its traditional domains, such as health and environmental contexts, toward the digital and educational domains. By examining its role in explaining and enhancing digital etiquette among university students, this study contributes to the growing body of literature on digital behavior and provides a theoretical foundation for future research in this area.
2. The findings highlight the importance of universities adopting social marketing programs aimed at promoting awareness of responsible digital etiquette. Integrating digital etiquette topics into student activities, educational initiatives, and training programs, while effectively utilizing social media platforms for awareness campaigns, can contribute to strengthening the digital university environment.
3. The comparison of the relative importance of the study variables revealed that digital etiquette demonstrated a higher relative importance compared with social marketing, indicating its greater perceived significance among the study participants.
4. The reliability assessment indicated that the stratified Cronbach’s alpha coefficient reached (0.94), representing a high level of internal consistency. This result confirms the reliability of the research instrument and enhances confidence in the validity of the derived findings.
5. The results of the common method bias assessment revealed no significant bias in the collected data, which strengthens the credibility of the identified relationships among the study variables.
6. The normality test results indicated that both social marketing and digital etiquette variables did not follow a normal distribution. Accordingly, statistical techniques appropriate to the nature and characteristics of the data were employed to ensure the accuracy and robustness of the analysis.
7. The confirmatory factor analysis results demonstrated that the measurement model achieved acceptable goodness-of-fit indices while retaining all questionnaire items. This finding supports the construct validity of the measurement instrument and confirms its suitability for assessing the study variables.
8. The structural analysis revealed a statistically significant effect of social marketing on digital etiquette among the study participants, providing empirical evidence supporting the relationship between the two constructs within the boundaries of the research design.
9. Furthermore, the findings demonstrated that all dimensions of social marketing exerted statistically significant effects on digital etiquette, emphasizing the importance of adopting an integrated approach when designing programs and initiatives aimed at promoting responsible digital behavior.

## Discussion

The findings indicate that social marketing practices have contributed significantly to enhancing digital etiquette among students at the University of Fallujah. This result can be explained by the fact that social marketing strategies extend beyond merely disseminating information; rather, they seek to influence individuals’ attitudes, motivations, and behavioral intentions. This interpretation is consistent with the assumptions of the Theory of Planned Behavior, which suggests that strengthening positive attitudes and perceived social norms increases the likelihood of adopting desirable behaviors. Furthermore, the findings support Social Learning Theory, which emphasizes that individuals acquire behaviors through observation, interaction, and engagement with their surrounding social environment.

The emergence of the social distribution dimension as the strongest predictor of digital etiquette can be attributed to the role of accessibility in facilitating responsible digital behavior adoption. Easy access to digital services, educational platforms, and awareness materials reduces barriers that may hinder students from engaging in appropriate digital practices. When awareness messages, digital applications, and educational resources are continuously available and easy to use, students become more exposed to positive digital practices, which subsequently influences their daily behaviors within the university environment. This finding is consistent with the social marketing literature, which considers accessibility and convenience as critical determinants of successful behavioral change interventions, as they reduce the effort, time, and perceived costs associated with adopting the desired behavior.

The findings are also consistent with the results of previous studies, including Arenas-Escaso et al. (2024) and Saud (2022), which emphasized that promotional activities and awareness campaigns contribute to strengthening responsible online behavior by increasing awareness and encouraging positive digital practices. Similarly, the current study demonstrates that social promotion represents one of the most influential dimensions of social marketing in enhancing digital etiquette among university students.

Moreover, the study revealed a relatively high level of student commitment to the technological and health-related dimensions of digital etiquette. This finding aligns with the concerns highlighted by the World Health Organization (2025) regarding the importance of increasing awareness of health risks associated with excessive technology use. Such agreement reflects students’ readiness to respond to awareness initiatives when these initiatives are implemented within a supportive university environment that encourages responsible technology use.

However, these findings differ from some previous studies that argued that awareness campaigns alone may not be sufficient to achieve sustainable behavioral change unless they are supported by appropriate technological infrastructure and clear institutional policies that reinforce responsible digital practices. This discrepancy may be attributed to differences in the Iraqi university context, the level of available digital services, variations in sample characteristics, and differences in the degree of digital transformation across educational institutions where previous studies were conducted.

From a practical perspective, the findings suggest that universities should consider social marketing as an integrated managerial strategy rather than viewing it merely as a temporary communication campaign. Enhancing access to digital services, developing continuous awareness messages, and providing user-friendly technological environments are essential actions that can contribute to improving students’ adherence to digital etiquette. Such initiatives can support the quality of digital education and reduce undesirable digital behaviors.

Theoretically, these findings provide further support for the fundamental assumptions of the Theory of Planned Behavior, which explains that behavioral adoption is influenced by attitudes, perceived social norms, and perceived behavioral control. Additionally, the findings are consistent with Social Learning Theory by demonstrating that educational environments and awareness messages serve as important sources for acquiring positive digital behaviors through observation, social interaction, and continuous engagement.

## Recommendations

Based on the results of the study and analysis of digital reality at the University of Fallujah, the researchers recommend the following:

1. Enhancing the adoption by Iraqi universities of social marketing practices that contribute to enhancing digital etiquette among students, by employing innovative methods in spreading digital behavioral awareness, such as the use of multimedia, awareness videos, and infographics, in a way that enhances the delivery of ethical messages to target groups more effectively.
2. Strengthening partnership between academic leaders (deanships of colleges, scientific departments, electronic media division) in consolidating digital values and responsibility.
3. Including mandatory training programs for new students that address digital etiquette, cyber security, and digital identity management, ensuring early awareness building among students about how to use educational platforms and digital technologies in a safe and ethical manner.
4. Investing in developing technological infrastructure and modernizing digital systems and platforms within universities, ensuring improved quality of digital interaction and enhancing a responsible behavioral environment.
5. Building human resources capabilities by training academic and administrative cadres on digital awareness methods, to ensure an effective and sustainable impact on student behavior.
6. Develop periodic monitoring and evaluation systems to measure the impact of social marketing practices on improving digital behavior, and identify the strengths and weaknesses of awareness programs, which helps improve them and direct them towards students’ needs.
7. Expanding the study of other variables affecting digital behavior, such as digital well-being, technical culture, institutional and governmental support, and psychological and social factors, to allow for a deeper understanding of digital behavior in the university environment.
8. Conducting similar studies in other Iraqi universities for the purpose of comparison, which contributes to developing unified educational policies that promote ethical digital behavior at the national level.

## Future research

Future research could extend the current study by adopting longitudinal or experimental research designs to provide deeper insights into the causal mechanisms underlying the relationship between social marketing practices and digital etiquette. Such approaches would help examine how changes in social marketing interventions over time influence students’ digital behaviors and provide stronger evidence regarding the directionality of these relationships.

Furthermore, future studies are encouraged to expand the research context by including students from different public and private universities across various geographical regions to enhance the generalizability of the findings. Comparative studies across different educational and cultural contexts may also provide valuable insights into how institutional and environmental factors shape the adoption of responsible digital behaviors.

In addition, future research could investigate potential mediating and moderating variables that may explain or strengthen the relationship between social marketing and digital etiquette. Variables such as digital citizenship, digital literacy, digital intelligence, perceived behavioral control, and technology acceptance may provide a more comprehensive understanding of the mechanisms through which social marketing contributes to promoting responsible digital practices among university students.

## Ethical considerations

This study involved human participants and was conducted in accordance with accepted ethical research standards and the principles outlined in the Declaration of Helsinki. Ethical approval was obtained from the Scientific Research Ethics Committee, University of Fallujah, Iraq (Approval No. HOF.HUM.2025.001). Written informed consent was obtained from all participants prior to their participation. All participants were informed about the purpose of the study, the voluntary nature of their participation, their right to withdraw at any time without consequences, and the confidentiality of their data.

## Data Availability

The data supporting the findings of this study are openly available in Zenodo at: https://doi.org/10.5281/zenodo.18475239, Hammadi, A. A., & Reham, M. H. (2026). The Impact of Social Marketing Practices in Promoting Digital Etiquette A Descriptive and Analytical Study on a Sample of Fallujah University Students [Data set]. Zenodo These data are available under the terms of the Creative Commons Zero “No rights reserved” data waiver (CC0 4.0 Public Domain Dedication). This study is an observational survey-based research and follows the STROBE reporting guidelines. No CONSORT or ARRIVE checklists are required, as the study does not involve clinical trials or animal experiments.
