# Supplementary material for: The impact of social marketing practices in promoting digital etiquette: a descriptive and analytical study on a sample of Fallujah University students
Source: F1000Res. 2026 Aug 24;15:553. Originally published 2026 Apr 18. [Version 2] doi: 10.12688/f1000research.173990.2 (PMC13631445; doi:10.12688/f1000research.173990.2)
Supplement: Peer review history

# Reviewer report: Reviewer response for version 2

**DOI:** 10.5256/f1000research.205748.r514814

**Published:** 2026-10-01

**Authors:** Ujang Sumarwan

**Affiliations:** 1 IPB University, Bogor, Indonesia

1. The clarity of presentation, accuracy of scientific reporting, and appropriate use of contemporary and relevant references contribute positively to the manuscript’s academic rigor, credibility, and scholarly contribution.

 

1. The research model should be further strengthened by providing a clear and comprehensive explanation of how each latent independent variables and latent dependent variable are  theoretically derived from the underlying theories adopted as the foundation of the study. The authors are encouraged to explicitly establish the theoretical rationale for including each latent variable and explain how its conceptualization and relationships with other variables are supported by relevant theoretical frameworks and previous empirical studies.

 

1. I have reviewed the methodology section and recommend reorganizing it into the following sections and subsections to improve its clarity, coherence, and methodological rigor:

1. Research Design

 2. Research Variables

 3. Operational Definitions and Measurement of Variables

 4. Data Collection Methods

 5. Sampling Method and Selection Procedures

 6. Sampling Unit

 7. Sample Size and Its Justification

 8. Statistical Analysis Methods

 The authors should provide a clear and comprehensive explanation of each subsection to ensure that the research methodology is transparent, systematic, and reproducible. Particular attention should be given to the justification of the sampling method, sample size determination, validity and reliability of the measurement instruments, and appropriateness of the statistical techniques employed.

1. The discussion should shift from a predominantly statistical interpretation toward a more substantive, theory-driven, and marketing-oriented explanation of the research findings. Statistical results should serve as the empirical foundation for the discussion, rather than constitute its primary focus. A stronger emphasis on marketing interpretation will enhance the theoretical contribution, practical relevance, and managerial implications of the study.

 

1. The authors should include a clear Data Availability Statement specifying where and how the underlying research data and supporting materials can be accessed. If public access is restricted due to confidentiality, ethical considerations, or institutional regulations, these limitations should be explicitly explained, together with the conditions under which qualified researchers may request access.

 

1. The conclusions was  clearly and adequately supported by the empirical findings presented in the study. The  conclusion is directly derived from the research results and is consistent with the stated research objectives, research questions, and hypotheses.

Is the work clearly and accurately presented and does it cite the current literature?

Yes

If applicable, is the statistical analysis and its interpretation appropriate?

Partly

Are all the source data underlying the results available to ensure full reproducibility?

Yes

Is the study design appropriate and is the work technically sound?

Partly

Are the conclusions drawn adequately supported by the results?

Yes

Are sufficient details of methods and analysis provided to allow replication by others?

Partly

Reviewer Expertise:

NA

I confirm that I have read this submission and believe that I have an appropriate level of expertise to confirm that it is of an acceptable scientific standard, however I have significant reservations, as outlined above.

# Reviewer report: Reviewer response for version 2

**DOI:** 10.5256/f1000research.205748.r514212

**Published:** 2026-08-25

**Authors:** Rahul Chauhan

**Affiliations:** 1 Karnavati University, Gandhinagar, India

The revised version of the manuscript has been carefully reviewed in light of the comments and recommendations provided in the previous review. The authors have made substantial and appropriate revisions to improve the clarity, coherence, theoretical grounding, and overall academic quality of the manuscript.

 In particular, the literature review and discussion have been strengthened with additional theoretical support and clearer interpretation of the findings. The conclusions have also been refined to ensure better alignment with the empirical results, while the addition of a dedicated Future Research section provides useful directions for subsequent research. The language and presentation of the manuscript have been improved, enhancing its overall readability and consistency.

 Based on my assessment, the major concerns raised in my previous review have been adequately addressed. I appreciate the authors' efforts to respond to the reviewers' recommendations and improve the manuscript.

 Overall, I consider the revised manuscript suitable for approval. I therefore recommend the status: **Approved**.

Is the work clearly and accurately presented and does it cite the current literature?

Partly

If applicable, is the statistical analysis and its interpretation appropriate?

Partly

Are all the source data underlying the results available to ensure full reproducibility?

Yes

Is the study design appropriate and is the work technically sound?

Partly

Are the conclusions drawn adequately supported by the results?

Partly

Are sufficient details of methods and analysis provided to allow replication by others?

Partly

Reviewer Expertise:

Marketing, Social Marketing, Consumer Behaviour, Digital Marketing, Educational Technology, Higher Education Research, Research Methodology, Quantitative Data Analysis, Structural Equation Modeling (SEM), Behavioral Studies, and Management Research.

I confirm that I have read this submission and believe that I have an appropriate level of expertise to confirm that it is of an acceptable scientific standard.

# Reviewer report: Reviewer response for version 1

**DOI:** 10.5256/f1000research.191857.r487188

**Published:** 2026-06-12

**Authors:** Dijana Vukovic

**Affiliations:** 1 Sveučilište Sjever, Varaždin, Croatia

**Reviewer Report**

**Summary of the Article**

 This manuscript investigates the impact of social marketing practices on promoting digital etiquette among students at the University of Fallujah. The study examines four dimensions of social marketing (social product, social pricing, social promotion, and social distribution) and their relationship with digital etiquette, conceptualised through technological, ethical, social, health, and security dimensions. Using a questionnaire administered to 215 students, the authors employ descriptive statistics, reliability analysis, confirmatory factor analysis, correlation analysis, and regression analysis to test the proposed relationships.

 The topic is timely and relevant, particularly given the increasing importance of responsible digital behaviour in higher education environments. The manuscript also benefits from the inclusion of recent literature and addresses a socially important issue. However, several theoretical, methodological, and statistical limitations currently reduce the scientific contribution of the study and should be addressed before indexing.

**Major Comments**

**1. Theoretical Framework and Research Gap**

 Although the manuscript reviews relevant literature on social marketing and digital etiquette, the research gap is not sufficiently articulated. The introduction provides numerous definitions and previous findings but does not clearly explain what remains unknown in the literature and how the present study advances existing knowledge.

 The authors should explicitly identify:

- the specific gap in previous studies,
- the novelty of examining social marketing and digital etiquette together,
- the theoretical contribution of the study.

Furthermore, the conceptual relationship between the dimensions of social marketing and the dimensions of digital etiquette remains largely descriptive. A stronger theoretical rationale explaining why and how social marketing influences digital etiquette would strengthen the manuscript considerably.

**2. Sampling Procedure and Research Design**

 The overall research design is appropriate for addressing the research question. However, important details regarding the sampling procedure are missing.

 The manuscript should provide:

- the total size of the student population,
- the number of distributed questionnaires,
- the response rate,
- a clearer explanation of the sampling strategy.

Without this information, it is difficult to assess the representativeness of the sample and the generalisability of the findings.

**3. Measurement Instrument**

 The questionnaire is insufficiently described. While the manuscript reports the number of items included in each construct, it does not provide enough information regarding:

- the origin of the measurement scales,
- scale adaptation procedures,
- content validity assessment,
- the wording of the questionnaire items.

The inclusion of an appendix containing the complete measurement instrument would substantially improve transparency and reproducibility.

**4. Statistical Analysis**

 The statistical procedures employed are generally appropriate for the research objectives. However, several methodological issues require attention.

 The manuscript reports non-normal data distributions based on the Kolmogorov-Smirnov test yet subsequently applies parametric techniques without sufficient justification. The authors should explain why the selected statistical procedures remain appropriate despite the observed deviations from normality or provide additional robustness analyses.

 The confirmatory factor analysis is not reported according to current methodological standards. To strengthen the validity of the measurement model, the authors should report:

- Composite Reliability (CR),
- Average Variance Extracted (AVE),
- discriminant validity assessment (Fornell-Larcker criterion and/or HTMT ratio),
- complete CFA model fit statistics.

The regression analysis would also benefit from additional reporting, including:

- R² values,
- multicollinearity diagnostics (e.g., VIF),
- effect sizes,
- Assumption testing.

**5. Discussion and Interpretation**

 The discussion section remains largely descriptive and primarily reiterates the statistical findings. Greater emphasis should be placed on interpreting the results in relation to previous research and explaining potential reasons for the observed relationships.

 In particular, the authors should discuss:

- why social distribution emerged as the strongest predictor,
- the practical implications of the findings for universities,
- similarities and differences compared with previous studies.

A deeper theoretical discussion would significantly improve the contribution of the manuscript.

**6. Conclusions**

 The conclusions are generally supported by the reported findings. However, some statements appear broader than the empirical evidence permits. Given the cross-sectional design and single-institution sample, causal interpretations should be avoided, and the conclusions should be framed more cautiously.

 The conclusion section would benefit from:

- a clearer statement of theoretical contributions,
- a discussion of practical implications,
- a dedicated subsection addressing limitations,
- recommendations for future research.

**Reproducibility and Data Availability**

 The authors have made the dataset publicly available through a Zenodo repository, which is a significant strength of the study and supports transparency and reproducibility. However, reproducibility could be further improved by providing a codebook, detailed variable descriptions, and additional documentation of the analytical procedures.

**Points That Must Be Addressed Before Indexing**

 The following issues should be addressed to ensure the scientific soundness of the manuscript:

1. Clearly define the research gap and theoretical contribution.
2. Provide a more detailed description of the sampling procedure and response rate.
3. Expand the description of the measurement instrument and scale development process.
4. Report additional measurement model validity indicators (CR, AVE, discriminant validity).
5. Justify the use of parametric analyses despite reported non-normality.
6. Provide more comprehensive reporting of regression model diagnostics and results.
7. Strengthen the discussion by comparing findings with previous studies and offering a deeper theoretical interpretation.
8. Revise the conclusions to better reflect the limitations of the research design and avoid overly broad claims.

**Recommendation**

**Major Revision**

 The manuscript addresses an important and relevant topic and has the potential to make a useful contribution to the literature. However, substantial revisions are required, particularly in relation to methodological transparency, measurement validation, statistical reporting, and theoretical interpretation, before the manuscript can be considered for indexing

Is the work clearly and accurately presented and does it cite the current literature?

Yes

If applicable, is the statistical analysis and its interpretation appropriate?

Partly

Are all the source data underlying the results available to ensure full reproducibility?

Yes

Is the study design appropriate and is the work technically sound?

Partly

Are the conclusions drawn adequately supported by the results?

Partly

Are sufficient details of methods and analysis provided to allow replication by others?

Partly

Reviewer Expertise:

NA

I confirm that I have read this submission and believe that I have an appropriate level of expertise to confirm that it is of an acceptable scientific standard, however I have significant reservations, as outlined above.

# Response

**Published:** 2026-07-04

**Authors:** Reham Hamza

**Affiliations:** college, University of Fallujah, Al-Fallujah, Al Anbar Governorate, Iraq

We sincerely thank the reviewers for their valuable, constructive, and insightful comments. The suggestions provided have significantly contributed to improving the theoretical foundation, methodological rigor, and overall quality of our manuscript. We have carefully considered all comments and revised the manuscript accordingly. A point-by-point response is presented below.

 Response to Reviewer #3 — Dijana Vukovic

 Comment #1:

 The study lacks a clearly developed theoretical framework explaining how social marketing practices influence digital etiquette. The inclusion of established theories such as the Theory of Planned Behavior, Social Cognitive Theory, Digital Citizenship Theory, or a formal social marketing framework would strengthen the conceptual foundation.

 Response:

 We appreciate this valuable suggestion. We have substantially revised the theoretical framework by incorporating relevant theories that explain the relationship between social marketing practices and digital etiquette. Specifically, we integrated the Theory of Planned Behavior, Social Cognitive Theory, and Digital Citizenship Theory to provide a stronger theoretical explanation of how social marketing mechanisms can influence students’ awareness, attitudes, and responsible digital behaviors.

 Location in revised manuscript: Theoretical Framework Section.

 Comment #2:

 The sampling procedure lacks sufficient clarification. The manuscript does not specify the total population size of Fallujah University students, sampling framework, or response rate.

 Response:

 We agree with the reviewer’s observation. We have added a detailed explanation of the sampling procedure, including the target population, sample size determination mechanism, sampling approach, and response rate. These additions clarify the representativeness of the study sample.

 Location in revised manuscript: Methodology Section.

 Comment #3:

 The statistical analysis requires further justification because the variables did not follow a normal distribution, while parametric tests were applied.

 Response:

 We appreciate this methodological comment. The revised manuscript now provides additional justification for the analytical procedures. Given the non-normal distribution of the variables, we applied robust estimation procedures and clarified the suitability of the adopted analytical approach. The rationale for using the selected statistical techniques has been added to the methodology section.

 Location in revised manuscript: Data Analysis Section.

 Comment #4:

 There are inconsistencies in demographic percentages.

 Response:

 We carefully reviewed all demographic statistics and corrected the reported percentages to ensure complete consistency between the text and tables.

 Location in revised manuscript: Demographic Characteristics Table.

 Comment #5:

 The construct validity analysis should report reliability and validity indicators such as CR, AVE, and discriminant validity.

 Response:

 We appreciate this important suggestion. The revised manuscript includes additional validity assessments and clarifies the measurement model evaluation procedures. Where applicable, reliability, convergent validity, and discriminant validity indicators have been reported and discussed.

 Location in revised manuscript: Measurement Model Assessment Section.

 Comment #6:

 The manuscript should report explanatory power measures such as R², adjusted R², and effect size.

 Response:

 Following the reviewer’s recommendation, we have added R² values, adjusted R² values, and effect size indicators to the hypothesis testing results. These additions provide a clearer understanding of the practical significance and explanatory capability of the proposed model.

 Location in revised manuscript: Results and Hypothesis Testing Sections.

# Reviewer report: Reviewer response for version 1

**DOI:** 10.5256/f1000research.191857.r487194

**Published:** 2026-06-11

**Authors:** Harmon Chaniago

**Affiliations:** 1 Bandung State Polytechnic, Bandung, Indonesia

The manuscript addresses a relevant topic. Digital etiquette is important in higher education. Social marketing is also a suitable perspective for studying behavioural change. The use of recent literature and the inclusion of a public data availability statement are positive elements.

 However, the manuscript does not yet meet the scientific standard expected by a Q1 or top-tier journal. The main weakness lies in the mismatch between the research design and the claims made by the authors. The study uses a cross-sectional self-report survey involving 215 students from one university. This design is useful for identifying relationships between variables. It is not sufficient to prove impact, behavioural change, or causal influence.

 The abstract states that students’ commitment to digital etiquette increased by 25%. This claim is not adequately supported. The study does not include baseline data, a pre-test and post-test design, an intervention, a control group, or longitudinal evidence. For this reason, the title, abstract, hypotheses, results, and conclusion present claims that go beyond what the data can support.

**Major issues that must be addressed**:

1. The manuscript has several serious weaknesses. First, the theoretical contribution is still limited. The paper uses the four dimensions of social marketing, but it does not develop a strong theory-based model. The research gap is not clear. The authors also need to explain why social marketing is the most suitable lens for understanding digital etiquette, compared with other frameworks such as behaviour change or digital citizenship.
2. The literature review is recent, but mostly descriptive. It does not critically compare previous studies. It also does not explain what remains unknown, what findings are inconsistent, and what new contribution this study offers. A Q1 marketing journal requires a stronger conceptual contribution.
3. The sampling procedure also needs major clarification. The authors mention a comprehensive inventory method, but they do not report the population size, sampling frame, response rate, inclusion criteria, exclusion criteria, or non-response handling. Some demographic data are inconsistent. For example, the text reports female respondents as 54%, while the table reports 45%. The age categories are also unclear.
4. The measurement process is not transparent enough. The paper states that social marketing was measured with 24 items and digital etiquette with 30 items. Yet, the full items, scale sources, adaptation process, translation procedure, and content validation are not fully explained. This limits replication.
5. The statistical analysis needs improvement. The data are non-normal, but the authors still use Pearson correlation and regression without strong justification. They should consider robust estimation, bootstrapping, or non-parametric alternatives. They also need to report standardized coefficients, R-squared, adjusted R-squared, effect sizes, VIF, residual diagnostics, and model assumptions.
6. The confirmatory factor analysis is incomplete. Key indicators such as chi-square, degrees of freedom, CFI, TLI, RMSEA, SRMR, CR, AVE, and discriminant validity are missing. Several factor loadings are low. The CMIN/DF value of 0.60 is unusual and should be checked again.
7. The common method bias test is also insufficient. Harman’s single-factor test alone is weak, especially because the study uses one questionnaire, one source, and one time point. The authors should add stronger procedural and statistical checks.
8. The results section is too long and repetitive. Many frequency tables and figures are not necessary. The findings should be reorganized into concise tables for the measurement model, structural model, and robustness tests.
9. The discussion is still general. It repeats the results, but does not explain the mechanisms behind the findings. It also does not discuss alternative explanations, boundary conditions, or study limitations. The authors need to clearly acknowledge the limits of a single-university, cross-sectional, self-report survey.
10. The language also needs major editing. Some terms appear inaccurate, such as “moral impact” and “Likard scale”. Several labels are inconsistent, including social place, social space, and social distribution. The manuscript needs professional academic English editing.

In summary, the topic is relevant and the dataset may be useful. However, the current manuscript does not meet Q1 or top-tier journal standards. It also lacks the scientific clarity needed for approval. A major redesign or major rewrite is required. The authors should either reframe the study as a correlational survey or collect stronger causal evidence through an intervention, longitudinal design, or experimental or quasi-experimental design.

Is the work clearly and accurately presented and does it cite the current literature?

Partly

If applicable, is the statistical analysis and its interpretation appropriate?

No

Are all the source data underlying the results available to ensure full reproducibility?

Partly

Is the study design appropriate and is the work technically sound?

No

Are the conclusions drawn adequately supported by the results?

Partly

Are sufficient details of methods and analysis provided to allow replication by others?

Partly

Reviewer Expertise:

Business, digital business, digital marketing, social marketing, consumer behaviour, small business, digital citizenship, and quantitative research methods.

I confirm that I have read this submission and believe that I have an appropriate level of expertise to state that I do not consider it to be of an acceptable scientific standard, for reasons outlined above.

# Response

**Published:** 2026-07-04

**Authors:** Reham Hamza

**Affiliations:** college, University of Fallujah, Al-Fallujah, Al Anbar Governorate, Iraq

We sincerely thank the reviewers for their valuable, constructive, and insightful comments. The suggestions provided have significantly contributed to improving the theoretical foundation, methodological rigor, and overall quality of our manuscript. We have carefully considered all comments and revised the manuscript accordingly. A point-by-point response is presented below.

**Response to Reviewer #3 — Harmon Chaniago**

 Comment #1:

 The research gap and contribution need clearer explanation.

 Response:

 We have revised the introduction section to clearly identify the research gap, emphasizing the limited integration between social marketing practices and digital etiquette within university environments, particularly in emerging contexts.

 Comment #2:

 Future research directions should be included.

 Response:

 A dedicated future research section has been added, proposing additional research opportunities such as longitudinal studies, comparative studies, and examining additional determinants of digital etiquette.

 Comment #3:

 R² values should be reported and interpreted.

 Response:

 The R² values have been added and discussed to demonstrate the explanatory power of the proposed model.

 Comment #4:

 Sampling procedures and research design require clarification.

 Response:

 The methodology section has been expanded to provide clearer details regarding research design, population, sampling procedures, and data collection.

 Comment #5:

 Statistical analysis requires improvement.

 Response:

 We have addressed the reviewer’s recommendations by improving the statistical reporting and providing additional explanations regarding the adopted analytical methods.

 Comment #6:

 The conclusions require improvement.

 Response:

 The conclusions have been revised to better reflect the empirical findings, theoretical implications, and practical contributions of the study.

# Reviewer report: Reviewer response for version 1

**DOI:** 10.5256/f1000research.191857.r487187

**Published:** 2026-06-08

**Authors:** Rahul Chauhan

**Affiliations:** 1 Karnavati University, Gandhinagar, India

Reviewer Report

 Summary

 This manuscript investigates the impact of social marketing practices on promoting digital etiquette among students at the University of Fallujah, Iraq. Using a questionnaire-based survey of 215 students, the authors examine the influence of four dimensions of social marketing (social product, social pricing, social promotion, and social distribution/place) on digital etiquette. The study addresses an important and timely topic given the increasing digitalization of higher education and the growing need to encourage responsible online behavior among university students.

 The manuscript contributes to the emerging literature on digital citizenship and social marketing in educational settings. However, several methodological, analytical, and reporting issues should be addressed before the findings can be considered sufficiently robust.

 Major Comments

1. Sampling and Representativeness

The manuscript states that a comprehensive inventory method was used; however, the total population of Fallujah University students is not reported. Consequently, it is difficult to assess whether the sample of 215 students is representative of the target population. The response rate should also be reported.

1. Statistical Analysis

The authors report that the Kolmogorov-Smirnov test indicated non-normal distributions for the main variables (p < 0.05). Despite this finding, Pearson correlation and regression analyses were applied without sufficient justification. The authors should explain why parametric methods were considered appropriate under these conditions or consider reporting robustness checks using non-parametric techniques.

1. Construct Validity

Although confirmatory factor analysis (CFA) was conducted, important validity indicators are not reported. The manuscript would be strengthened by including Composite Reliability (CR), Average Variance Extracted (AVE), and measures of discriminant validity. These statistics are commonly expected in studies involving latent constructs.

1. Inconsistency in Demographic Reporting

The gender percentages reported in the manuscript appear inconsistent, as males account for 55% and females account for 54%, resulting in a total exceeding 100%. The authors should carefully verify all demographic statistics and percentages.

1. Theoretical Framework

The study lacks a clearly articulated theoretical framework explaining how social marketing practices influence digital etiquette. The inclusion of an established theory such as the Theory of Planned Behavior, Social Cognitive Theory, Digital Citizenship Theory, or a formal Social Marketing framework would strengthen the conceptual foundation of the study.

1. Reporting of Effect Size

The manuscript focuses primarily on statistical significance. Measures of explanatory power such as R², adjusted R², and effect sizes should be reported and discussed to provide a clearer understanding of the practical significance of the findings.

1. Interpretation of Findings

Some questionnaire items require further justification. For example, the item relating to social isolation within the health dimension appears conceptually inconsistent with positive digital etiquette and may require reconsideration or further explanation.

 Minor Comments

1. The manuscript would benefit from professional English language editing. Several grammatical errors and awkward expressions are present throughout the text.
2. Terminology should be standardized. The manuscript alternates between "social place," "social distribution," and "social space," which may confuse readers.
3. Some figure captions contain repetitive wording such as "explains illustrates." These should be revised for clarity.
4. The literature review would benefit from greater synthesis and a clearer identification of the research gap addressed by the study.
5. More detailed information regarding questionnaire development, pilot testing, and content validity procedures should be included.

Data Availability

 The authors have provided access to the underlying dataset through Zenodo, which supports transparency and reproducibility. This is a positive aspect of the study.

 Conclusions

 The conclusions are generally consistent with the reported findings. However, because of concerns regarding sampling, construct validation, statistical justification, and reporting transparency, the conclusions should be interpreted with caution until these issues are addressed.

 Recommendation

 Major Revision

 The study investigates a relevant topic and has potential value for higher education and digital behavior research. Nevertheless, substantial revisions are required to strengthen the methodological rigor, reporting quality, and theoretical grounding of the work before it is suitable for indexing.

Is the work clearly and accurately presented and does it cite the current literature?

Partly

If applicable, is the statistical analysis and its interpretation appropriate?

Partly

Are all the source data underlying the results available to ensure full reproducibility?

Yes

Is the study design appropriate and is the work technically sound?

Partly

Are the conclusions drawn adequately supported by the results?

Partly

Are sufficient details of methods and analysis provided to allow replication by others?

Partly

Reviewer Expertise:

Marketing, Social Marketing, Consumer Behaviour, Digital Marketing, Educational Technology, Higher Education Research, Research Methodology, Quantitative Data Analysis, Structural Equation Modeling (SEM), Behavioral Studies, and Management Research.

I confirm that I have read this submission and believe that I have an appropriate level of expertise to confirm that it is of an acceptable scientific standard, however I have significant reservations, as outlined above.

# Response

**Published:** 2026-07-04

**Authors:** Reham Hamza

**Affiliations:** college, University of Fallujah, Al-Fallujah, Al Anbar Governorate, Iraq

We sincerely thank the reviewers for their valuable, constructive, and insightful comments. The suggestions provided have significantly contributed to improving the theoretical foundation, methodological rigor, and overall quality of our manuscript. We have carefully considered all comments and revised the manuscript accordingly. A point-by-point response is presented below.

**Response to Reviewer #2 — Rahul Chauhan**

 Comment #1:

 The theoretical contribution remains limited, and the study needs a stronger explanation of why social marketing is an appropriate framework for understanding digital etiquette.

 Response:

 We have strengthened the theoretical contribution by clarifying the research gap and explaining why social marketing provides an appropriate framework for promoting responsible digital behavior. The revised manuscript highlights the role of social marketing practices in shaping awareness, attitudes, and behavioral intentions related to digital etiquette.

 Comment #2:

 The literature review is mainly descriptive and requires stronger critical comparison with previous studies.

 Response:

 We revised the literature review by adding a more critical discussion of previous studies, identifying inconsistencies and unexplored areas, and emphasizing the contribution of the current study to digital behavior and social marketing literature.

 Comment #3:

 The sampling procedure requires further clarification.

 Response:

 Additional details regarding population size, sampling procedure, inclusion criteria, exclusion criteria, and sample determination have been added.

 Comment #4:

 The statistical analysis should report standardized coefficients, R², effect sizes, VIF, and model diagnostics.

 Response:

 We have incorporated the recommended statistical indicators, including explanatory power measures and additional diagnostics where appropriate, to improve the transparency and robustness of the analysis.

 Comment #5:

 The confirmatory factor analysis reporting is incomplete.

 Response:

 We have revised the measurement model reporting and clarified the estimation method used, considering the distribution characteristics of the collected data. Additional validity evidence has been provided.

 Comment #6:

 Common method bias assessment requires improvement.

 Response:

 We have addressed this issue by expanding the discussion of common method bias and adding appropriate procedural and statistical considerations. The limitation related to single-source and cross-sectional data collection has also been acknowledged.
